# An Alternative Strategy for Screening and Confirmation of 330 Pesticides in Ground- and Surface Water Using Liquid Chromatography Tandem Mass Spectrometry

**DOI:** 10.3390/molecules27061872

**Published:** 2022-03-14

**Authors:** Edgár Tóth, Ádám Tölgyesi, Andrea Simon, Mária Bálint, Xingmao Ma, Virender K. Sharma

**Affiliations:** 1 Bálint Analitika Ltd., Fehérvári út 144, 1116 Budapest, Hungary; ttedgar90@gmail.com (E.T.); schimocza@gmail.com (A.S.); hplc@balintanalitika.hu (M.B.); 2 Department of Civil and Environmental Engineering, Texas A&M University, College Station, TX 77843, USA; samuelma@tamu.edu; 3 Program for the Environment and Sustainability, Department of Environmental and Occupational Health, School of Public Health, Texas A&M University, 212 Adriance Lab Road, 1266 TAMU, College Station, TX 77843, USA

**Keywords:** pesticides, water, solid-phase extraction, LC-MS/MS, validation

## Abstract

The presence of pesticide residues in water is a huge worldwide concern. In this paper we described the development and validation of a new liquid chromatography tandem mass spectrometric (LC-MS/MS) method for both screening and quantification of pesticides in water samples. In the sample preparation stage, the samples were buffered to pH 7.0 and pre-concentrated on polymeric-based cartridges via solid-phase extraction (SPE). Highly sensitive detection was carried out with mobile phases containing only 5 mM ammonium formate (pH of 6.8) as an eluent additive and using only positive ionization mode in MS/MS instrument. Hence, only 200-fold sample enrichment was required to set a screening detection limit (SDL) and reporting limit (RL) of 10 ng/L. The confirmatory method was validated at 10 and 100 ng/L spiking levels. The apparent recoveries obtained from the matrix-matched calibration (5–500 ng/L) were within the acceptable range (60–120%), also the precision (relative standard deviation, RSD) was not higher than 20%. During the development, 480 pesticides were tested and 330 compounds fulfilled the requirements of validation. The method was successfully applied to proficiency test samples to evaluate its accuracy. Moreover, the method robustness test was carried out using higher sample volume (500 mL) followed by automated SPE enrichment. Finally, the method was used to analyze 20 real samples, in which some compounds were detected around 10 ng/L, but never exceeded the assay maximum level.

## 1. Introduction

To protect crops from pathogens, fungi, weeds and insects, millions of tons of pesticides are applied each year in order to maintain agricultural productivity [1,2,3,4]. Herbicides and insecticides are the most common pesticides used, accounting for about 47.5% and 29.5% of the total pesticide consumption [1,5]. So far, hundreds of pesticides are registered in the European Union (EU) and most of them have maximum residue limits (MRLs) in foods of plant origin to protect consumers against toxic chemicals [6]. While many of these chemicals are readily degraded in the environment or have no adverse effects on human health, there are some for which there is little toxicological information [2,3,4,5,7,8,9]. The extensive use of pesticides, however, also means that they appear and accumulate in soil, clay or in water samples based on the hydrophobicity of the molecules [2,3,4,10]. 

A recent Hungarian study on pesticides in ground- and surface water reported that more than half samples contained detectable levels of pesticides, and about 5% of the samples contained pesticides at levels exceeding the 100 ng/L maximum concentration level for individual pesticides, and 500 ng/L maximum concentration level for the sum of pesticides as specified by EU directives [11,12,13]. Additionally, numerous research articles have reported the frequent detection of pesticides in water worldwide [2,3,4,5,7,8,9,14,15,16,17,18,19,20,21,22,23,24]. The other environmental concern is the fate of pesticides, which can lead to harmful metabolites with unknown toxicity to animals and humans [3,8,9]. According to Bexfield et al. [25], among the 41% of groundwater containing pesticide compounds, three-quarters contained metabolites as well. Therefore, there is a great need to monitor pesticide residues in water samples using state-of-the-art analytical techniques that enable the simultaneous analysis of high numbers of compounds [2]. 

In food analysis, the EN 15662:2018 standard, utilizing QuEChERS (quick, easy, cheap, effective, rugged and safe) sample preparation, describes the detection of hundreds of pesticide compounds [26]. However, the target concentration levels in food are much higher than 100 ng/L, the individual maximum level of pesticides in water [12,13]. Consequently, sample pre-concentration should be carried out during sample preparation before instrumental analysis [2,18,19,20,21]; however, the direct injection of water samples has been used in some published methods [14,15,16]. Gas chromatography (GC) or ultra/high performance liquid chromatography (UHPLC) coupled with mass spectrometric detection (GC-MS or LC-MS) are the most suitable techniques for the analysis of a high number of pesticides [2,3,4,11,17,26,27,28]. From an analytical point of view, a great number of pesticides is not necessarily indicative of structural diversity. At present, a universal method for pesticide pre-concentration and analysis in water is still lacking. There will always be a loss of compounds under sample extraction due to their highly polar structure. Additionally, both GC and HPLC analysis may be used to cover as many compounds as possible. In recent papers, the detection of 20–300 pesticides in water could be achieved if both GC-MS and LC-MS were used in the method [5,7,8,9,18,19,20,21,29,30,31,32]. For GC-MS analysis, the liquid–liquid extraction (LLE) of water with immiscible organic solvent such as diethyl ether or dichloromethane is a preferable approach to enrich pesticides [2,27,33,34]. This sample pre-concentration method is quite selective and is a good tool to pre-concentrate more volatile compounds; however, it requires a high volume of organic solvent that does not meet the purpose of green analytical chemistry [35]. The extraction of more hydrophilic compounds with LLE results in losses during sample preparation, therefore solid-phase extraction (SPE) shall be employed to obtain fit-for-purpose recovery for a broader range of analytes [2,18,19,20,21,29,30,31,36,37]. SPE utilizing modified hydrophilic polymeric cartridges enables the extraction of both polar and non-polar compounds if the sample pH is optimal [22,23,24,28,33,36,37]. Our aim was to develop an SPE clean-up method that enables the analysis of as many pesticides as possible in water. In our laboratory, 480 compounds are detected in food with EN 15662:2018 standard [26] using LC-MS/MS technique and these compounds (Table S1) were in the focus of the present study.

In this paper, we report for the first time the simultaneous separation of more than 300 pesticides in ground- and surface water using a simple sample enrichment and LC-MS/MS separation. Our goal was to develop a LC-MS/MS method that enables fast screening of as many pesticides as possible employing polymeric SPE pre-concentration at an optimized sample pH. Furthermore, our aim was to use the method for confirmatory purposes, so the validation of the method in line with the screening and confirmatory requirements is carried out including the proficiency test evaluation. Another aim was to verify the method’s robustness by automating the SPE process and using a higher sample volume. Finally, the method was applied to real samples to monitor pesticide contamination. 

## 2. Results

### 2.1. SPE-LC-MS/MS Method

The LC-MS/MS separation for 480 compounds in neat solvent (Figure S1 and Table S1) was developed for the analysis of large numbers of pesticides in food samples according to the EN 15662:2018 standard [26]. Our deviation from the standard method in terms of compound separation was the elimination of acid from the mobile phases and application of C_18_ HPLC column packed with fused-core particles. Although only positive ionization was used, the introduction of acid in the eluents (0.1%, *v*/*v*, HCOOH) did not improve the sensitivity, instead, it decreased the intensity of adduct precursor ions other than [M + H]^+^. Moreover, the intensity of some compounds detected with protonated molecules also decreased with a factor of 2–10. These compounds include, for instance, acetamipirid, acetochlor, furalaxyl and fluazuron. Only a few compounds had higher sensitivity with acidic eluent such asiprolidone, pirimiphos-ethyl and pirimiphos-methyl. Only 2-fold enhancement was observed. This was also found in our previous work using the same instrument [38]. The neutral eluent composition allowed an instrumental limit of quantification (LOQ) ≤1 μg/L in neat solvent. The improved sensitivity caused by the non-acidified eluents allowed the detection of 480 compounds without polarity switching in the ion source (Table S1). These 480 compounds were tested for SPE pre-concentration using hydrophilic modified polystyrene and divinylbenzene cartridges (Strata-XL) in ground- and surface water. 

The sample pH was found to be critical because pesticides with various chemical structures (acidic, neutral, basic) were analyzed; on the other hand, pesticides can appear in different chemical forms in environmental water as metal complex [39], hence EDTA at 10 μg/mL was added to all samples to convert complexes into free form of pesticides because metal ions complex preferably with EDTA [39]. Three pH conditions were tested: acidification with formic acid (at pH 3 and at pH 5) and neutral condition (pH 7) using QuEChERS extraction salt prepared according to EN 15662:2018 standard [26]. The acidic condition is quite important for phenoxy herbicides that have an acidic character [26]; however, the acidification resulted in a considerable loss of more than half of the compounds compared to the neutral condition. The SPE cartridge had no retention for phenoxy herbicides above pH 5 [24]. Only matrix-matched calibration and isotope dilution could improve the recovery, approximately, two-thirds of the analytes did not reach the recovery limit of 60% either at pH of 3 or 5. Additionally, the RSD% was higher than 20% for most of the compounds evaluated from three ground- and three surface water samples spiked at 100 ng/L. This led to the trial of buffered water using QuEChERS extraction salt. The dissolution of 6.5 g salt in 100 mL water supersaturated the sample that would precipitate the non-polar compounds before SPE. Therefore, the amount of salt was reduced to obtain easy dissolution of salt mix in water at the pH of 7. This was achieved by dissolving 6.5 g salt in 500 mL water. Since we aimed to obtain a fast (rapid) procedure, the sample volume was reduced to 100 mL, and only 1.3 g salt was added. This addition of 1.3 g salt in water did not precipitate the compounds because all 480 compounds were detected by direct injection of 10 μL buffered water spiked at 1 μg/L level. Among them, 150 compounds did not fulfill the 60–120% recovery and RSD of 20%. They might have been lost during the SPE pre-concentration. The SPE enrichment of the buffered sample at pH 7 significantly improved the recovery and the precision for most compounds. Using neutral condition, the analytical performance of 330 pesticides, evaluated with matrix-matched calibration solutions, reached the requirement of recovery 60–120% and RSD ≤ 20% calculated from three spikes at 100 ng/L. Moreover, the signal-to-noise ratio (SNR) at 100 ng/L level was not lower than 100, suggesting that the LOQ/SDL/RL ≤ 10 ng/mL can be achieved with 200-fold pre-concentration of the sample. Those compounds that were excluded due to their low recovery (<60%) are highlighted in Table S1.

### 2.2. Method Validation

Two kinds of validation were performed: screening and confirmatory [40,41]. The point of screening method is to distinguish the compliant samples as fast as possible from those containing pesticides above screening detection limit (SDL). Overall, a 10-fold lower concentration than the maximum level (100 ng/L) was set as the SDL concentration (10 ng/L). The SNR, calculated for the selected 330 compounds from the 20 spiked samples analyzed at 10 ng/L, was always higher than 10. Interfering peaks within the compounds’ retention time windows were not detected; therefore, the 20 spikes could be distinguished from the 20 blanks at 10 ng/L. This means that samples at 10 ng/L concentration can be detected with the SPE-LC-MS/MS method with an error of 5% [41]. Some of the compounds could even be detected at lower levels (0.1–10 ng/L); however, 10 ng/L was the concentration that can be detected after 200-fold sample enrichment for all compounds in all investigated real samples. The CEN/TS 17061:2017 standard allows the quantification of pesticides using one-point calibration [40,41]. In this case, a semi-quantitative result can also be obtained from the screening analysis that supports the confirmatory analysis in terms of the calibration range used.

**Figure 1:**
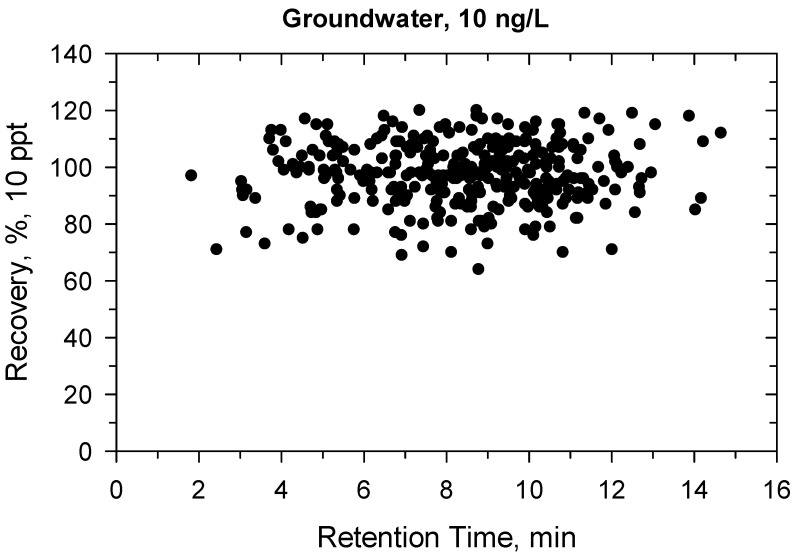
Recovery (%) versus retention time (min) at 10 ng/L in groundwater.

**Figure 2:**
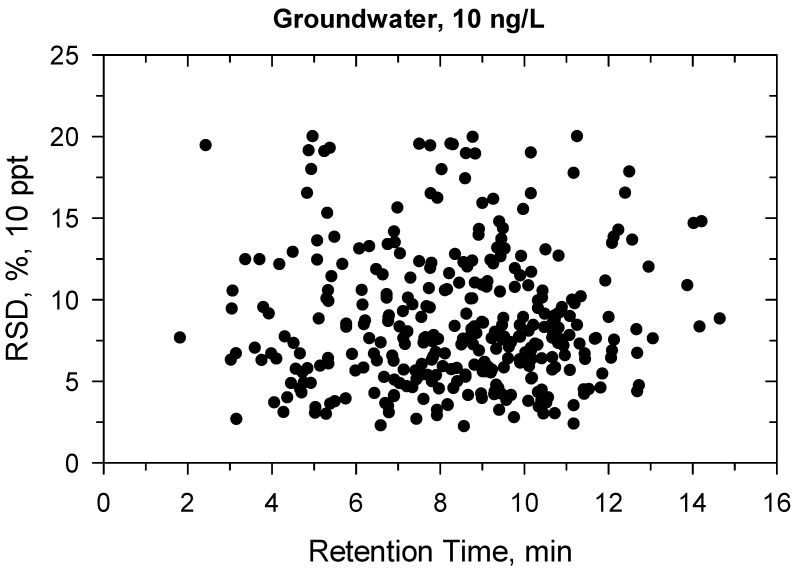
Precision (%) versus retention time (min) at 10 ng/L in groundwater.

**Figure 3:**
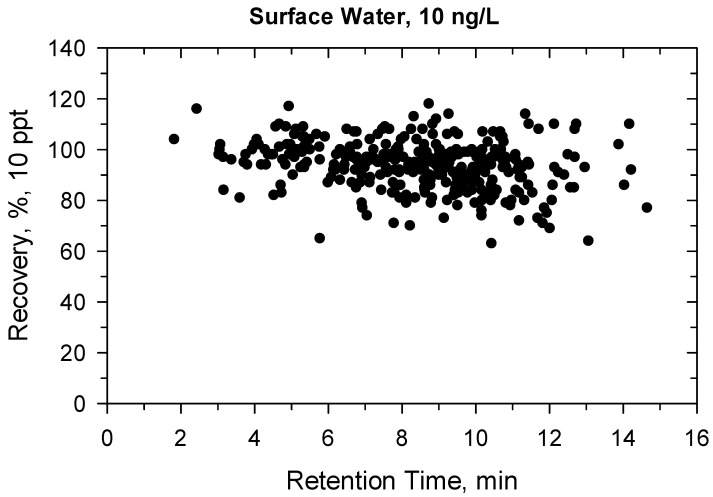
Recovery (%) versus retention time (min) at 10 ng/L in surface water.

**Figure 4:**
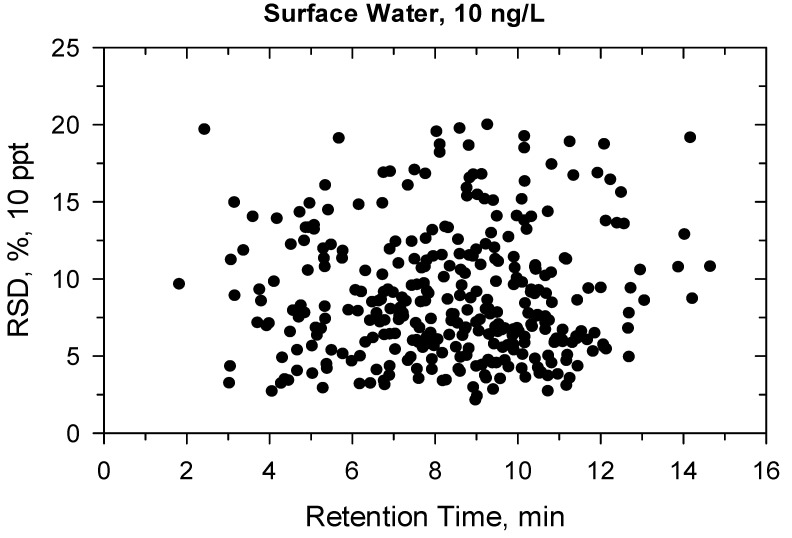
Precision (%) versus retention time (min) at 10 ng/L in surface water.

Under confirmatory validation, the matrix-matched calibration from 5 to 500 ng/L showed fit-for-purpose linear regression. Above 500 ng/L, the regression became quadratic, so the linear range was found ≤500 ng/L. Two ion transitions were used for each compound (Supplementary Table S1) and all ion traces could be detected at 10 ng/L, the ion ratios were within the permitted tolerance range (±30%), hence the identification criteria met the requirement [41]. The recovery and precision data for the 330 compounds are detailed in Table S2a,b and shown in Figure 1, Figure 2, Figure 3 and Figure 4. All recoveries were between 60 and 120% calculated from 10 samples for each matrix (ground- or surface water) and at both spike levels (10–100 ng/L). Additionally, the RSD was not higher than 20% for all compounds (Table S2a,b). Figure 1, Figure 2, Figure 3 and Figure 4 shows how the results at 10 ng/L depend on the retention time of compounds (Figures S2–S5 shows the charts at 100 ng/L spiking level). Lower retention means more polar compounds, so the validation data versus hydrophobicity of pesticides is plotted. The recoveries randomly deviated from 100%, significant trend based on retention times could not be seen. The same conclusion is true for the precision. Those compounds had better recoveries and high precision whose losses during sample preparation or in the ion source were compensated with isotope dilution (Tables S1 and S2a,b).

The SNR was higher than 10 for all compounds at 10 ng/L level, so this concentration was set as the reporting limit (RL) for all compounds. The LOQ varied between compounds, some pesticides had very high sensitivity, but all compounds could be detected at 10 ng/L. Hence, we preferred the introduction of RL rather than LOQ. The validation results met the requirements [41]. The accuracy and robustness were further investigated by a proficiency test and by testing the SPE enrichment with an automated system (see below).

### 2.3. Proficiency Test

**Figure 5:**
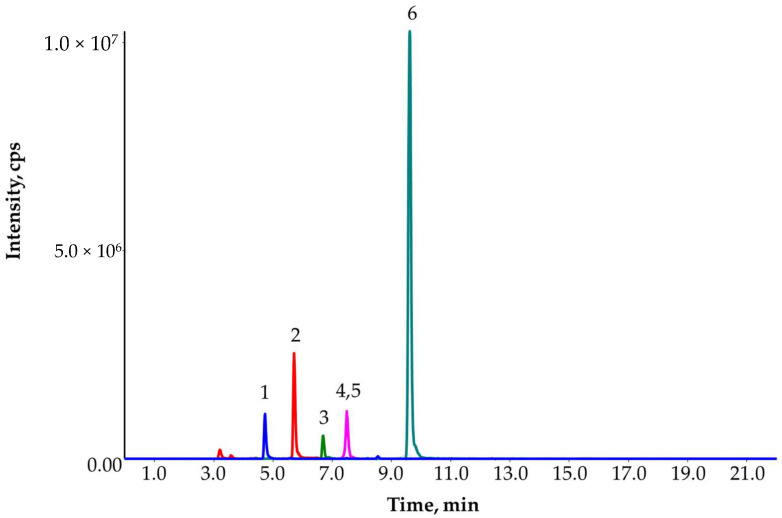
Extracted ion chromatogram of the first PT sample containing: desizopropyl atrazine (1), desethyl atrazine (2), simazine (3), metazachlor (4), atrazine (5) and metolachlor (6). The metazachlor (4) and atrazine (5) completely co-eluted in the chromatogram with the same intensity. However, the MS instrument could distinguish them on different mass channels, so interference did not influence the analysis.

The results of the proficiency test (PT) are shown in Table 1. Two PT samples were received for analysis. First, they were screened for 330 compounds at 10 ng/L. The detected compounds were: atrazine, desethyl atrazine, desisopropyl atrazine, metazachlor, metolachlor and simazine (Figure 5). All concentrations were higher than the SDL (10 ng/L). During the confirmatory analysis, samples were prepared in duplicate and evaluated with matrix-matched calibration together with isotope dilution (Section 4.5). The average concentrations were reported.

Both the identification and quantification were satisfactory (Table 1). In the PT report, the organizers only provided charts in which the deviations from the target values could be seen. The permitted deviation was ±35% for all compounds and in both samples. In sample 2, the desisopropyl atrazine concentrations submitted by participants had high standard deviation and limited data were available, so the evaluation of desisopropyl atrazine was inconclusive. All submitted results were satisfactory, the accuracy of the method was fit for purpose.

### 2.4. Automated SPE Pre-Concetration

The method’s robustness was tested with automated SPE enrichment using the same extraction method and with a 500 mL groundwater sample (Section 4.7). In this case, the pre-concentration factor was 1000-fold and that considerably improved the analytical limits which decrease with higher enrichment ratio, meaning that the noise level did not increase between 200- and 1000-fold pre-concentration. The calculation of recovery and precision from the six spikes (10 ng/L) using the single calibration point (100 ng/L) showed that the compounds could be recovered within the acceptable range (60–120%). Moreover, the recovery was between 80 and 120% (Table S3), while the RSD% remained lower than 20% which is even better than the results obtained with manual SPE (Table S2a,b). It should be pointed out that this trial was performed with only six samples and at one day. The conclusion is that SPE automation did not lower the method performance and the automatization of SPE enrichment is feasible.

### 2.5. Real Sample Analysis

**Figure 6:**
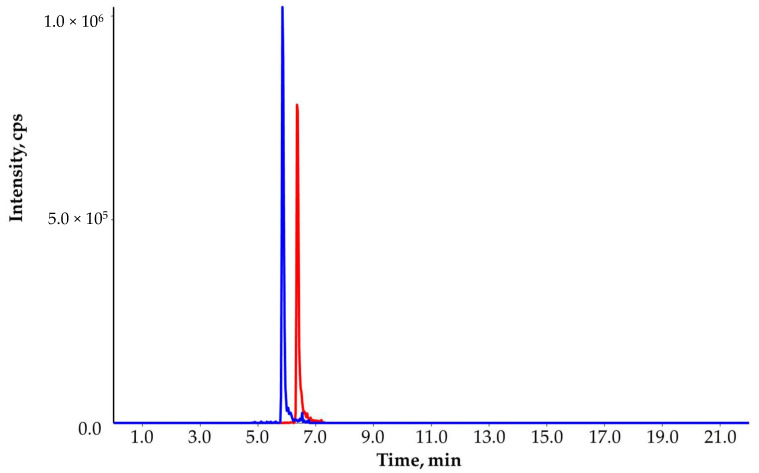
Extracted ion chromatogram of carbendazim (10.3 ng/L; blue line) and thiabendazole (10.4 ng/L; red line) in the surface water #8.

One groundwater and nineteen surface water samples were first screened: all samples were analyzed with and without spiking (10 ng/L). The detected compounds in real samples above 10 ng/L were: acetamiprid, atrazine-desethyl, azoxystrobin, boscalicid, carbendazim, fenhexamid, imidacloprid, isoproturon, metolachlor, penconazole, tebuconazole, terbuthylazine-desethyl and thiabendazole (Table 2). Then, samples were re-analyzed with the confirmatory method to verify the identified compounds and calculate their concentrations. Results are summarized in Table 2. All identified compounds were confirmed, and the concentration ranged from 10.1 to 43.9 ng/L. The most frequently detected compounds were carbendazim and thiabendazole (Figure 6). Neither the individual concentration nor the sum of concentrations exceeded the maximum levels (100 and 500 ng/L). Furthermore, those compounds, which could be detected with appropriate SNR and ion ratio below 10 ng/L, were also evaluated during the confirmatory analysis. All investigated and confirmed compounds and concentrations are summarized in Table S4. This investigation highlighted that the total concentration of pesticides in water can be significantly different if only those results that are higher than the RL of 10 ng/L (Table 2) are considered. For instance, the 18th surface water contained 129 compounds between 1 and 10 ng/L, which increased the sum concentration from 33.2 to 245 ng/L. The most frequently detected compounds below 10 ng/L were the atrazine and azoxystrobin (Table S4).

## 3. Discussion

### 3.1. Method Development

In general, both GC-MS/MS and LC-MS/MS are required to monitor concerned pesticides in water [2,26]. If only the LC-MS/MS technique is used, the polarity switching in the ion source limits the number of compounds that can be detected [26]. Our aim was to only use LC-MS/MS with positive ionization. The acidification of mobile phase is commonly used to improve the sensitivity in positive ionization mode [2]; however, our instrument did not show considerable sensitivity enhancement for all compounds with acidic eluents. Those compounds that were detected with sodium or ammonium adduct precursor ions had the highest sensitivity drop with acidic eluents. The used LC-MS/MS instrument does not require acidic condition for higher sensitivity, consistent with our previous work [38]. The mobile phase consisting only of ammonium formate as eluent additives enabled the simultaneous analysis of 480 pesticides using scheduled multiple reaction monitoring (MRM) detection in the MS/MS instrument (Table S1). This eluent composition is rather different from those used earlier [2,14,15,16,18,19,20,21,26,29,30,31,32].

Even though 480 compounds were tested, only 330 analytes fulfilled the minimum requirements of analytical performance characteristics (Table S1) due to the diverse properties of pesticides. The poor detection of some pesticides could be caused by the hydrophobicity of these compounds. The retention of chemicals on SPE cartridge is influenced by the sample composition and pH. Since, the acidic condition resulted in higher compound loss, the phenoxy herbicides could not be analyzed by the method. Instead, these compounds require negative ionization [24]. Under acidic conditions, the basic target compounds are in a more polar chemical form and had lower retention on the cartridge, while the neutral and acidic pesticides could be well extracted at lower pH.

**Figure 7:**
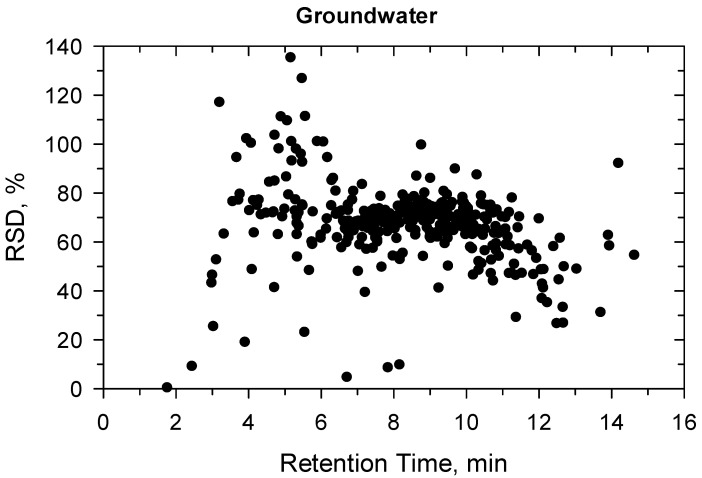
Recovery (%) versus retention time (min) at 10 ng/L in groundwater. Results were calculated without matrix compensation.

**Figure 8:**
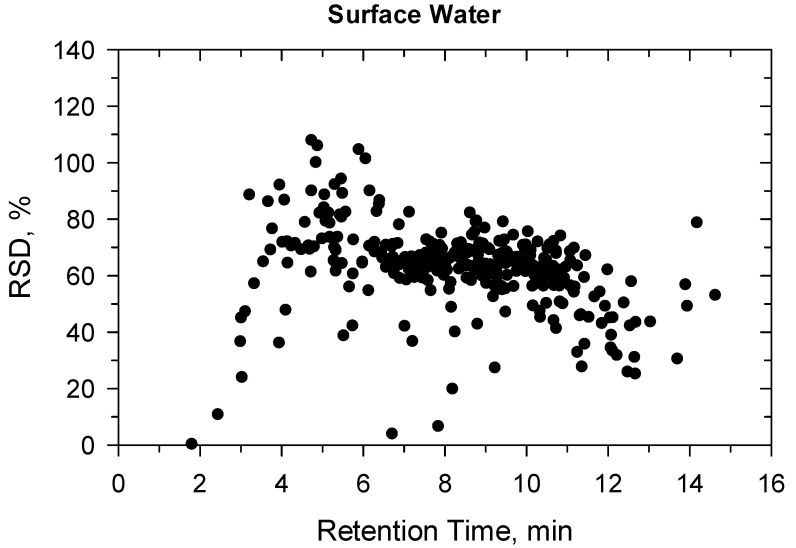
Recovery (%) versus retention time (min) at 10 ng/L in surface water. Results were calculated without matrix compensation.

The more polar compounds had lower retention on SPE cartridge, while the more hydrophobic ones had lower solubility either in the sample solvents or in the injection solution. This was confirmed by plotting the compound retention time versus recovery evaluated with external calibration without matrix compensation (Figure 7 and Figure 8). The best recoveries could be achieved for medium polar compounds (from pyridafol, Rt = 3.93 min, LogP = 0.56 to diflubenzuron, Rt = 9.98 min, LogP = 3.89) that elute in the retention time window from 4 to 10 min, in both ground- and surface water spiked at 10 ng/L. There is a sharp improvement in recoveries between 2 and 4 min as the more non-polar compounds elute. After 10 min, where the most non-polar compounds elute, the recovery starts to drop. It should be mentioned that the lower recoveries (≤60%) can also be caused by non-compensated background that suppresses the compounds’ signal in the ion source. After background compensation with matrix-matched calibration and internal standard (IS), these 330 compounds could be recovered between 60–120% (Table S2a,b). For instance, methamidophos (elutes at 1.9 min) had almost <5% recovery without IS (methamidophos-d6) compensation, while the IS evaluation resulted in fit-for-purpose recovery and precision in both method validation and automation SPE experiments (Tables S2a,b and S3). The results suggest that more compounds might be included if all ISs are available, but this would significantly increase the cost, so it is not effective.

This solubility issue for non-polar compounds was also seen when samples were filtered through inert hydrophilic PTFE syringe filter after sample re-constitution. Most of the non-polar compounds fully disappeared from the sample solution. Therefore, those samples, which remained turbid after SPE clean-up, were only centrifuged in plastic Eppendorf tubes. The other source of compound loss was the evaporation step. The evaporation to dryness, followed by sample re-constitution in 50% MeOH reduced the number of non-polar compounds, again. Therefore, the SPE eluates were evaporated only to 0.5 mL, in which the composition of the sample solvent allowed the injection of 5 μL sample without distorting the chromatographic peak of hydrophilic compounds.

In Table 3, we summarized some earlier methods that only utilized LC-MS separation as well. High number of pesticides (300) could be detected if direct injection was applied. The direct injection needs an injector loop with high volume (i.e., 400 μL) that is not common; moreover, the non-purified samples can considerably decrease the lifetime and resolution of HPLC column and the instrument, and reduces the sensitivity of detection if high numbers of samples are analyzed in a sequence. Our method is capable of simultaneously separating 330 pesticides in water samples at level ≤10 ng/L after a simple and fast SPE pre-concentration.

### 3.2. Real Sample Analysis

The real sample analysis mostly covered surface water that is generally more polluted with pesticides [5,7,8,24]. The one groundwater tested did not contain pesticide above 10 ng/L (Table 2), but it did contain detectable pesticides between 1 and 10 ng/L. In total, 16 out of 19 surface water samples contained pesticides above 10 ng/L (Table S4). The daily condition of the instrument and the complexity of samples can also influence the sensitivity of the detection; however, 10 ng/L was the concentration that could be detected for all compounds in any scenario using 200-fold sample pre-concentration. Therefore, the set SDL and RL (both 10 ng/L) were fit for purpose and the further lowering of them is not necessary. This level can also be achieved with a less sensitive instrument if higher enrichment is applied.

Concentrations below 1 ng/L were also detectable for some compounds, but these compounds do not impact the total concentration of pesticides; therefore, the concentration below 1 ng/L was considered as non-detected (nd) in Table S4. On the other hand, the large number of pesticides, found between 1 and 10 ng/L, could considerably increase the total concentration of pesticides (Table 2). This can cause various sum concentrations of pesticides in a particular sample between laboratories if other laboratories have different RLs. In this case, only the individual concentrations, higher than RL/LOQ, can be compared. Lepom et al. suggested that the methods should be able to detect at least 30% of the maximum level [42] that means 30 ng/L here. The 30 ng/L as RL would mean that only a few pesticides are reported from the 20 samples, which can be a significant underestimation of the individual and total concentrations of pesticides in water (Table S4). Most of the detected chemicals in the screened samples were between 1 and 10 ng/L, the further confirmation of them would not be necessary because the 500 ng/L maximum limit for sum concentration is quite far from their concentrations. For example, surface water sample #18 clearly shows this scenario (Table 2). The confirmation of all compounds above 1 ng/L is time consuming and not cost effective. In conclusion, there is no need to put in great effort to decrease the SDL/RL/LOQ below 10 ng/L; however, this level should be reached at least. The RL of ≤10 ng/L is achievable with the presented method. In summary, our study led to a cost-effective and sensitive LC-MS/MS method that can simultaneously detect over 300 pesticide compounds with limited pretreatment.

## 4. Materials and Methods

### 4.1. Reagents and Samples

Both native and isotopically labelled standards (acetamiprid-d3, atrazine-d5, atrazine-desisopropyl-d5, boscalid-d4, carbaryl-d7, carbendazim-d3, chlorotoluron-d6, cyromazine-d4, diazinon-d10, dichlorvos-d6, difenoconazole-d4, diflufenican-d3, dimethoate-d6, fipronil-^13^C3, fluopyram-d4, imidacloprid-d4, malathion-d6, methamidophos-d6, metolachlor-d6, napropamide-d10, pendimethalin-d5, pyraclostrobin-d3, pyrimethanil-d5, tebuconazole-^13^C3, thiabendazole-d4 and thiametoxam-d3) were obtained from LGC (Wesel, UK). Stock solutions (1 mg/mL) were prepared and stored according to the pesticide database [6]. Working standard mixture solution including the native standards for calibration and spiking purpose was prepared at 100 ng/mL in acetonitrile at −20 °C for a week. Additionally, an internal standard (IS) mixture solution containing the labelled analogues was prepared at 100 ng/mL in acetonitrile and stored under the same conditions.

Methanol, acetonitrile, ammonium formate, formic acid (either LC-MS or HPLC grade), ethylenediaminetetraacetic acid disodium salt dihydrate (Na_2_-EDTA × 2H_2_O) and the Ascentis Express C_18_ HPLC column (100 mm × 3 mm, 2.7 μm) were purchased from the Merck-Sigma group (Schnelldorf, Germany). EN 15662:2018 QuEChERS extraction salt was obtained from Agilent Technologies (Waldbronn, Germany) that contains 4 g MgSO_4_, 1 g NaCl, 1 g Na-citrate × 2H_2_O and 0.5 g Na-hydrogencitrate sesquihydrate. HPLC gradient grade water was obtained from VWR International Ltd. (Debrecen, Hungary). HPLC pre-column holders and C_18_ pre-column cartridges (4 mm × 3 mm; 5 μm) and Strata-XL SPE cartridges (6 mL/200 mg) were obtained from Phenomenex (Torrance, CA, USA). Hydrophilic PTFE syringe filters were obtained from Gen-Lab Ltd. (Budapest, Hungary).

The twenty real samples (one ground- and nineteen surface water) were collected by our accredited sampling department in November 2021 and were stored at +4 °C in the dark until analyses. Samples originated from the Zala region of Hungary. Proficiency test groundwater samples were obtained from Nemzeti Népegészségügyi Központ (Budapest, Hungary).

### 4.2. Instrumentation

LC-MS/MS analyses were carried out on a Shimadzu Nexera LC-30AD liquid chromatograph, consisting of a SIL-30AC auto sampler, CTO-20AC column oven and CBM-20A communications bus module (Shimadzu Corporation, Kyoto, Japan), coupled to a QTRAP 6500+ triple quad MS detector equipped with an IonDrive Turbo V Source ( Sciex; Warrington, Cheshire, UK). Data acquisition and evaluation were performed with the Analyst software version 1.7.1 and MultiQuant software version 3.0.3, respectively(Sciex; Warrington, Cheshire, UK). Automated SPE clean-up was performed using SPE-03 PromoChrom 8-Channel High Volume Automated SPE system (PromoChrom Technologies, Richmond, BC, Canada). Sample evaporation was carried out by TurboVap II (Biotage, Uppsala, Sweden). MicroCen MR Eppendorf centrifuge was obtained from Herolab GmbH (Wiesloch, Germany).

### 4.3. Sample Preparation

Water samples were filtered on Macherey–Nagel (MN 615 ¼, 240 mm) filter paper (Düren, Germany,) into glass bottles. QuEChERS extraction salt (1.3 g) and 10 μL 10% (m/m) Na_2_-EDTA (pH 7) was added to 100 mL filtrate. Samples were mixed with 100 μL IS mix solution (100 ng/mL), corresponding to 100 ng/L in the samples. Then, samples were subjected to SPE clean-up.

Strata-XL SPE cartridges (200 mg/6 mL) were attached to a vacuum manifold and conditioned with 6 mL methanol, followed by 6 mL water. The dropping was not aided with vacuum and was solely due to the gravity. Samples were passed through dropwise, then cartridges were rinsed with 6 mL water. Afterwards, cartridges were dried with vacuum for 1 min before elution. The drying steps did not affect the performance of SPE pre-concentration in terms of recovery and precision because the cartridges were polymeric based. Samples were eluted with 5 mL methanol into glass tubes. Eluates were evaporated to 0.5 mL at 45 °C under a gentle stream of nitrogen. If needed, the volume of the evaporated eluate was adjusted to 0.5 mL with 50% methanol. Tubes were vortex-mixed for 10 s, and samples were then transferred into HPLC vials. Turbid samples were centrifuged in Eppendorf tubes at 10,000 rpm at ambient temperature for 1 min. The syringe filtration was eliminated because it caused significant losses of some non-polar compounds. The dilution factor was 0.005 (200-fold pre-concentration).

### 4.4. LC-MS/MS Separation

Pesticides were separated on an Ascentis Express C_18_ HPLC column equipped with C_18_ guard column (4 mm × 3 mm, 5 μm). Binary gradient elution mode was applied with solvent A containing 5 mM ammonium formate in water and solvent B containing 5 mM ammonium formate in methanol. The mobile phase gradient consisted of 10% B at 0 min; 10% B at 1.0 min; 62% B at 5.5 min; 100% B at 14 min; 100% B at 17 min; 10% B at 17.1 min; 10% B at 22.0 min; and flow rate was set to 0.5 mL/min. The column thermostat and autosampler were maintained at 30 °C and at 15 °C, respectively. The injection volume was 5 μL. Compounds were detected using positive ionization mode and scheduled multiple reaction monitoring (sMRM) scan mode. Ion transitions for 480 compounds are presented in Supplementary Table S1. The MRM time window was 120 ms and the cycle time was 0.400 s. ESI ion source parameters were as follows: curtain gas 40 unit, gas1 50 unit, gas2 65 unit, drying gas temperature 300 °C and ion spray voltage: 4500 V and interface heater ‘on’. The HPLC effluent entered the ion source only in the retention time window between 1.5 and 15 min.

### 4.5. Quantification

In the case of screening analysis, samples were extracted with and without spiking. Both samples were spiked with 100 ng/L IS mixture, but only the second sample was fortified with native working standard solution to obtain 10 ng/L (SDL) level. From this single standard addition point, the semi-quantitative concentration of compounds in the non-spiked sample can be calculated and a decision on possible confirmation (>10 ng/L) can be made.

For the confirmatory purpose, a six-point matrix-matched calibration curve was prepared. Blank ground- or surface water samples were spiked with the native working standard solution and the internal standard solution at the beginning of sample preparation. The calibration levels were as follows: 5, 50, 100, 250 and 500 ng/L. IS concentration was 100 ng/L. Samples were prepared as written in Section 4.3. IS evaluation was only used for those compounds that possessed the corresponding isotopically labelled analogue (Section 4.1). Other compounds were evaluated using external calibration method with matrix-matched calibration. The concentration of analytes could be directly obtained from the equation of linear calibrations weighted with the factor of 1/×. In this concentration range (5–500 ng/L), for each compound and in both sample types, determination coefficients obtained under the validation study were not lower than 0.9950.

### 4.6. Validation

In the case of screening validation, 10 blank groundwater and 10 blank surface water samples were prepared and analyzed with and without spiking. In total, 40 samples were analyzed: 20 blank and 20 spiked ones. One-point spike level was set at 10 ng/L as SDL concentration. The evaluation was based on the distinction of blanks and fortified samples at the SDL level, so appropriate quantification was not necessary.

In the case of confirmatory validation, ground- and surface water samples were validated separately. Two spiking levels were set as 10 (SDL level, RL) and 100 ng/L (regulatory level). Five samples were prepared at both levels and measurements were repeated on another day. In total, 10 samples were carried out at each level over two days for both ground- and surface water samples. Recovery and within-laboratory precision was calculated at each level for the 330 compounds in both ground- and surface water samples. The SDL/RL was set as the lowest validated concentration level, where the SNR was found to be higher than 10.

### 4.7. Automated SPE Enrichment

The same procedure was carried out as written in Section 4.3 using a higher volume of filtrated samples (500 mL). Samples were spiked with IS mixture (100 μL, 100 ng/mL) and one bag (6.5 g) QuEChERS salt mix was dissolved in each sample and 50 μL 10% (m/m) Na_2_-EDTA solution was added. The automated SPE steps were the following: as the first step, the system conditioned the cartridges (Strata-XL, 200 mg/6 mL, 100 μm) with 6 mL methanol, followed by 6 mL water. In the next step, the system passed the water samples (500 mL) through the cartridges. In the following step, the rinse of cartridges was carried out with 6 mL water. The flow rate was 1 mL/min in each step. Before sample elution, a drying step was performed by air for 5 min with a flow rate of 5 mL/min. As the last step, the target compounds were eluted from the cartridges into glass tubes with 6 mL methanol, followed by a drying step with air for 1 min with a flow rate of 5 mL/min. Then, samples were manipulated as written in Section 4.3. In this case, the dilution factor was 0.001 (1000-fold pre-concentration.)

Eight samples (groundwater) were extracted with automated SPE: one blank, six spikes (10 ng/L) and a single calibration point (100 ng/L). The recovery (%) and precision (%) were evaluated from the one-point matrix-matched calibration (standard addition).

## Figures and Tables

**Table 1:** Proficiency test results.

Compound	Sample	Result (ng/L)	Deviation from the Assigned Value%	Permitted Deviation%	Evaluation
Atrazine	1	51.2	−5	±35	Satisfactory
Atrazine	2	99.6	+3	±35	Satisfactory
Desethyl atrazine	1	51.7	+5	±35	Satisfactory
Desethyl atrazine	2	63.0	−1	±35	Satisfactory
Desizopropyl atrazine	1	54.6	+20	±35	Satisfactory
Metazachlor	1	27.0	−30	±35	Satisfactory
Metazachlor	2	74.0	−21	±35	Satisfactory
Metolachlor	1	55.0	+1	±35	Satisfactory
Metolachlor	2	183	+8	±35	Satisfactory
Simazine	1	50.0	−8	±35	Satisfactory
Simazine	2	53.0	−5	±35	Satisfactory

**Table 2:** Results of real sample analysis: Identified compounds under and above 10 ng/L and the total concentration. The detailed results are in Table S4.

Sample Type	Number of Compounds Identified under 10 ng/L (Screening)	Confirmed Concentration of Compounds under 10 ng/L	Identified Compound above 10 ng/L (Screening)	Confirmed Concentration Range and Sum of Compounds above 10 ng/L	Total Number of Confirmed Compounds	Sum Concentration of All Compounds (ng/L)
Groundwater #1	3	1.50–7.55	-	-	3	11.8
Surface water #1	2	2.25–4.33	Carbendazim, Thiabendazole	12.62–15.35 (∑28.0 ng/L)	4	34.5
Surface water #2	4	1.79–8.46	-	-	4	19.5
Surface water #3	11	1.69–9.52	Acetamiprid, Boscalid, Carbendazim, Fenhexamid, Imidacloprid, Penconazole, Terbuthylazine-desethyl	12.2–36.3 (∑163 ng/L)	18	213
Surface water #4	14	1.93–5.61	Azoxystrobin, Imidacloprid, Metolachlor, Thiabendazole	11.2–42.4 (∑92.9 ng/L)	18	159
Surface water #5	4	1.91–7.87	-	-	4	15.7
Surface water #6	9	1.07–9.28	-	-	9	30.0
Surface water #7	6	1.42–4.63	Carbendazim, Thiabendazole	14.1–16.4 (∑30.5 ng/L)	8	45.8
Surface water #8	5	1.30–3.75	Carbendazim, Thiabendazole	10.3–10.4 (∑20.7 ng/L)	7	31.3
Surface water #9	11	1.95–8.27	Atrazine-desethyl, Imidacloprid, Thiabendazole	10.9–43.9 (∑85.8 ng/L)	14	131
Surface water #10	6	1.00–4.29	Carbendazim, Thiabendazole	12.0–16.8 (∑28.8 ng/L)	8	45.2
Surface water #11	14	1.31–9.39	Azoxystrobin, Boscalid, Carbendazim, Imidacloprid, Isoproturon, Metolachlor, Tebuconazole, Thiabendazole	10.5–37.1 (∑146 ng/L)	8	219
Surface water #12	6	1.21–3.58	Carbendazim, Thiabendazole	10.1–10.1 (∑20.2 ng/L)	8	34.7
Surface water #13	6	1.00–4.37	Carbendazim, Thiabendazole	15.0–16.3 (∑31.3 ng/L)	8	46.1
Surface water #14	8	1.24–7.45	Atrazine-desethyl	31.9 (∑31.3 ng/L)	9	58.4
Surface water #15	5	1.07–9.23	Carbendazim	10.3 (∑10.3 ng/L)	6	26.2
Surface water #16	7	1.10–4.58	Carbendazim, Thiabendazole	10.6–12.0 (∑22.6 ng/L)	9	36.5
Surface water #17	4	1.16–3.45	Carbendazim, Thiabendazole	10.2–12.3 (∑22.5 ng/L)	6	30.8
Surface water #18	129	1.04–4.94	Carbendazim, Thiabendazole	18.8–14.4 (∑33.2 ng/L)	131	245
Surface water #19	8	1.07–4.18	Carbendazim, Thiabendazole	11.3–13.8 (∑25.0 ng/L)	10	41.5

**Table 3:** Examples for methods using only LC-MS for pesticide analysis in water.

Number of Compounds Analyzed	Sample Preparation	LOQ (ng/L)	Ref.
300	direct injection	100–1000	[14]
20	direct injection	0.5–2.0	[15]
102	direct injection	10–700	[16]
7	SPE	10	[18]
18	SPE	4–100	[19]
43	SPE	100–1000	[20]
150	SPE	100–1000	[21]
12	SPE	5–99	[29]
65	SPE	1.67–5.37	[30]
6	on-line SPE	1–15	[31]
22	SPE	2–1000	[32]

## Supplementary Materials

The following supporting information can be downloaded at: https://www.mdpi.com/article/10.3390/molecules27061872/s1, Figure S1: Extracted ion chromatogram of 480 pesticides in neat solvent at 100 ng/mL; Figure S2: Recovery (%) versus retention time (min) at 100 ng/L in groundwater; Figure S3: Precision (%) versus retention time (min) at 100 ng/L in groundwater; Figure S4: Recovery (%) versus retention time (min) at 100 ng/L in surface water; Figure S5: Precision (%) versus retention time (min) at 100 ng/L in surface water; Table S1: The scheduled MRM ion transitions of the tested 480 pesticides; Table S2: (a): Validation data for 330 pesticides in groundwater; (b): Validation data for 330 pesticides in surface water; Table S3: Recovery% and precision (RSD%) in groundwater at 10 ng/L spiking level using automated SPE enrichment; Table S4: Results of real sample analysis.

## Sample Availability

Samples of the compounds are not available from the authors.
