# Supplementary material for: An Alternative Strategy for Screening and Confirmation of 330 Pesticides in Ground- and Surface Water Using Liquid Chromatography Tandem Mass Spectrometry
Source: Molecules. 2022 Mar 14;27(6):1872. doi: 10.3390/molecules27061872 (PMC8950376; doi:10.3390/molecules27061872)
Supplement: Supplementary file 1 [file molecules-27-01872-s001.zip › molecules-1627265-supplementary.pdf]

An Alternative Strategy for Screening and Confirmation of 330 Pesticides in Ground- and Surface Water Using Liquid Chromatography Tandem Mass Spectrometry

Edgár Tóth [1,†], Ádám Tölgyesi [1,*,†], Andrea Simon [1], Mária Bálint [1], Xingmao Ma [2] and Virender K. Sharma [3,*]

[1] Bálint Analitika Ltd., Fehérvári út 144, 1116 Budapest, Hungary; ttedgar90@gmail.com (E.T.); schimocza@gmail.com (A.S.); hplc@balintanalitika.hu (M.B.)
[2] Department of Civil and Environmental Engineering, Texas A&M University, College Station, TX 77843, USA; samuelma@tamu.edu
[3] Program for the Environment and Sustainability, Department of Environmental and Occupational Health, School of Public Health, Texas A&M University, 212 Adriance Lab Road, 1266 TAMU, College Station, TX 77843, USA
* Correspondence: tolgyesi83@gmail.com (Á.T.); vsharma@tamu.edu (V.K.S.)
† These authors contributed equally to this work.

Citation: Tóth, E.; Tölgyesi, Á.; Simon, A.; Bálint, M.; Ma, X.; Sharma, V.K. An Alternative Strategy for Screening and Confirmation of 330 Pesticides in Ground- and Surface Water Using Liquid Chromatography Tandem Mass Spectrometry. *Molecules* **2022**, *27*, 1872. https://doi.org/10.3390/molecules27061872

Academic Editors: Jean-Christophe Garrigues and Florence Benoit-Marquié

Received: 21 February 2022
Accepted: 11 March 2022
Published: 14 March 2022

Publisher's Note: MDPI stays neutral with regard to jurisdictional claims in published maps and institutional affiliations.

Copyright: © 2022 by the authors. Submitted for possible open access publication under the terms and conditions of the Creative Commons Attribution (CC BY) license (https://creativecommons.org/licenses/by/4.0/).

Figure S1: Extracted ion chromatogram of 480 pesticides in neat solvent at 100 ng/mL.

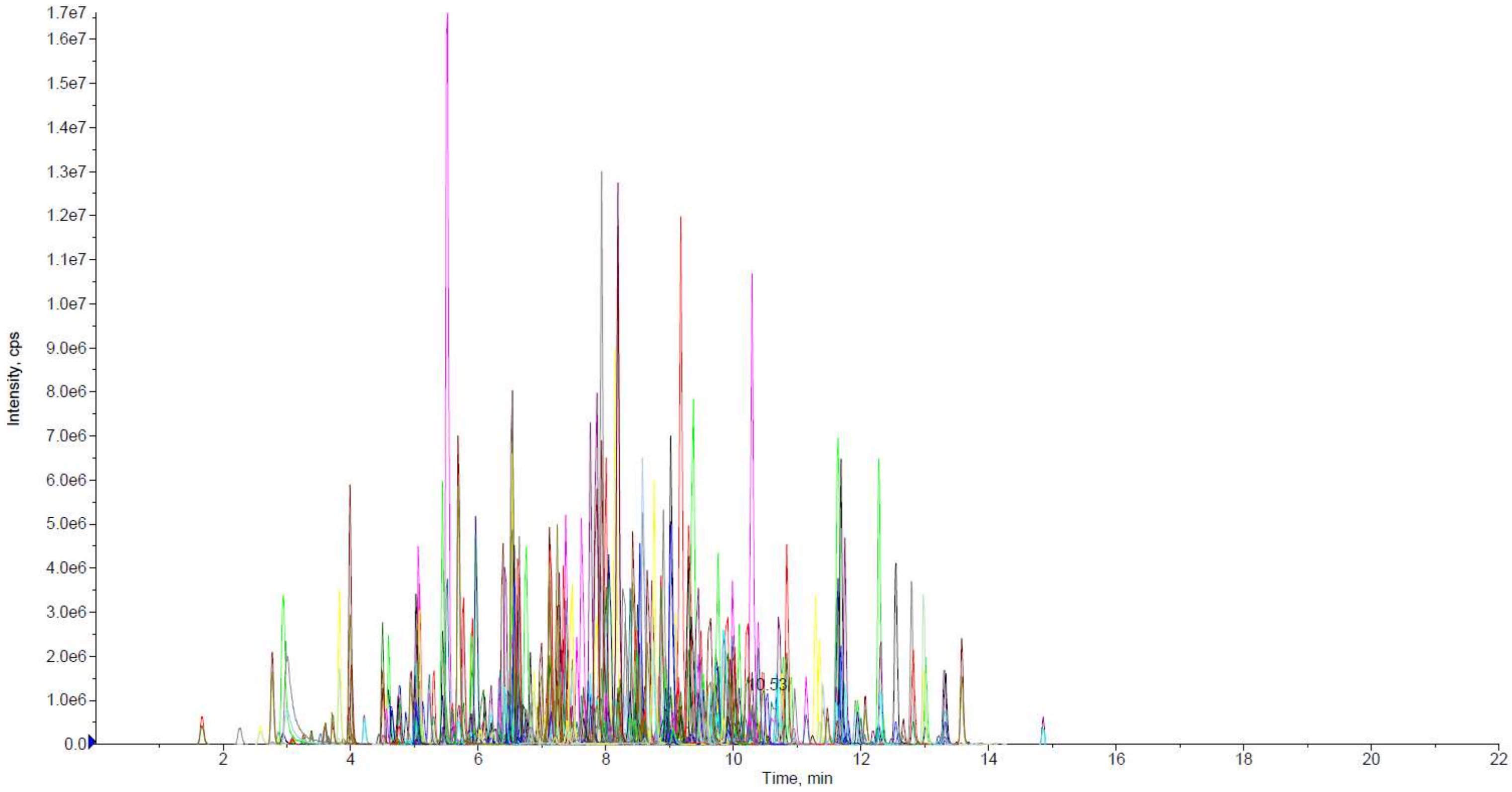

Figure S2. Recovery (%) *versus* retention time (min) at 100 ng/L in groundwater.

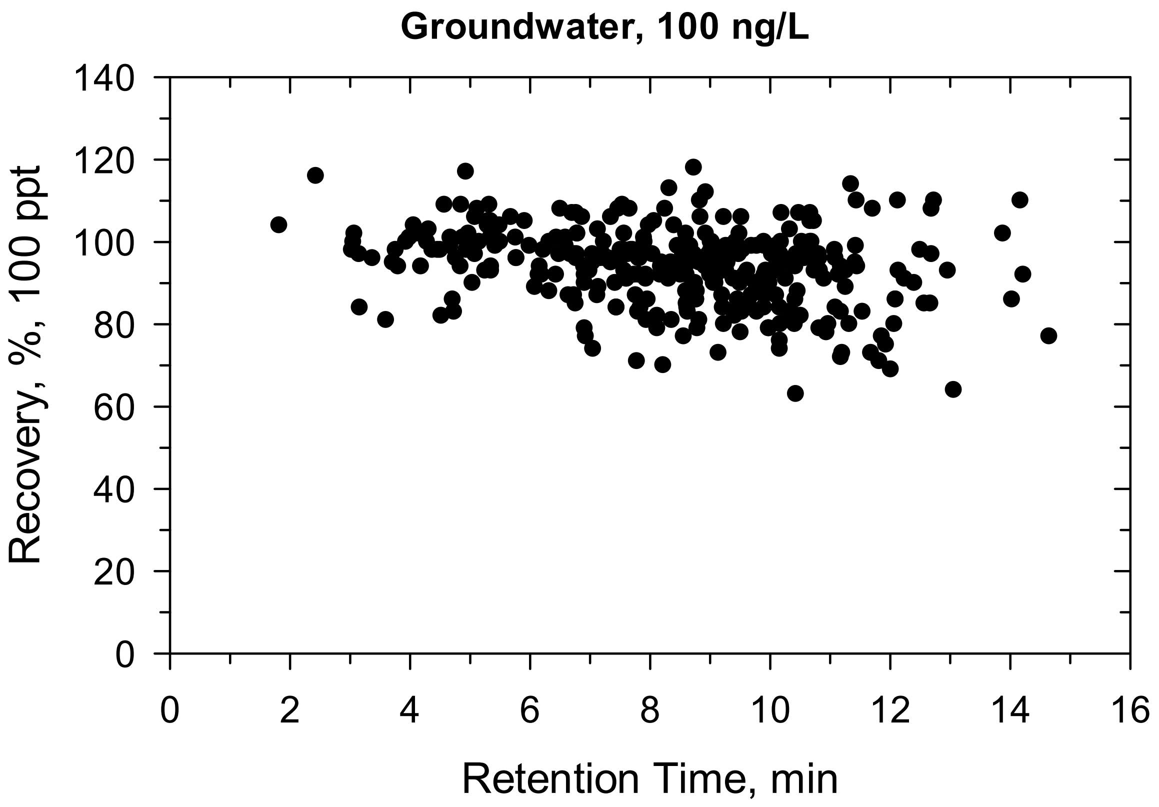

Figure S3. Precision (%) *versus* retention time (min) at 100 ng/L in groundwater.

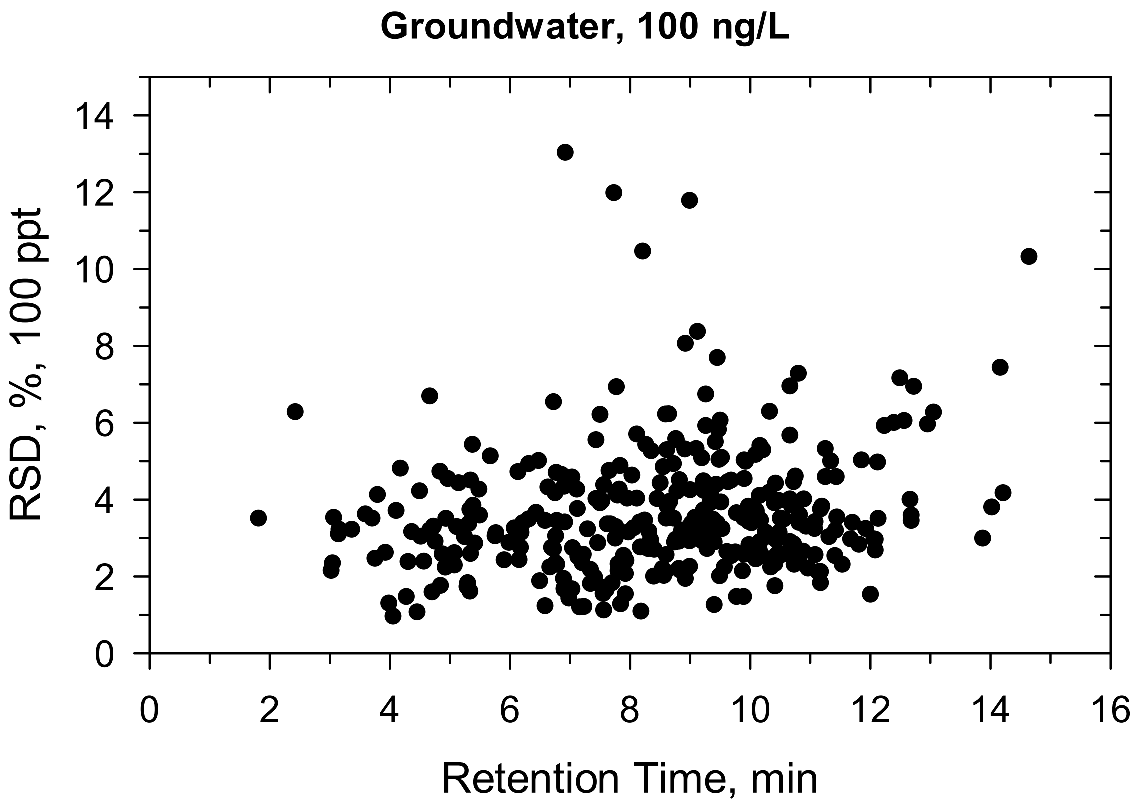

Figure S4. Recovery (%) *versus* retention time (min) at 100 ng/L in surface water.

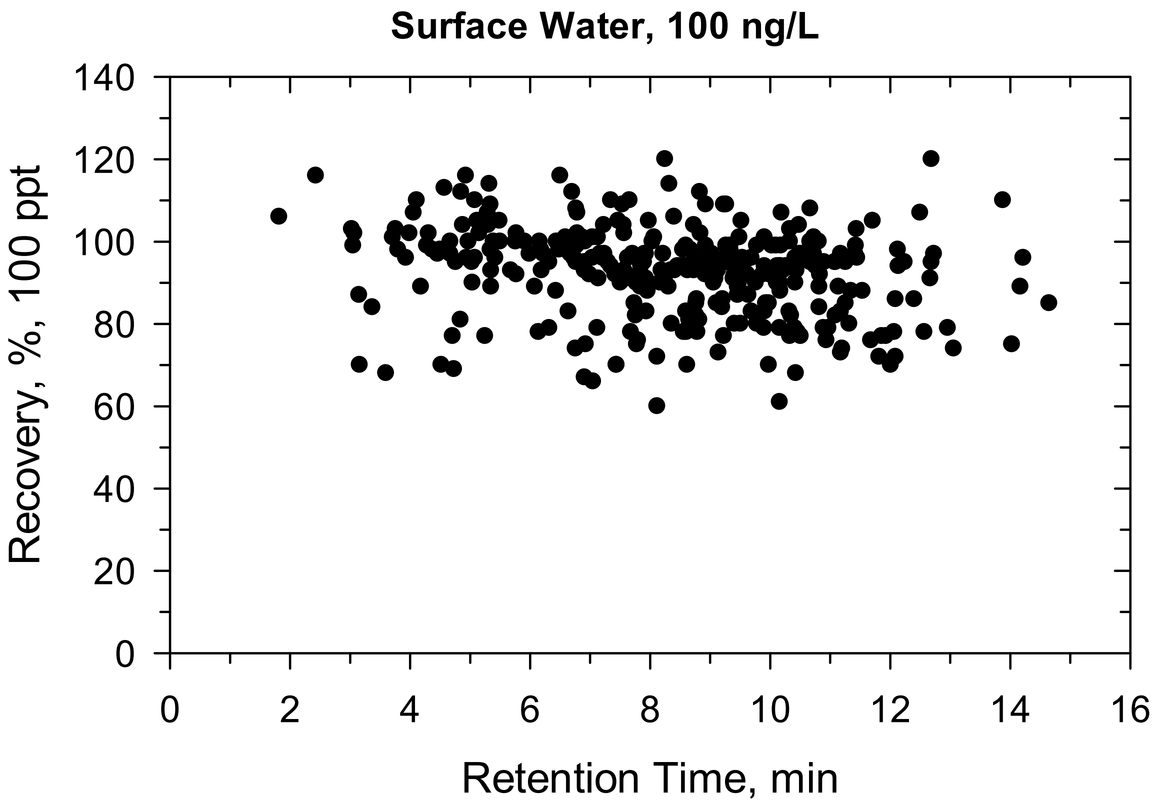

Figure S5. Precision (%) *versus* retention time (min) at 100 ng/L in surface water.

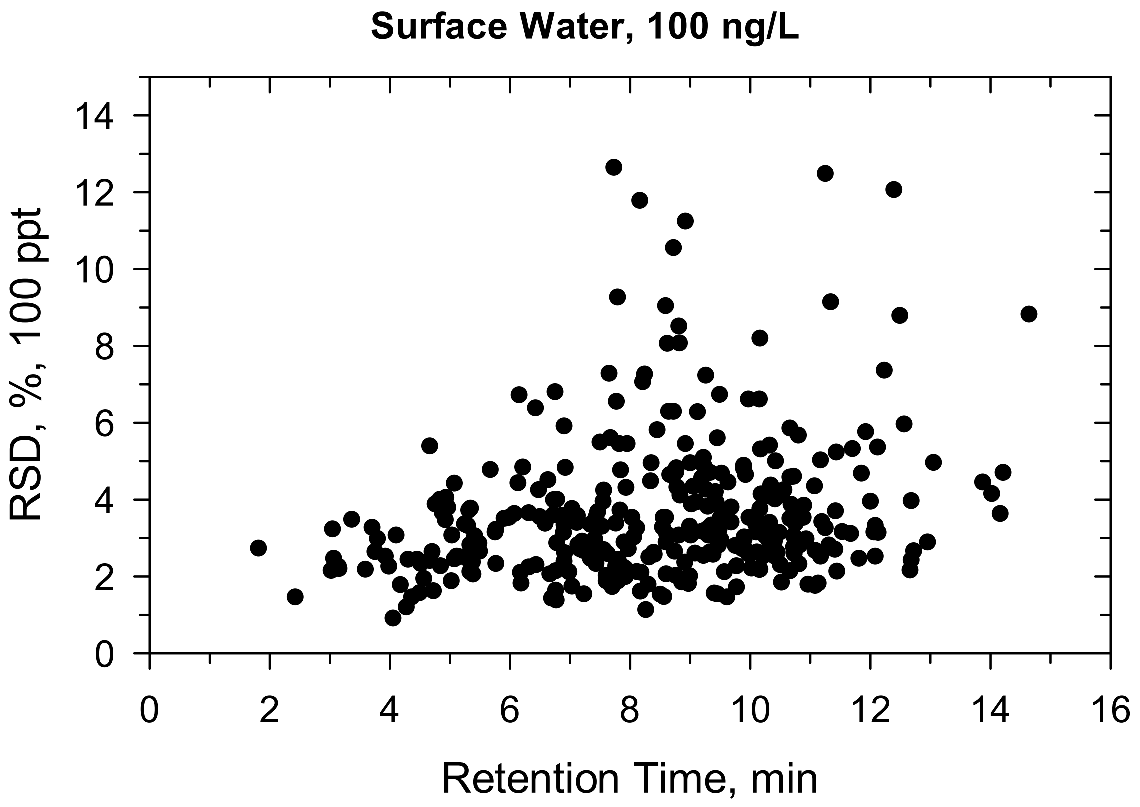

Table S1: The scheduled MRM ion transitions of the tested 480 pesticides, the MRM time window was 120 s, the cycle was 0.400 s. First ion transition was used for quantification and the second was used for confirmation. Those compounds are highlighted that did not fulfill the recovery criteria of 60-120%. CE: collision energy; CXP: collision cell exit potential; DP: declustering potential; EP: entrance potential; IS: internal standard; Rt: retention time.

Compound	Rt (min)	Precursor ion (m/z)	Product ion (m/z)	DP (V)	EP (V)	CE (V)	CXP (V)	IS
3-Hydroxycarbofuran.1	5.3	238.1	181.0	66	10	15	12	
3-Hydroxycarbofuran.2	5.3	238.1	163.0	66	10	19	10	
Acephate.1	2.4	184.1	143.0	46	10	11	28	
Acephate.2	2.4	184.1	49.0	46	10	35	0	
Acetamiprid.1	5.3	223.0	126.0	68	10	29	8	Acetamiprid-d3.1
Acetamiprid.2	5.3	223.0	99.0	68	10	53	6	Acetamiprid-d3.1
Acetochlor.1	9.5	270.3	224.1	111	10	15	12	
Acetochlor.2	9.5	270.3	148.0	111	10	25	16	
Acibenzolar-S-methyl.1	8.5	211.0	136.2	70	10	41	8	
Acibenzolar-S-methyl.2	8.5	211.0	91.2	70	10	29	4	
Aclonifen.1	9.9	265.0	248.2	76	10	27	12	
Aclonifen.2	9.9	265.0	182.2	76	10	39	12	
Acrinathrin.1	12.9	559.1	208.1	36	10	19	12	
Acrinathrin.2	12.9	559.1	181.0	36	10	33	10	
Alachlor.1	9.5	270.0	162.0	91	10	27	16	
Alachlor.2	9.5	270.0	147.0	91	10	47	18	
Aldicarb.1	6.0	208.1	116.0	26	10	11	6	
Aldicarb.2	6.0	208.1	89.0	26	10	23	4	
Aldicarb-sulfone.1	3.6	240.1	148.2	36	10	25	6	
Aldicarb-sulfone.2	3.6	240.1	86.2	36	10	29	12	
Aldicarb-sulfoxide.1	3.3	207.1	132.1	56	10	9	8	
Aldicarb-sulfoxide.2	3.3	207.1	89.1	56	10	19	6	

Ametoctradin.1	10.9	276.0	177.0	186	10	45	18	
Ametoctradin.2	10.9	276.0	149.0	181	10	47	16	
Ametryn.1	8.4	228.1	186.1	65	10	25	12	
Ametryn.2	8.4	228.1	96.0	65	10	35	6	
Amidosulfuron.1	4.5	370.0	261.1	65	10	20	12	
Amidosulfuron.2	4.5	370.0	218.1	65	10	35	12	
Aminocarb.1	6.8	209.1	137.1	56	10	33	8	
Aminocarb.2	6.8	209.1	152.0	56	10	19	10	
Amitraz.1	10.2	294.2	148.3	98	10	22	9	
Amitraz.2	10.2	294.2	91.2	98	10	57	5	
Anilofos.1	10.1	368.0	199.0	65	10	20	12	
Anilofos.2	10.1	368.0	125.0	65	10	50	12	
Atrazine.1	7.5	215.9	174.0	56	10	25	15	Atrazine-d5.1
Atrazine.2	7.5	215.9	104.1	56	10	39	15	Atrazine-d5.1
Atrazine-desethyl.1	5.7	188.1	145.9	1	10	23	16	
Atrazine-desethyl.2	5.7	188.1	43.0	1	10	59	20	
Atrazine-desisopropyl.1	4.7	174.0	104.0	51	10	31	14	Atrazine-desisopropyl-d5.1
Atrazine-desisopropyl.2	4.7	174.0	67.9	1	10	55	30	Atrazine-desisopropyl-d5.1
Azaconazole.1	7.7	300.0	159.1	65	10	35	12	
Azaconazole.2	7.7	300.0	231.1	65	10	35	12	
Azamethiphos.1	6.6	325.0	183.0	65	10	31	12	
Azamethiphos.2	6.6	325.0	112.0	65	10	39	12	
Azinphos-ethyl.1	9.2	346.0	132.1	50	10	31	15	
Azinphos-ethyl.2	9.2	346.0	137.0	50	10	32	15	
Azinphos-methyl.1	8.0	318.0	132.2	56	10	23	12	
Azinphos-methyl.2	8.0	318.0	160.2	56	10	13	12	
Aziprotryne.1	8.8	226.0	156.0	65	10	25	12	

Aziprotryne.2	8.8	226.0	125.0	65	10	27	12	
Azoxystrobin.1	8.2	404.1	372.1	65	10	19	10	
Azoxystrobin.2	8.2	404.1	344.1	65	10	33	10	
Benalaxyl.1	10.2	326.2	148.1	60	10	29	8	
Benalaxyl.2	10.2	326.2	294.1	60	10	15	8	
Bendiocarb.1	6.8	224.1	167.1	61	10	15	10	
Bendiocarb.2	6.8	224.1	109.0	61	10	33	6	
Benflutamid.1	9.9	356.0	91.0	111	10	77	10	
Benflutamid.2	9.9	356.0	162.0	111	10	33	20	
Bensulfuron-metil.1	7.2	411.1	149.1	65	10	35	12	
Bensulfuron-metil.2	7.2	411.1	182.1	65	10	26	12	
Benthiavalicarb-isopropyl.1	8.8	382.2	197.0	65	10	29	12	
Benthiavalicarb-isopropyl.2	8.8	382.2	116.0	65	10	37	12	
Benzolyprop-ethyl.1	10.3	366.0	105.0	66	10	17	12	
Benzolyprop-ethyl.2	10.3	366.0	320.0	66	10	13	20	
Benzoximate.1	10.6	364.0	199.0	31	10	11	12	
Benzoximate.2	10.6	364.0	105.0	31	10	31	6	
Benzthiazuron.1	8.6	208.0	151.0	65	10	16	12	
Benzthiazuron.2	8.6	208.0	109.0	65	10	16	12	
Bifenazate.1	9.1	301.1	198.1	66	10	13	12	
Bifenazate.2	9.1	301.1	170.1	66	10	27	10	
Bitertanol.1	10.6	338.2	269.2	56	10	13	2	
Bitertanol.2	10.6	338.2	70.0	56	10	29	12	
Boscalid.1	8.6	343.0	140.0	35	10	25	8	Boscalid-d4.1
Boscalid.2	8.6	343.0	307.0	35	10	27	8	Boscalid-d4.1
Bromacil.1	6.7	261.0	205.0	65	10	35	12	
Bromacil.2	6.7	261.0	188.0	65	10	42	12	
Bromucanozole-Isomer.1.1	9.0	378.0	159.0	46	10	37	2	

Bromucanozole-Isomer.1.2	9.0	378.0	70.0	46	10	47	6	
Bromucanozole-Isomer.2.1	9.8	378.0	159.0	46	10	37	2	
Bromucanozole-Isomer.2.2	9.8	378.0	70.0	46	10	47	6	
Bupirimate.1	9.7	317.0	166.1	91	10	33	10	
Bupirimate.2	9.7	317.0	108.0	91	10	35	6	
Buprofezin.1	11.8	306.2	201.1	56	10	17	12	
Buprofezin.2	11.8	306.2	116.2	36	10	23	6	
Butachlor.1	11.9	312.2	238.2	65	10	21	12	
Butachlor.2	11.9	312.2	57.3	65	10	46	12	
Butafenacil.1	9.2	492.1	331.0	68	10	27	8	
Butafenacil.2	9.2	492.1	349.0	68	10	19	10	
Butocarboxim.1	6.1	213.1	75.1	86	10	19	3	
Butocarboxim.2	6.1	213.1	116.0	86	10	17	6	
Butocarboxim-sulfoxide.1	3.1	207.0	74.9	56	10	19	12	
Butocarboxim-sulfoxide.2	3.1	207.0	90.0	56	10	19	12	
Butoxycarboxim.1	3.5	223.1	106.0	61	10	13	6	
Butoxycarboxim.2	3.5	223.1	166.0	61	10	11	10	
Buturon.1	7.6	237.1	126.1	65	10	50	12	
Buturon.2	7.6	237.1	99.0	65	10	50	12	
Cadusafos.1	10.5	271.0	158.9	65	10	20	12	
Cadusafos.2	10.5	271.1	130.9	65	10	38	12	
Carbaryl.1	7.0	202.1	145.0	51	10	13	8	Carbaryl-d7.1
Carbaryl.2	7.0	202.1	127.0	51	10	39	32	Carbaryl-d7.1
Carbendazim.1	5.8	192.2	160.2	61	10	25	10	Carbendazim-d3.1
Carbendazim.2	5.8	192.2	132.1	61	10	41	8	Carbendazim-d3.1
Carbetamide.1	6.4	237.1	192.0	56	10	13	12	
Carbetamide.2	6.4	237.1	118.1	56	10	17	6	
Carbofuran.1	6.8	222.1	165.1	65	10	17	10	

Carbofuran.2	6.8	222.1	123.0	65	10	29	8	
Carboxin.1	7.0	236.1	143.0	66	10	21	8	
Carboxin.2	7.0	236.1	87.0	66	10	33	6	
Carfentrazon.1	10.0	429.0	366.0	106	10	31	18	
Carfentrazon.2	10.0	429.0	345.9	106	10	37	18	
Carfentrazone-ethyl.1	9.9	412.0	346.0	91	10	31	6	
Carfentrazone-ethyl.2	9.9	412.0	366.0	91	10	25	10	
Chlorantraniliprole.1	7.9	484.0	452.9	61	10	21	12	
Chlorantraniliprole.2	7.9	484.0	285.9	61	10	17	8	
Chlorbromuron.1	8.5	293.0	204.0	76	10	29	12	
Chlorbromuron.2	8.5	293.0	182.0	76	10	25	12	
Chlorbufam NH4.1	8.3	241.0	224.0	1	10	9	20	
Chlorbufam NH4.2	8.3	241.0	171.9	1	10	17	8	
Chlorbufam.1	8.3	224.1	171.9	56	10	13	12	
Chlorbufam.2	8.3	224.1	153.9	56	10	25	12	
Chlorbufam.3	8.3	224.0	172.0	1	10	11	10	
Chlorbufam.4	8.3	224.0	153.9	1	10	23	18	
Chlorfenvinphos.1	10.3	359.0	155.0	61	10	19	12	
Chlorfenvinphos.2	10.3	358.9	99.0	61	10	50	12	
Chlorfluazuron.1	12.8	540.0	383.0	93	10	27	10	
Chlorfluazuron.2	12.8	540.0	158.0	93	10	29	10	
Chloridazon.1	5.4	222.0	51.0	111	10	50	24	
Chloridazon.2	5.4	222.0	77.0	111	10	55	10	
Chlorotoluron.1	7.3	213.1	72.2	68	10	31	4	Chlorotoluron-d6.1
Chlorotoluron.2	7.3	213.1	46.2	68	10	35	8	Chlorotoluron-d6.1
Chloroxuron.1	9.0	291.1	72.4	81	10	47	4	
Chloroxuron.2	9.0	291.1	218.1	81	10	33	14	
Chlorpyrifos.1	12.0	349.8	197.8	56	10	25	54	

Chlorpyrifos.2	12.0	349.8	96.9	56	10	69	12	
Chlorpyrifos-methyl.1	10.7	321.9	124.9	81	10	29	12	
Chlorpyrifos-methyl.2	10.7	321.9	289.9	81	10	21	12	
Chlorsulfuron.1	5.1	358.1	167.2	65	10	35	12	
Chlorsulfuron.2	5.1	358.1	141.1	65	10	50	12	
Chlorthiophos.1	12.5	360.9	305.0	56	10	20	12	
Chlorthiophos.2	12.5	360.9	192.0	56	10	40	12	
Chromafenozide.1	9.1	395.1	175.0	65	10	22	12	
Chromafenozide.2	9.1	395.1	339.0	65	10	10	12	
Cinosulfuron.1	5.1	414.1	183.2	65	10	29	12	
Cinosulfuron.2	5.1	414.1	157.1	65	10	35	12	
Cirimidine.1	6.1	172.0	136.0	61	10	27	16	
Cirimidine.2	6.1	172.0	107.0	56	10	41	12	
Clethodim-Isomer.1.1	7.4	360.1	164.0	51	10	29	10	
Clethodim-Isomer.1.2	7.4	360.1	268.1	51	10	17	18	
Clethodim-Isomer.2.1	9.1	360.1	164.0	51	10	29	10	
Clethodim-Isomer.2.2	9.1	360.1	268.1	51	10	17	18	
Clofentezine.1	10.7	303.0	138.0	56	10	19	8	
Clofentezine.2	10.7	303.0	102.0	56	10	51	6	
Clomazone.1	8.1	240.1	125.0	66	10	27	12	
Clomazone.2	8.1	240.1	89.1	66	10	65	12	
Cloquintocet-mexyl.1	11.8	336.2	238.0	65	10	23	12	
Cloquintocet-mexyl.2	11.8	336.2	192.1	65	10	38	12	
Clothianidin.1	4.9	250.0	169.0	56	10	19	10	
Clothianidin.2	4.9	250.0	132.0	56	10	21	8	
Coumaphos.1	10.2	363.0	227.0	60	10	35	10	
Coumaphos.2	10.2	363.0	211.1	60	10	53	10	
Crufomat(Ruelene).1	9.7	292.0	108.1	65	10	39	12	

Crufomat(Ruelene).2	9.7	292.0	236.1	65	10	18	12	
Cyanazine.1	6.5	241.1	214.1	65	10	35	12	
Cyanazine.2	6.5	241.1	104.0	65	10	45	12	
Cyanofenphos.1	10.0	304.0	275.9	121	10	17	32	
Cyanofenphos.2	10.0	304.0	156.9	121	10	29	16	
Cyanophos.1	7.9	261.0	125.0	65	10	35	12	
Cyanophos.2	7.9	261.0	78.9	65	10	50	12	
Cyantraniliprole.1	7.0	475.1	286.0	71	10	19	10	
Cyantranilipore.2	7.0	475.1	444.0	71	10	23	10	
Cyazofamid.1	9.4	325.2	108.0	50	10	18	6	
Cyazofamid.2	9.4	325.2	261.2	50	10	14	6	
Cybutrine.1	9.6	254.3	198.0	121	10	25	20	
Cybutrine.2	9.6	254.3	68.0	121	10	57	8	
Cyflufenamid.1	10.6	413.2	295.1	65	10	15	12	
Cyflufenamid.2	10.6	413.2	203.0	65	10	32	12	
Cymiazole.1	9.0	219.0	144.0	1	10	49	20	
Cymiazole.2	9.0	219.0	171.0	101	10	37	22	
Cymoxanil.1	5.6	198.9	128.2	56	10	12	7	
Cymoxanil.2	5.6	198.9	111.2	56	10	23	6	
Cyproconazole-Isomer.1.1	8.8	292.0	70.0	66	10	31	2	
Cyproconazole-Isomer.1.2	8.8	292.0	125.0	66	10	29	10	
Cyproconazole-Isomer.2.1	9.1	292.0	70.0	66	10	31	2	
Cyproconazole-Isomer.2.2	9.1	292.0	125.0	66	10	29	10	
Cyprodinil.1	10.2	226.0	93.0	91	10	47	6	
Cyprodinil.2	10.2	226.0	77.0	91	10	61	4	
Cyromazine.1	2.9	167.1	85.1	89	10	26	4	Cyromazine-d4.1
Cyromazine.2	2.9	167.1	125.1	89	10	24	7	Cyromazine-d4.1
Demeton-S.1	8.1	259.0	89.0	65	10	45	12	

Demeton-S.2	8.1	259.0	61.1	65	10	52	12	
Demeton-S-methyl NH4.1	7.5	230.9	61.0	46	10	47	12	
Demeton-S-methyl NH4.2	7.5	230.9	89.1	46	10	17	12	
Demeton-S-methylsulphone.1	4.2	263.0	168.9	61	10	23	14	
Demeton-S-methylsulphone.2	4.2	262.9	108.9	106	10	37	10	
Demeton-S-sulfone.1	5.7	291.0	121.0	61	10	23	14	
Demeton-S-sulfone.2	5.7	291.0	197.0	61	10	23	10	
Demeton-S-sulfoxide.1	5.6	275.0	197.0	61	10	23	10	
Demeton-S-sulfoxide.2	5.6	275.0	105.0	61	10	23	14	
Desmedipham.1	7.8	318.1	182.0	36	10	19	12	
Desmedipham.2	7.8	318.1	154.0	36	10	35	26	
Desmetryn.1	7.5	214.1	172.2	65	10	35	12	
Desmetryn.2	7.5	214.1	82.0	65	10	50	12	
Diafentiouron.1	12.6	385.2	328.8	116	10	31	14	
Diafentiouron.2	12.6	385.2	286.8	116	10	37	14	
Diallát.1	11.3	270.0	109.0	65	10	50	12	
Diallát.2	11.3	270.0	228.0	65	10	20	12	
Diazinon.1	10.3	305.1	169.1	61	10	29	14	Diazinon-d10.1
Diazinon.2	10.3	305.1	97.0	61	10	41	14	Diazinon-d10.1
Dichlorvos.1	6.7	220.8	109.1	60	10	35	14	Dichlorvos-d6.1
Dichlorvos.2	6.7	220.8	94.9	60	10	50	14	Dichlorvos-d6.1
Diclobutrazol.1	9.9	328.2	70.2	70	10	48	3	
Diclobutrazol.2	9.9	328.2	59.1	70	10	48	2	
Dicrotophos.1	4.8	238.1	193.0	61	10	13	12	
Dicrotophos.2	4.8	238.1	112.1	61	10	17	6	
Diethofencarb.1	8.3	268.1	226.1	61	10	13	14	
Diethofencarb.2	8.3	268.1	124.0	61	10	43	8	
Difenoconazole-Isomer.1.1	10.8	408.2	253.1	46	10	31	6	Difenoconazole-d4.1

Difenoconazole-Isomer.1.2	10.8	406.1	251.1	46	10	41	2	Difenoconazole-d4.1
Difenoxuron.1	7.6	287.0	72.0	96	10	47	12	
Difenoxuron.2	7.6	287.0	123.0	96	10	29	12	
Diflubenzuron.1	9.6	311.0	158.2	61	10	19	10	
Diflubenzuron.2	9.6	311.0	141.1	61	10	43	8	
Diflufenican.1	11.1	395.0	266.0	65	10	35	12	Diflufenican-d3.1
Diflufenican.2	11.1	395.0	246.0	65	10	23	12	Diflufenican-d3.1
Dimefox.1	4.8	155.1	110.0	71	10	25	12	
Dimefox.2	4.8	155.1	44.0	71	10	39	20	
Dimefuron.1	8.0	339.0	72.0	136	10	41	12	
Dimefuron.2	8.0	339.0	167.0	136	10	31	12	
Dimethachlor.1	7.9	256.0	224.0	71	10	17	26	
Dimethachlor.2	7.9	256.0	148.1	71	10	33	14	
Dimethenamid.1	8.5	275.9	244.0	71	10	19	26	
Dimethenamid.2	8.5	275.9	168.1	71	10	31	18	
Dimethirimol.1	7.4	210.1	71.1	65	10	47	12	
Dimethirimol.2	7.4	210.1	140.0	65	10	38	12	
Dimethoate.1	5.3	230.0	199.0	46	10	13	12	Dimethoate-d6.1
Dimethoate.2	5.3	230.0	125.0	46	10	29	8	Dimethoate-d6.1
Dimethomorph-Isomer.1.1	8.3	388.1	301.0	75	10	29	0	
Dimethomorph-Isomer.1.2	8.3	388.1	165.1	75	10	45	10	
Dimethomorph-Isomer.2.1	8.7	388.1	301.0	75	10	29	0	
Dimethomorph-Isomer.2.2	8.7	388.1	165.1	75	10	45	10	
Dimoxystrobin.1	9.8	327.1	116.0	66	10	29	6	
Dimoxystrobin.2	9.8	327.1	205.0	66	10	23	14	
Diniconazole.1	10.7	326.2	70.2	72	10	50	3	
Diniconazole.2	10.7	326.2	159.0	72	10	43	10	
Dinotefuran.1	3.3	203.1	157.2	51	10	17	10	

Dinotefuran.2	3.3	203.1	129.2	51	10	11	8	
Dioxacarb.1	5.3	224.1	167.0	51	10	11	10	
Dioxacarb.2	5.3	224.1	123.0	51	10	21	18	
Diphenamid.1	7.9	240.1	134.1	65	10	28	12	
Diphenamid.2	7.9	240.1	167.1	65	10	25	12	
Dipropetryn.1	10.1	256.0	214.0	121	10	27	12	
Dipropetryn.2	10.1	256.0	144.0	116	10	37	16	
Disulfoton NH4.1	10.8	292.0	275.1	56	10	5	20	
Disulfoton NH4.2	10.8	292.0	88.9	56	10	13	12	
Disulfoton.1	10.8	275.1	61.0	56	10	56	12	
Disulfoton.2	10.8	275.1	89.0	56	10	35	12	
Disulfoton.3	10.8	274.9	89.1	1	10	11	14	
Disulfoton.4	10.8	274.9	60.9	1	10	47	8	
Disulfoton-sulfone.1	7.4	307.1	171.0	60	10	27	12	
Disulfoton-sulfone.2	7.4	307.1	153.0	60	10	32	12	
Disulfoton-sulfoxide.1	7.3	291.1	212.9	60	10	22	12	
Disulfoton-sulfoxide.2	7.3	291.1	185.0	60	10	32	12	
Diuron.1	7.7	233.1	72.0	71	10	37	14	
Diuron.2	7.7	235.1	72.1	66	10	33	4	
DMST.1	6.9	215.2	78.9	65	10	50	12	
DMST.2	6.9	215.2	106.0	65	10	44	12	
Dodemorph.1	13.9	282.1	116.0	65	10	35	12	
Dodemorph.2	13.9	282.1	98.0	65	10	50	12	
Dodine.1	10.4	228.3	57.0	91	10	41	10	
Dodine.2	10.4	228.3	186.2	91	10	27	10	
Doramectin.1	13.9	916.6	331.4	61	10	33	8	
Doramectin.2	13.9	916.6	593.5	66	10	19	20	
Emamectin-benzoate b1a.1	12.4	886.5	158.1	106	10	49	10	

Emamectin-benzoate b1a.2	12.4	886.5	82.1	106	10	107	6	
Emamectin-benzoate b1b.1	12.1	872.6	158.2	101	10	51	10	
Emamectin-benzoate b1b.2	12.1	872.6	302.3	101	10	43	26	
Epoxiconazole.1	9.4	330.0	121.1	41	10	49	10	
Epoxiconazole.2	9.4	330.0	101.1	41	10	69	2	
Etaconazole-Isomer.1.1	9.4	328.1	159.0	91	10	31	12	
Etaconazole-Isomer.1.2	9.4	328.1	205.0	91	10	23	6	
Etaconazole-Isomer.2.1	9.8	328.1	159.0	91	10	31	12	
Etaconazole-Isomer.2.2	9.8	328.1	205.0	91	10	23	6	
Ethidimuron.1	5.0	265.0	208.1	65	10	14	12	
Ethidimuron.2	5.0	265.0	114.0	65	10	36	12	
Ethiofencarb.1	7.2	226.1	107.2	56	10	21	6	
Ethiofencarb.2	7.2	226.1	164.0	56	10	21	6	
Ethiofencarb-sulfone.1	4.8	275.0	107.1	56	10	29	12	
Ethiofencarb-sulfone.2	4.8	275.0	201.1	56	10	17	12	
Ethiofencarb-sulfoxide.1	4.9	242.0	107.0	71	10	13	12	
Ethiofencarb-sulfoxide.2	4.9	242.0	185.1	71	10	23	12	
Ethion.1	12.0	385.0	143.0	65	10	35	12	
Ethion.2	12.0	385.0	96.9	65	10	50	12	
Ethiprole.1	8.4	397.3	255.2	96	10	47	16	
Ethiprole.2	8.4	397.3	350.9	96	10	27	10	
Ethirimol.1	7.7	210.2	140.1	96	10	31	8	
Ethirimol.2	7.7	210.2	98.1	96	10	39	12	
Ethofumesate.1	8.3	287.1	259.1	76	10	15	6	
Ethofumesate.2	8.3	287.1	121.1	76	10	23	6	
Ethoprophos.1	9.4	243.0	131.0	66	10	32	12	
Ethoprophos.2	9.4	243.0	97.0	66	10	30	12	
Ethoxyquin.1	9.5	218.1	174.0	86	10	39	12	

Ethoxyquin.2	9.5	218.1	160.0	86	10	47	12	
Etofenprox NH4.1	13.9	394.0	177.0	51	10	21	14	
Etofenprox NH4.2	13.9	394.0	107.0	51	10	61	18	
Etoxazole.1	12.4	360.1	141.0	31	10	59	8	
Etoxazole.2	12.4	360.1	57.2	31	10	51	10	
Etrimfos.1	10.1	293.1	265.1	65	10	22	12	
Etrimfos.2	10.1	293.1	125.0	65	10	35	12	
Famoxadone.1	10.3	392.0	331.0	46	10	13	10	
Famoxadone.2	10.3	392.0	238.0	46	10	23	16	
Fenamidone.1	8.5	312.1	92.0	36	10	35	6	
Fenamidone.2	8.5	312.1	236.1	36	10	21	14	
Fenamiphos.1	9.7	304.0	217.0	81	10	37	18	
Fenamiphos.2	9.7	304.0	202.0	86	10	49	10	
Fenamiphos-sulfone.1	6.9	336.0	266.0	81	10	29	22	
Fenamiphos-sulfone.2	6.9	336.0	308.0	81	10	23	8	
Fenamiphos-sulfoxide.1	6.8	320.0	233.0	81	10	37	20	
Fenamiphos-sulfoxide.2	6.8	320.0	171.0	76	10	33	14	
Fenarimol.1	9.2	331.0	268.0	81	10	35	26	
Fenarimol.2	9.2	331.0	81.0	81	10	55	14	
Fenazaquin.1	13.2	307.1	161.1	76	10	27	10	
Fenazaquin.2	13.2	307.1	147.0	76	10	25	8	
Fenbuconazole.1	9.5	337.0	124.9	61	10	55	14	
Fenbuconazole.2	9.5	337.0	70.0	61	10	39	6	
Fenfuram.1	7.0	202.1	109.1	65	10	45	12	
Fenfuram.2	7.0	202.1	91.8	65	10	50	12	
Fenhexamid.1	9.2	302.0	97.0	95	10	33	6	
Fenhexamid.2	9.2	302.0	55.0	95	10	61	10	
Fenitrothion.1	8.9	278.1	169.1	60	10	30	12	

Fenitrothion.2	8.9	278.1	125.0	60	10	45	12	
Fenitrothion.3	8.9	277.9	124.9	41	10	25	14	
Fenitrothion.4	8.9	277.9	136.0	41	10	15	16	
Fenobucarb.1	8.2	208.2	95.3	61	10	19	6	
Fenobucarb.2	8.2	208.2	152.1	61	10	13	8	
Fenoxaprop-P-ethyl.1	11.5	362.1	288.0	65	10	32	12	
Fenoxaprop-P-ethyl.2	11.5	362.1	91.9	65	10	50	12	
Fenoxycarb.1	9.7	302.1	116.1	61	10	17	6	
Fenoxycarb.2	9.7	302.1	88.0	61	10	29	6	
Fenpropimorph.1	13.8	304.0	147.0	141	10	39	12	
Fenpropimorph.2	13.8	304.0	117.0	141	10	73	2	
Fenpyroximate.1	12.7	422.0	366.1	88	10	23	10	
Fenpyroximate.2	12.7	422.0	135.1	88	10	53	8	
Fensulfothion.1	7.6	309.1	280.8	65	10	18	12	
Fensulfothion.2	7.6	309.1	252.9	65	10	22	12	
Fensulfothion-oxon.1	6.0	293.0	237.0	65	10	19	12	
Fensulfothion-oxon.2	6.0	293.0	265.0	65	10	14	12	
Fensulfothion-oxon-sulfone.1	6.2	309.0	253.0	65	10	16	12	
Fensulfothion-oxon-sulfone.2	6.2	309.0	175.0	65	10	27	12	
Fensulfothion-sulfone.1	7.7	325.1	268.9	65	10	17	12	
Fensulfothion-sulfone.2	7.7	325.1	297.0	65	10	20	12	
Fenthion.1	10.1	279.1	169.1	65	10	35	12	
Fenthion.2	10.1	279.1	105.1	65	10	50	12	
Fenthion-oxon-sulfone.1	5.6	295.1	217.2	120	10	15	10	
Fenthion-oxon-sulfone.2	5.6	295.1	78.1	120	10	40	10	
Fenthion-oxon-sulfoxide.1	5.4	279.1	264.2	130	10	15	10	
Fenthion-oxon-sulfoxide.2	5.4	279.1	104.2	130	10	25	10	
Fenthion-sulfone.1	7.0	311.1	125.0	65	10	28	12	

Fenthion-sulfone.2	7.0	311.0	109.0	65	10	39	12	
Fenthion-sulfoxid.1	6.9	295.1	279.7	76	10	25	16	
Fenthion-sulfoxid.2	6.9	295.1	108.9	76	10	45	18	
Fenuron.1	5.1	165.1	72.1	55	10	45	4	
Fenuron.2	5.1	165.1	46.0	55	10	29	8	
Fipronil.1	9.7	437.1	368.0	96	10	23	9	Fipronil-13C3.1
Fipronil.2	9.7	437.1	290.0	96	10	37	7	Fipronil-13C3.1
Flamprop-isopropyl.1	10.2	364.1	105.0	65	10	50	12	
Flamprop-isopropyl.2	10.2	364.1	304.0	65	10	20	12	
Flamprop-methyl.1	8.9	336.0	105.0	65	10	46	12	
Flamprop-methyl.2	8.9	336.0	77.0	65	10	50	12	
Flazasulfuron.1	5.3	408.1	182.1	65	10	35	12	
Flazasulfuron.2	5.3	408.1	139.0	65	10	60	12	
Flonicamid.1	4.1	230.1	203.1	71	10	23	12	
Flonicamid.2	4.1	230.1	174.0	71	10	25	10	
Florasulam.1	5.1	360.0	129.1	65	10	50	12	
Florasulam.2	5.1	360.0	109.0	65	10	20	12	
Fluazifop.1	7.0	328.1	282.0	65	10	35	12	
Fluazifop.2	7.0	328.1	254.0	65	10	43	12	
Fluazuron.1	12.2	505.9	348.9	151	10	27	30	
Fluazuron.2	12.2	505.9	140.9	151	10	83	20	
Flubendiamide.1	9.9	683.1	408.0	85	10	9	12	
Flubendiamide.2	9.9	683.1	274.1	85	10	41	6	
Fludioxinil.1	8.4	266.0	229.0	41	10	17	14	
Fludioxinil.2	8.4	266.0	227.1	41	10	11	12	
Flufenacet.1	9.3	364.1	152.1	36	10	27	8	
Flufenacet.2	9.3	364.1	194.2	36	10	17	12	
Flufenoxuron.1	12.4	489.0	158.0	96	10	27	10	

Flufenoxuron.2	12.4	489.0	141.1	96	10	63	8	
Fluometuron.1	7.2	233.1	72.1	76	10	35	12	
Fluometuron.2	7.2	233.1	46.0	76	10	37	8	
Fluopicolide.1	8.7	383.0	173.0	66	10	31	14	
Fluopicolide.2	8.7	383.0	145.0	66	10	71	12	
Fluopyram.1	9.1	396.7	208.0	15	10	29	24	Fluopyram-d4.1
Fluopyram.2	9.1	396.7	173.0	15	10	35	20	Fluopyram-d4.1
Fluoxastrobin.1	9.1	459.2	427.2	81	10	23	12	
Fluoxastrobin.2	9.1	459.2	188.0	81	10	47	10	
Fluquinconazole.1	9.1	376.0	307.0	86	10	27	16	
Fluquinconazole.2	9.1	376.0	349.0	86	10	29	20	
Fluridone.1	8.0	330.1	309.1	65	10	50	12	
Fluridone.2	8.0	330.1	290.0	65	10	50	12	
Fluroxypyr.1	4.2	254.9	208.8	65	10	22	12	
Fluroxypyr.2	4.2	254.9	180.8	65	10	28	12	
Flurtamone.1	8.0	334.1	247.2	65	10	35	12	
Flurtamone.2	8.0	334.1	178.2	65	10	50	12	
Flusilazole.1	9.6	316.1	165.1	86	10	37	10	
Flusilazole.2	9.6	316.1	247.1	86	10	21	18	
Fluthiacet-methyl.1	9.9	404.0	274.2	65	10	39	12	
Fluthiacet-methyl.2	9.9	404.0	215.0	65	10	50	12	
Flutolanil.1	8.7	324.1	242.1	75	10	35	14	
Flutolanil.2	8.7	324.1	262.1	75	10	31	16	
Flutriafol.1	7.5	302.1	70.1	16	10	59	2	
Flutriafol.2	7.5	302.1	123.0	16	10	37	2	
Fluxapyroxad.1	8.7	382.0	362.0	156	10	21	28	
Fluxapyroxad.2	8.7	382.0	342.0	156	10	27	54	
FomesafenNH4.1	7.7	456.1	344.0	65	10	23	12	

FomesafenNH4.2	7.7	456.1	223.1	65	10	46	12	
Fonofos.1	10.3	247.0	137.0	65	10	15	12	
Fonofos.2	10.3	247.0	109.0	65	10	35	12	
Foramsulfuron.1	5.2	453.0	182.0	65	10	31	12	
Foramsulfuron.2	5.2	453.0	272.0	65	10	20	12	
Forchlorfenuron.1	7.6	248.0	129.1	71	10	25	8	
Forchlorfenuron.2	7.6	248.0	93.1	71	10	49	6	
Formetanate HCl.1	4.4	222.1	165.0	66	10	23	10	
Formetanate HCl.2	4.4	222.1	120.0	66	10	37	8	
Fosthiazat.1	7.2	284.1	227.9	56	10	15	14	
Fosthiazat.2	7.2	284.1	104.0	56	10	31	6	
Fuberidazole.1	6.5	185.0	157.0	86	10	37	8	
Fuberidazole.2	6.5	185.0	65.0	86	10	57	12	
Furalaxyl.1	8.2	302.1	242.1	51	10	21	16	
Furalaxyl.2	8.2	302.1	95.0	51	10	39	8	
Furathiocarb.1	11.6	383.1	195.1	86	10	25	12	
Furathiocarb.2	11.6	383.1	252.1	86	10	17	6	
Halofenozide.1	8.4	331.2	275.1	30	10	11	8	
Halofenozide.2	8.4	331.2	105.0	30	10	23	6	
Haloxifop.1	8.1	362.2	316.2	65	10	35	12	
Haloxifop.2	8.1	362.2	272.2	65	10	50	12	
Haloxyfop-2-ethoxyethyl.1	11.5	434.0	316.0	65	10	35	12	
Haloxyfop-2-ethoxyethyl.2	11.5	434.0	272.0	65	10	50	12	
Haloxyfop-P-methyl.1	11.0	376.0	316.0	65	10	35	12	
Haloxyfop-P-methyl.2	11.0	376.0	272.0	65	10	50	12	
Heptenophos.1	7.7	251.0	127.0	65	10	25	12	
Heptenophos.2	7.7	251.0	125.0	65	10	32	12	
Hexaconazole.1	10.4	314.1	70.0	81	10	55	6	

Hexaconazole.2	10.4	314.1	159.0	81	10	33	10	
Hexaflumuron.1	11.1	461.1	158.2	85	10	23	10	
Hexaflumuron.2	11.1	461.1	141.1	85	10	57	8	
Hexazionon(Velpar).1	6.8	253.1	171.1	116	10	23	10	
Hexazionon(Velpar).2	6.8	253.1	71.0	116	10	41	10	
Hexythiazox.1	12.2	353.1	228.0	41	10	19	14	
Hexythiazox.2	12.2	353.1	168.0	41	10	37	12	
Hydramethylnon.1	11.1	495.2	323.2	136	10	41	10	
Hydramethylnon.2	11.1	495.2	151.1	136	10	77	2	
Hydroxycarbofuran.1	5.3	238.0	181.1	106	10	15	10	
Hydroxycarbofuran.2	5.3	238.0	163.0	106	10	21	20	
Imazalil.1	10.0	297.0	159.0	81	10	29	12	
Imazalil.2	10.0	297.0	201.0	81	10	25	12	
Imazaquin.1	5.0	312.0	199.1	61	10	35	12	
Imazaquin.2	5.0	312.1	181.2	61	10	50	12	
Imidacloprid.1	4.9	256.0	209.1	61	10	21	14	Imidacloprid-d4.1
Imidacloprid.2	4.9	256.0	175.1	61	10	25	10	Imidacloprid-d4.1
Indoxacarb.1	10.8	528.0	203.0	55	10	47	12	
Indoxacarb.2	10.8	528.0	218.0	55	10	35	16	
Indoxacarb.3	10.8	528.0	249.0	55	10	27	18	
Indoxacarb.4	10.8	528.0	150.0	55	10	27	20	
Ipconazole.1	11.1	334.2	70.0	81	10	37	12	
Ipconazole.2	11.1	334.2	125.0	101	10	47	12	
Iprobenfos.1	9.9	289.1	205.0	61	10	17	12	
Iprobenfos.2	9.9	289.1	91.0	61	10	35	12	
Iprodione.1	9.7	330.1	244.9	61	10	21	14	
Iprodione.2	9.7	332.1	246.9	61	10	21	14	
Iprovalicarb.1	9.2	321.2	203.1	61	10	13	14	

Iprovalicarb.2	9.2	321.2	119.0	61	10	47	8	
Isocarbamide.1	5.7	186.0	86.9	56	10	37	12	
Isocarbamide.2	5.7	186.0	130.2	56	10	20	12	
Isocarbophos.1	7.8	307.0	231.1	32	10	19	5	
Isocarbophos.2	7.8	307.0	121.1	32	10	42	7	
Isofenphos.1	10.6	346.1	217.1	61	10	35	12	
Isofenphos.2	10.6	346.1	245.1	61	10	25	12	
Isofenphos-methyl Na.1	9.9	354.0	252.9	156	10	23	16	
Isofenphos-methyl Na.2	9.9	354.0	311.8	156	10	21	22	
Isofenphos-methyl.1	9.9	332.1	231.0	61	10	20	12	
Isofenphos-methyl.2	9.9	332.1	273.0	61	10	17	12	
Isofenphos-methyl.3	9.9	332.0	231.0	1	10	19	22	
Isofenphos-methyl.4	9.9	332.0	272.9	1	10	7	22	
Isoprocarb.1	7.5	194.1	137.0	56	10	13	8	
Isoprocarb.2	7.5	194.1	95.0	56	10	21	6	
Isoprothiolane.1	8.7	291.1	189.0	76.02	10	36	12	
Isoprothiolane.2	8.7	291.1	145.0	76.01	10	50	12	
Isoproturon.1	7.6	207.2	72.1	71	10	29	12	
Isoproturon.2	7.6	207.2	46.1	71	10	35	8	
Isoxaflutole.1	7.6	359.9	250.9	136	10	23	28	
Isoxaflutole.2	7.6	359.9	220.0	136	10	51	22	
Isoxathion.1	10.5	314.1	105.0	61	10	32	12	
Isoxathion.2	10.5	314.1	96.9	61	10	50	12	
Ivermectin.1	14.3	892.3	569.5	56	10	21	16	
Ivermectin.2	14.3	892.3	307.3	56	10	33	8	
izoxaben.1	8.6	333.1	150.1	65	10	50	12	
izoxaben.2	8.6	333.1	165.0	65	10	35	12	
Kresoxim-methyl.1	10.0	314.2	116.1	63	10	18	6	

Kresoxim-methyl.2	10.0	314.2	131.2	63	10	29	8	
Lenacil.1	7.5	235.1	153.0	41	10	21	18	
Lenacil.2	7.5	235.1	136.0	41	10	43	16	
Linuron.1	8.3	249.1	182.1	66	10	21	12	
Linuron.2	8.3	249.1	160.0	66	10	25	34	
Lufenuron.1	11.9	511.1	158.1	96	10	27	10	
Lufenuron.2	11.9	511.1	141.2	96	10	61	8	
Malaoxon.1	6.8	315.1	99.1	61	10	37	6	
Malaoxon.2	6.8	315.1	127.1	61	10	19	8	
Malathion.1	8.7	331.0	285.0	61	10	11	12	Malathion-d6.1
Malathion.2	8.7	331.0	127.0	61	10	19	12	Malathion-d6.1
Mandipropamid.1	8.5	412.1	328.1	85	10	19	8	
Mandipropamid.2	8.5	412.1	356.1	85	10	15	10	
Mecarbam.1	9.3	330.1	227.0	61	10	20	12	
Mecarbam.2	9.3	330.1	198.9	61	10	26	12	
Mefenacet.1	8.9	299.0	148.1	36	10	21	8	
Mefenacet.2	8.9	299.0	120.1	36	10	35	6	
Mefenpyr-diethyl.1	10.4	390.1	327.0	61	10	26	12	
Mefenpyr-diethyl.2	10.4	390.1	160.1	61	10	46	12	
Mepanipyrim.1	9.2	224.0	106.0	86	10	35	6	
Mepanipyrim.2	9.2	224.0	77.0	86	10	55	6	
Mephosfolan.1	6.6	270.1	140.0	65	10	34	12	
Mephosfolan.2	6.6	270.1	75.1	65	10	48	12	
Mepronil.1	8.8	270.1	119.1	81	10	31	6	
Mepronil.2	8.8	270.1	228.0	81	10	21	16	
Mesotrione.1	3.1	340.2	228.2	85	10	22	5	
Mesotrione.2	3.1	340.2	104.1	85	10	41	6	
Metaflumizone.1	11.7	507.1	178.1	101	10	33	10	

Metaflumizone.2	11.7	507.1	287.1	101	10	33	6	
Metalaxyl.1	7.6	280.1	220.2	61	10	19	14	
Metalaxyl.2	7.6	280.1	192.2	61	10	25	12	
Metamitron.1	5.2	203.1	175.1	86	10	21	20	
Metamitron.2	5.2	203.1	104.0	86	10	29	12	
Metazachlor.1	7.5	278.0	134.1	80	10	27	16	
Metazachlor.2	7.5	278.0	210.0	80	10	13	24	
Metconazole.1	10.5	320.1	70.0	101	10	43	4	
Metconazole.2	10.5	320.1	125.0	101	10	53	4	
Methabenzthiazuron.1	7.5	222.1	150.3	46	10	45	8	
Methabenzthiazuron.2	7.5	222.1	165.2	46	10	21	10	
Methacrifos.1	7.4	241.0	124.9	61	10	35	12	
Methacrifos.2	7.4	241.0	208.9	61	10	17	12	
Methamidophos.1	1.9	141.9	93.9	51	10	25	6	Methamidophos-d6.1
Methamidophos.2	1.9	142.0	125.0	51	10	19	6	Methamidophos-d6.1
Methidathion.1	7.9	303.0	145.1	66	10	15	12	
Methidathion.2	7.9	303.0	85.1	66	10	27	12	
Methiocarb.1	8.4	226.1	169.1	56	10	13	10	
Methiocarb.2	8.4	226.1	121.1	56	10	27	6	
Methiocarb-sulfone.1	5.2	258.1	200.9	61	10	13	12	
Methiocarb-sulfone.2	5.2	258.1	122.0	61	10	25	8	
Methomyl.1	4.0	163.1	88.1	41	10	13	4	
Methomyl.2	4.0	163.1	106.0	41	10	13	6	
Methoprotryne.1	8.3	272.2	240.2	41	10	27	16	
Methoprotryne.2	8.3	272.2	198.0	41	10	31	12	
Methoxyfenozide.1	8.8	369.1	313.2	56	10	11	4	
Methoxyfenozide.2	8.8	369.1	149.1	56	10	21	8	
Metobromuron.1	7.4	259.0	170.2	60	10	25	10	

Metobromuron.2	7.4	259.0	148.2	60	10	21	8	
Metolachlor.1	9.5	284.1	252.0	65	10	20	12	Metolachlor-d6.1
Metolachlor.2	9.5	284.1	176.2	65	10	35	12	Metolachlor-d6.1
Metolcarb.1	6.4	166.0	108.9	76	10	13	10	
Metolcarb.2	6.4	166.0	94.0	76	10	39	10	
Metosulam.1	6.3	418.0	175.1	61	10	41	12	
Metosulam.2	6.3	418.0	140.1	61	10	55	12	
Metoxuron.1	6.1	229.0	72.0	70	10	19	8	
Metoxuron.2	6.1	229.0	156.0	70	10	31	16	
Metrafenon.1	10.7	409.2	209.1	51	10	19	12	
Metrafenon.2	10.7	411.2	209.1	51	10	19	12	
Metribuzin.1	6.8	215.1	187.1	41	10	25	12	
Metribuzin.2	6.8	215.1	84.1	41	10	31	6	
Metsulfuron-methyl.1	4.7	382.1	167.1	65	10	35	12	
Metsulfuron-methyl.2	4.7	382.1	135.1	65	10	55	12	
Mevinphos-Isomer.1.1	5.3	225.1	127.1	56	10	21	8	
Mevinphos-Isomer.1.2	5.3	225.1	193.2	56	10	11	12	
Mevinphos-Isomer.2.1	5.9	225.1	127.1	56	10	21	8	
Mevinphos-Isomer.2.2	5.9	225.1	193.2	56	10	11	12	
Mexacarbate.1	8.8	223.2	166.1	56	10	21	10	
Mexacarbate.2	8.8	223.2	151.0	56	10	31	10	
Molinate.1	9.0	188.2	126.2	65	10	25	10	
Molinate.2	9.0	188.2	98.0	65	10	45	10	
(Monceren)Pencycuron.1	10.7	329.1	125.0	71	10	31	8	
(Monceren)Pencycuron.2	10.7	329.1	218.1	71	10	23	14	
Monocrotophos.1	4.5	224.1	127.1	61	10	21	8	
Monocrotophos.2	4.5	224.1	98.0	61	10	17	6	
Monolinuron.1	7.1	215.1	126.1	61	10	23	8	

Monolinuron.2	7.1	215.1	99.0	61	10	47	8	
Monuron.1	6.6	199.0	126.0	71	10	42	12	
Monuron.2	6.6	199.0	99.0	71	10	50	12	
Moxidectin.1	14.0	640.4	528.5	61	10	13	18	
Moxidectin.2	14.0	640.4	498.5	61	10	17	16	
Myclobutanil.1	8.8	289.0	70.0	66	10	41	4	
Myclobutanil.2	8.8	289.0	125.0	66	10	39	8	
Napropamide.1	9.4	272.2	171.0	56	10	35	12	Napropamide-d10.1
Napropamide.2	9.4	272.2	128.0	56	10	53	12	Napropamide-d10.1
Neburon.1	9.8	275.0	88.0	26	10	23	6	
Neburon.2	9.8	275.0	114.0	26	10	21	6	
Nitenpyram.1	3.8	271.0	126.0	66	10	35	8	
Nitenpyram.2	3.8	271.0	225.2	66	10	17	14	
Nitralin.1	9.6	346.1	304.1	56	10	20	12	
Nitralin.2	9.6	346.1	262.1	56	10	16	12	
Norflurazon.1	7.7	304.0	284.1	65	10	39	10	
Norflurazon.2	7.7	304.0	160.1	65	10	50	10	
Novaluron.1	11.1	493.0	158.1	66	10	29	10	
Novaluron.2	11.1	493.0	141.1	66	10	65	8	
Nuarimol.1	8.3	315.0	252.1	101	10	37	22	
Nuarimol.2	8.3	315.0	81.0	101	10	49	6	
Omethoate.1	3.0	214.0	182.8	56	10	17	12	
Omethoate.2	3.0	214.0	124.9	56	10	31	8	
Oxadiazon.1	11.9	345.0	303.0	146	10	19	16	
Oxadiazon.2	11.9	345.0	219.9	146	10	27	12	
Oxadixyl.1	6.3	279.1	132.1	66	10	43	8	
Oxadixyl.2	6.3	279.1	219.1	66	10	15	14	
Oxamyl.1	3.8	237.1	90.1	26	10	11	6	

Oxamyl.2	3.8	237.1	72.1	26	10	29	4	
Oxamyl-oxime.1	3.0	163.0	71.9	65	10	52	10	
Oxamyl-oxime.2	3.0	163.0	90.0	65	10	35	10	
Oxycarboxin.1	5.7	268.1	174.9	65	10	52	10	
Oxycarboxin.2	5.7	268.1	104.9	65	10	49	10	
Oxydemeton-methyl.1	4.1	247.0	168.9	51	10	21	14	
Oxydemeton-methyl.2	4.1	247.0	108.9	51	10	39	18	
Paclobutrazol.1	8.7	294.0	70.0	21	10	49	12	
Paclobutrazol.2	8.7	294.0	125.0	21	10	41	12	
Paraoxon.1	7.4	275.9	219.9	65	10	20	12	
Paraoxon.2	7.4	275.9	174.1	65	10	35	12	
Paraoxon-ethyl.1	7.4	276.0	219.9	46	10	21	26	
Paraoxon-ethyl.2	7.4	276.0	174.0	46	10	33	20	
Paraoxon-methyl.1	6.3	248.0	202.0	76	10	27	12	
Paraoxon-methyl.2	6.3	248.1	109.0	76	10	39	18	
Parathion.1	9.9	292.0	235.9	66	10	21	14	
Parathion.2	9.9	292.0	110.0	66	10	41	14	
Parathion-methyl.1	8.3	263.9	232.1	71	10	21	12	
Parathion-methyl.2	8.3	263.9	125.0	71	10	38	12	
Penconazole.1	9.9	284.1	70.0	81	10	37	2	
Penconazole.2	9.9	284.1	159.0	81	10	35	16	
Pendimethalin.1	12.3	282.1	212.2	46	10	15	12	Pendimethalin-d5.1
Pendimethalin.2	12.3	282.1	194.0	46	10	23	12	Pendimethalin-d5.1
Penthiopyrad.1	9.9	360.0	275.9	136	10	19	18	
Penthiopyrad.2	9.9	360.0	177.0	136	10	41	20	
Pethoxamid.1	9.4	296.2	131.0	65	10	35	12	
Pethoxamid.2	9.4	296.2	91.0	65	10	50	12	
Phenmedipham.1	7.9	301.2	168.0	76	10	12	10	

Phenmedipham.2	7.9	301.2	107.9	76	10	44	6	
Phenothrin.1	13.8	351.1	183.0	71	10	31	12	
Phenothrin.2	13.8	351.1	128.0	71	10	83	12	
Phenthoate.1	9.9	321.0	247.0	65	10	18	12	
Phenthoate.2	9.9	321.0	135.1	65	10	35	12	
Phenylthiourea.1	3.9	153.0	135.9	1	10	21	12	
Phenylthiourea.2	3.9	153.0	77.2	1	10	33	4	
Phorate-sulfone.1	7.4	293.0	170.8	65	10	20	12	
Phorate-sulfone.2	7.4	293.0	96.7	65	10	48	12	
Phorate-sulfoxide.1	7.3	277.0	143.0	65	10	35	12	
Phorate-sulfoxide.2	7.3	277.0	171.0	65	10	25	12	
Phosalone.1	10.6	368.0	182.0	65	10	24	12	
Phosalone.2	10.6	369.9	111.0	65	10	50	12	
Phosmet.1	7.7	318.0	160.1	71	10	19	12	
Phosmet.2	7.7	318.0	133.1	71	10	49	12	
Phosphamidon.1	6.5	300.2	127.1	65	10	42	12	
Phosphamidon.2	6.5	300.2	174.0	65	10	35	12	
Phoxim.1	10.5	299.2	129.2	56	10	17	8	
Phoxim.2	10.5	299.2	77.1	56	10	42	12	
Picoxystrobin.1	9.7	368.0	205.0	56	10	13	14	
Picoxystrobin.2	9.7	368.0	145.0	56	10	29	8	
Pinoxaden.1	10.5	401.1	317.1	141	10	27	34	
Pinoxaden.2	10.5	401.1	57.0	141	10	65	26	
Piperonyl-butoxide.1	11.9	356.2	177.2	41	10	13	10	
Piperonyl-butoxide.2	11.9	356.2	119.1	41	10	47	6	
Piperophos.1	11.0	354.1	171.0	65	10	26	12	
Piperophos.2	11.0	354.1	143.0	65	10	35	12	
Pirimicarb.1	7.4	239.2	72.1	61	10	33	4	

Pirimicarb.2	7.4	239.2	182.1	61	10	21	12	
Pirimicarb-desmethyl.1	6.2	225.0	72.0	56	10	27	12	
Pirimicarb-desmethyl.2	6.2	225.0	168.1	56	10	19	12	
Pirimiphos-ethyl.1	11.9	334.1	198.0	65	10	38	12	
Pirimiphos-ethyl.2	11.9	334.1	182.3	65	10	42	12	
Pirimiphos-methyl.1	10.7	306.1	164.1	66	10	29	12	
Pirimiphos-methyl.2	10.7	306.1	108.1	66	10	39	12	
Pirimiszulfuron-metil.1	7.1	469.0	254.0	65	10	29	12	
Pirimiszulfuron-metil.2	7.1	469.0	135.1	65	10	50	12	
Prochloraz.1	10.5	376.0	308.0	51	10	15	8	
Prochloraz.2	10.5	376.0	70.0	51	10	43	4	
Prochloraz-desimidazole-amino.1	10.4	325.0	129.0	136	10	33	26	
Prochloraz-desimidazole-amino.2	10.4	325.0	282.0	136	10	25	14	
Profluralin.1	7.4	348.0	276.0	1	10	13	14	
Profluralin.2	7.4	348.0	248.0	1	10	19	14	
Promecarb.1	8.6	208.1	151.0	56	10	13	10	
Promecarb.2	8.6	208.1	109.0	56	10	21	6	
Prometon.1	8.2	226.1	142.0	81	10	33	8	
Prometon.2	8.2	226.1	86.0	81	10	39	6	
Prometryne.1	9.2	242.2	158.1	41	10	33	10	
Prometryne.2	9.2	242.2	200.1	41	10	25	12	
Propachlor.1	7.6	212.0	170.0	65	10	25	12	
Propachlor.2	7.6	212.0	94.1	65	10	47	12	
Propamocarb.1	4.0	189.2	102.0	61	10	25	6	
Propamocarb.2	4.0	189.2	144.0	61	10	19	8	
Propanil.1	8.3	218.0	127.0	71	10	50	12	
Propanil.2	8.3	218.0	162.0	71	10	35	12	
Propargite.1	12.5	368.2	231.1	41	10	15	16	

Propargite.2	12.5	368.2	175.1	41	10	23	10	
Propetamphos.1	8.9	282.1	138.0	65	10	30	12	
Propetamphos.2	8.9	282.1	110.0	65	10	45	12	
Propham.1	7.4	180.1	138.0	46	10	11	8	
Propham.2	7.4	180.1	120.0	46	10	23	6	
Propiconazole.1	10.2	342.1	159.0	81	10	31	8	
Propiconazole.2	10.2	342.1	69.0	81	10	39	6	
Propoxur.1	6.7	210.1	111.0	51	10	19	6	
Propoxur.2	6.7	210.1	168.1	51	10	11	10	
Propyzamide.1	8.8	256.1	190.0	65	10	28	12	
Propyzamide.2	8.8	256.1	173.0	65	10	32	12	
Proquinazid.1	12.9	373.2	330.9	60	10	16	12	
Proquinazid.2	12.9	373.2	289.0	60	10	20	12	
Prosulfocarb.1	11.3	252.1	91.2	76	10	29	12	
Prosulfocarb.2	11.3	252.1	128.1	76	10	17	12	
Prosulfuron.1	6.7	420.1	167.1	65	10	32	12	
Prosulfuron.2	6.7	420.1	141.0	65	10	41	12	
Prothioconazole.1	9.7	344.1	188.9	46	10	31	12	
Prothioconazole.2	9.7	344.1	125.1	46	10	33	13	
Prothiofos.1	13.2	344.9	240.9	126	10	25	28	
Prothiofos.2	13.2	344.9	132.9	126	10	65	16	
Pymetrozine.1	4.3	218.0	105.0	71	10	27	6	
Pymetrozine.2	4.3	218.0	78.0	71	10	61	4	
Pyracarbolid.1	6.9	218.1	125.0	61	10	25	8	
Pyracarbolid.2	6.9	218.1	97.0	61	10	37	8	
Pyraclostrobin.1	10.4	388.0	194.0	51	10	17	14	Pyraclostrobin-d3.1
Pyraclostrobin.2	10.4	388.0	163.0	51	10	31	10	Pyraclostrobin-d3.1
Pyraflufen-ethyl.1	10.1	414.9	340.9	116	10	27	18	

Pyraflufen-ethyl.2	10.1	414.9	262.9	116	10	43	30	
Pyrazophos.1	10.6	374.0	222.0	60	10	32	12	
Pyrazophos.2	10.6	374.0	194.0	60	10	45	12	
Pyrethrin.1	12.6	329.1	161.1	136	10	13	8	
Pyrethrin.2	12.6	329.1	77.0	136	10	81	8	
Pyridaben.1	13.1	365.0	147.0	41	10	33	8	
Pyridaben.2	13.1	365.0	309.0	41	10	19	8	
Pyridafol.1	4.3	207.0	103.9	65	10	39	12	
Pyridafol.2	4.3	207.0	77.0	65	10	45	12	
Pyridaphenthion.1	8.9	341.0	189.0	56	10	36	12	
Pyridaphenthion.2	8.9	341.0	205.0	56	10	32	12	
Pyrifenox.1	9.6	295.1	93.1	76	10	42	12	
Pyrifenox.2	9.6	295.1	67.2	76	10	80	12	
Pyrimethanil.1	8.5	200.0	107.0	86	10	33	6	Pyrimethanil-d5.1
Pyrimethanil.2	8.5	200.0	82.0	86	10	37	4	Pyrimethanil-d5.1
Pyriproxyfen.1	12.1	322.0	96.0	46	10	21	6	
Pyriproxyfen.2	12.1	322.0	185.0	46	10	31	12	
Pyroquilon.1	6.6	174.0	117.0	65	10	32	12	
Pyroquilon.2	6.6	174.0	132.0	65	10	25	12	
Quinalphos.1	9.9	299.0	163.0	65	10	32	12	
Quinalphos.2	9.9	299.0	271.0	65	10	20	12	
Quinoclamine.1	6.5	208.0	77.0	65	10	55	12	
Quinoclamine.2	6.5	208.0	105.0	65	10	41	12	
Quinoxyfen.1	12.1	308.1	197.1	91	10	45	12	
Quinoxyfen.2	12.1	308.1	162.1	91	10	63	10	
Quizalofop.1	7.8	345.1	299.0	71	10	25	15	
Quizalofop.2	7.8	347.1	91.0	71	10	47	15	
Resmethrin.1	13.5	339.1	128.0	116	10	61	14	

Resmethrin.2	13.5	339.1	91.0	116	10	67	14	
Rimsulfuron.1	4.8	432.0	182.1	131	10	27	10	
Rimsulfuron.2	4.8	432.0	325.0	131	10	21	22	
Rotenone.1	9.6	395.1	213.1	101	10	31	14	
Rotenone.2	9.6	395.1	192.1	101	10	33	12	
Sebuthylazin.1	8.2	230.1	174.2	65	10	35	12	
Sebuthylazin.2	8.2	230.1	68.0	65	10	55	12	
Secbumeton.1	8.4	226.2	170.1	86	10	25	10	
Secbumeton.2	8.4	226.2	100.0	86	10	37	6	
Silthiofam.1	9.8	268.0	139.1	65	10	31	12	
Silthiofam.2	9.8	268.0	73.0	65	10	50	12	
Simazine.1	6.7	202.2	104.0	91	10	33	12	
Simazine.2	6.7	202.2	124.0	91	10	30	12	
Simetryn.1	7.5	214.0	124.0	36	10	29	10	
Simetryn.2	7.5	214.0	144.0	36	10	29	8	
Spinetoram.1	13.7	748.5	142.2	146	10	43	8	
Spinetoram.2	13.7	748.5	98.1	146	10	95	8	
Spinosad (Spinosyn A).1	13.3	732.5	142.2	136	10	39	14	
Spinosad (Spinosyn A).2	13.3	732.5	98.1	136	10	95	6	
Spinosad (Spinosyn D).1	13.7	746.8	142.4	112	11	41	11	
Spinosad (Spinosyn D).2	13.7	746.8	98.3	112	11	93	7	
Spirodiclofen.1	13.0	411.3	71.3	101	10	31	14	
Spirodiclofen.2	13.0	411.3	313.3	101	10	17	6	
Spiromesifen.1	12.2	371.2	273.2	76	10	20	8	
Spiromesifen.2	12.2	371.2	255.2	76	10	39	6	
Spirotetramat.1	9.2	374.2	330.2	136	10	23	8	
Spirotetramat.2	9.2	374.2	302.2	136	10	27	20	
Spirotetramat-cisenol.1	7.8	300.0	268.0	276	10	39	30	

Spirotetramat-cisenol.2	7.8	300.0	214.0	276	10	37	24	
Spiroxamine-Isomer.1.1	11.0	298.2	144.2	71	10	29	8	
Spiroxamine-Isomer.1.2	11.0	298.2	100.1	71	10	43	6	
Sulfentrazone.1	6.7	387.0	307.1	96	10	29	20	
Sulfentrazone.2	6.7	387.0	146.0	96	10	59	8	
Sulfometuron-methyl.1	5.3	365.0	150.0	65	10	26	12	
Sulfometuron-methyl.2	5.3	365.0	107.0	65	10	49	12	
Sulfosulfuron.1	5.4	471.1	211.2	65	10	38	12	
Sulfosulfuron.2	5.4	471.1	156.0	65	10	50	12	
Sulfotep(Tetraethyldithiopyrophosphate).1	8.2	323.0	97.0	65	10	48	12	
Sulfotep(Tetraethyldithiopyrophosphate).2	8.2	323.0	115.0	65	10	31	12	
Tau-fluvalinate.1	13.4	503.0	181.0	65	10	39	12	
Tau-fluvalinate.2	13.4	503.0	208.0	65	10	32	12	
Tebuconazole.1	10.0	308.2	70.0	86	10	51	10	
Tebuconazole.2	10.0	308.2	125.0	86	10	55	8	
Tebufenozide.1	9.7	353.2	297.2	51	10	11	8	
Tebufenozide.2	9.7	353.2	133.0	51	10	23	8	
Tebufenpyrad.1	11.7	334.0	145.0	116	10	37	8	
Tebufenpyrad.2	11.7	334.0	117.0	116	10	47	8	
Tebutam.1	9.5	234.2	91.1	65	10	48	12	
Tebutam.2	9.5	234.2	192.4	65	10	16	12	
Tebuthiuron.1	6.9	229.1	172.4	66	10	25	10	
Tebuthiuron.2	6.9	229.1	116.1	66	10	37	6	
Teflubenzuron.1	11.8	381.1	158.2	106	10	23	10	
Teflubenzuron.2	11.8	381.1	141.2	106	10	47	8	
Tembotrion.1	5.8	460.0	343.0	15	10	21	20	
Tembotrion.2	5.8	460.0	264.0	15	10	43	30	
Temephos.1	11.8	467.0	419.1	96	10	27	12	

Temephos.2	11.8	467.0	405.0	96	10	21	12	
Terbufos-sulfone.1	8.2	321.1	115.0	66	10	35	10	
Terbufos-sulfone.2	8.2	321.1	171.0	66	10	25	10	
Terbufos-sulfoxide.1	8.2	305.0	187.0	71	10	27	12	
Terbufos-sulfoxide.2	8.2	305.0	97.0	71	10	47	12	
Terbumeton.1	8.4	226.1	170.1	41	10	23	10	
Terbumeton.2	8.4	226.1	100.0	41	10	41	6	
Terbuthylazine-desethyl.1	7.0	202.1	146.1	65	10	20	12	
Terbuthylazine-desethyl.2	7.0	202.1	79.1	65	10	35	12	
Terbutryn.1	9.4	242.1	186.1	36	10	25	12	
Terbutryn.2	9.4	242.1	68.1	36	10	61	4	
Tetrachlorvinphos.1	9.8	367.0	127.0	65	10	48	12	
Tetrachlorvinphos.2	9.8	367.0	206.0	65	10	39	12	
Tetraconazole.1	9.2	372.1	159.0	86	10	35	8	
Tetraconazole.2	9.2	372.1	70.0	86	10	47	12	
Tetrametrin NH4.1	11.3	349.2	164.1	60	10	35	8	
Tetrametrin NH4.2	11.3	349.2	107.1	60	10	50	8	
Thiabendazole.1	6.3	202.1	175.1	36	10	35	10	
Thiabendazole.2	6.3	202.1	131.2	36	10	45	8	
Thiacloprid.1	5.7	253.0	126.0	76	10	29	8	
Thiacloprid.2	5.7	253.0	99.0	76	10	59	6	
Thiamethoxam.1	4.2	292.0	211.0	56	10	17	14	Thiamethoxam-d3
Thiamethoxam.2	4.2	292.0	181.0	56	10	31	12	Thiamethoxam-d3
Thidiazuron.1	6.7	221.1	102.1	36	10	23	6	
Thidiazuron.2	6.7	221.1	127.9	36	10	23	8	
Thifensulfuron-methyl.1	4.7	388.0	167.1	65	10	35	12	
Thifensulfuron-methyl.2	4.7	388.0	205.1	65	10	25	12	
Thiodicarb.1	7.1	355.0	88.0	61	10	31	6	

Thiodicarb.2	7.1	355.0	108.0	61	10	23	8	
Thiofanox.1	7.3	219.2	57.2	28	10	16	2	
Thiofanox.2	7.3	219.2	61.1	28	10	13	2	
Thiofanox-sulfone Na.1	5.1	273.0	215.9	141	10	19	22	
Thiofanox-sulfone Na.2	5.1	273.0	137.1	141	10	29	14	
Thiofanox-sulfone NH4.1	5.1	268.0	251.0	1	10	9	22	
Thiofanox-sulfone NH4.2	5.1	268.0	57.1	1	10	35	14	
Thiofanox-sulfone.1	5.1	251.1	57.2	61	10	40	12	
Thiofanox-sulfone.2	5.1	251.1	76.1	61	10	32	12	
Thiofanox-sulfone.3	5.1	251.0	56.9	96	10	23	26	
Thiofanox-sulfone.4	5.1	251.0	75.9	96	10	9	12	
Thiofanox-sulfoxide NH4.1	4.9	252.0	235.0	1	10	9	16	
Thiofanox-sulfoxide NH4.2	4.9	252.0	104.0	1	10	15	12	
Thiofanox-sulfoxide.1	4.9	235.0	103.9	101	10	11	12	
Thiofanox-sulfoxide.2	4.9	235.0	57.1	101	10	27	26	
Thiofanox-sulfoxide.3	4.9	235.0	104.0	91	10	11	14	
Thiofanox-sulfoxide.4	4.9	235.0	57.0	91	10	27	8	
Thionazin(zinophos).1	7.6	249.0	193.0	80	10	23	10	
Thionazin(zinophos).2	7.6	249.0	97.0	80	10	40	10	
Thiophanate.1	7.7	371.1	151.1	65	10	50	12	
Thiophanate.2	7.7	371.1	325.1	65	10	15	12	
Thiophanate-methyl.1	6.7	343.0	151.1	61	10	31	10	
Thiophanate-methyl.2	6.7	343.0	311.0	61	10	17	8	
Tolclofos-methyl.1	10.6	301.0	268.9	86	10	23	10	
Tolclofos-methyl.2	10.6	301.0	175.0	86	10	35	10	
Tolfenpyrad.1	11.8	384.0	197.0	191	10	47	18	
Tolfenpyrad.2	11.8	384.0	154.0	191	10	49	18	
Tralkoxydim.1	9.0	330.2	138.1	65	10	35	12	

Tralkoxydim.2	9.0	330.2	96.0	65	10	50	12	
Triadimefon.1	8.8	294.0	197.1	41	10	21	12	
Triadimefon.2	8.8	294.0	225.0	41	10	19	14	
Triadimenol.1	9.1	296.1	70.0	11	10	33	12	
Triadimenol.2	9.1	296.1	227.1	11	10	17	16	
Triallate.1	12.3	304.0	142.9	76	10	35	12	
Triallate.2	12.3	304.0	82.9	76	10	50	12	
Triasulforon.1	5.5	402.1	167.1	65	10	32	12	
Triasulforon.2	5.5	402.1	141.1	65	10	39	12	
Triazophos.1	9.0	314.0	162.0	65	10	38	12	
Triazophos.2	9.0	314.2	119.0	65	10	53	12	
Trichlorfon.1	5.3	256.9	109.1	66	10	25	6	
Trichlorfon.2	5.3	256.9	79.0	66	10	50	8	
Tricyclazole.1	5.9	190.0	163.0	76	10	33	10	
Tricyclazole.2	5.9	190.0	136.0	76	10	39	8	
Trietazin.1	9.2	230.1	104.0	65	10	48	12	
Trietazin.2	9.2	230.1	67.9	65	10	52	12	
Trifloxystrobin.1	11.0	409.0	186.0	61	10	21	12	
Trifloxystrobin.2	11.0	409.0	206.0	61	10	19	14	
Triflumizole.1	11.2	346.1	278.1	16	10	17	6	
Triflumizole.2	11.2	346.1	73.0	16	10	27	4	
Triflumuron.1	10.5	359.1	156.2	71	10	23	10	
Triflumuron.2	10.5	359.1	139.0	71	10	45	8	
Triflusulfuron-methyl.1	7.0	493.1	264.2	65	10	32	12	
Triflusulfuron-methyl.2	7.0	493.1	96.0	65	10	48	12	
Trimethylphenyl-methylcarbamate.1	7.6	194.1	137.0	81	10	15	24	
Trimethylphenyl-methylcarbamate.2	7.6	194.1	107.0	81	10	51	12	
Triticonazole.1	9.3	318.1	70.0	86	10	35	6	

Triticonazole.2	9.3	318.1	125.0	86	10	49	8	
Vamidothion.1	5.3	288.0	146.0	61	10	17	8	
Vamidothion.2	5.3	288.0	118.0	61	10	37	8	
Vamidothion-sulfoxide.1	4.0	304.2	201.0	71	10	22	12	
Vamidothion-sulfoxide.2	4.0	304.2	169.0	71	10	27	12	
Cinerin I.1	12.3	317.3	149.1	41	10	11	10	
Cinerin I.2	12.3	317.3	107.0	41	10	19	12	
Cinerin I.3	12.3	317.3	91.0	41	10	51	10	
Cinerin II.1	10.6	361.3	149.2	96	10	11	18	
Cinerin II.2	10.6	361.3	107.1	96	10	19	12	
Cinerin II.3	10.6	361.3	91.0	96	10	59	10	
Jasmolin I.1	12.8	331.3	163.2	56	10	11	18	
Jasmolin I.2	12.8	331.3	107.1	56	10	29	12	
Jasmolin I.3	12.8	331.3	91.1	56	10	51	10	
Jasmolin II.1	11.3	375.3	163.2	56	10	11	18	
Jasmolin II.2	11.3	375.3	107.1	56	10	29	12	
Jasmolin II.3	11.3	375.3	91.1	56	10	51	10	
Pyrethrin I.1	12.4	329.3	161.2	141	10	11	14	
Pyrethrin I.2	12.4	329.3	105.1	141	10	45	12	
Pyrethrin I.3	12.4	329.3	128.1	141	10	53	14	
Pyrethrin II.1	10.7	373.3	161.2	141	10	11	14	
Pyrethrin II.2	10.7	373.3	105.1	141	10	45	12	
Pyrethrin II.3	10.7	373.3	128.1	141	10	53	14	
Acetamiprid-d3.1	5.3	226.0	126.0	68	10	29	8	
Atrazine-d5.1	7.5	221.0	179.0	56	10	25	15	
Atrazine-d5.2	7.5	221.0	104.0	56	10	39	15	
Atrazine-desisopropyl-d5.1	4.7	179.0	105.0	51	10	31	14	
Atrazine-desisopropyl-d5.2	4.7	179.0	104.0	51	10	31	14	

Boscalid-d4.1	8.6	347.0	311.0	35	10	27	8	
Carbaryl-d7.1	7.0	209.0	152.0	51	10	13	8	
Carbendazim-d3.1	5.8	195.0	160.2	61	10	25	10	
Carbendazim-d3.2	5.8	195.0	132.1	61	10	41	8	
Chlorotoluron-d6.1	7.3	219.0	78.0	68	10	31	4	
Chlorotoluron-d6.2	7.3	219.0	52.0	68	10	35	8	
Cyromazine-d4.1	2.9	171.0	86.0	89	10	26	4	
Cyromazine-d4.2	2.9	171.0	87.0	89	10	26	4	
Diazinon-d10.1	10.3	315.0	170.0	61	10	29	14	
Diazinon-d10.2	10.3	315.0	154.0	61	10	41	14	
Dichlorvos-d6.1	6.7	227.0	115.0	60	10	35	14	
Dichlorvos-d6.2	6.7	227.0	95.0	60	10	50	14	
Difenoconazole-d4.1	10.8	410.0	255.0	46	10	41	2	
Diflufenican-d3.1	11.1	398.0	268.0	65	10	35	12	
Diflufenican-d3.2	11.1	398.0	269.0	65	10	35	12	
Dimethoate-d6.1	5.3	236.0	205.0	46	10	13	12	
Dimethoate-d6.2	5.3	236.0	131.0	46	10	29	8	
Fipronil-13C3.1	9.7	441.0	372.0	96	10	23	9	
Fipronil-13C3.1	9.7	441.0	293.0	96	10	37	7	
Fluopyram-d4.1	9.1	402.0	209.0	15	10	29	24	
Imidacloprid-d4.1	4.9	260.0	213.0	61	10	21	14	
Imidacloprid-d4.2	4.9	260.0	212.0	61	10	21	14	
Malathion-d6.1	8.7	337.0	291.0	61	10	11	12	
Malathion-d6.2	8.7	337.0	127.0	61	10	19	12	
Methamidophos-d6.1	1.8	148.0	97.0	51	10	25	6	
Metolachlor-d6.1	9.5	290.0	258.0	65	10	20	12	
Metolachlor-d6.2	9.5	290.0	182.0	65	10	35	12	
Napropamide-d10.1	9.4	282.0	171.0	56	10	35	12	

Napropamide-d10.2	9.4	282.0	128.0	56	10	53	12	
Pendimethalin-d5.1	12.3	287.0	213.0	46	10	15	12	
Pendimethalin-d5.2	12.3	287.0	194.0	46	10	23	12	
Pyraclostrobin-d3.1	10.4	391.0	197.0	51	10	17	14	
Pyrimethanil-d5.1	8.5	205.0	108.0	86	10	33	6	
Pyrimethanil-d5.2	8.5	205.0	109.0	86	10	33	6	
Thiamethoxam-d3.1	4.2	295.0	214.0	56	10	17	14	
Thiamethoxam-d3.2	4.2	295.0	184.0	56	10	31	12	
Tebuconazole-13C3.1	10.4	311.0	70.0	86	10	51	10	
Tebuconazole-13C3.2	10.4	311.0	125.0	86	10	55	8	
Thiabendazole-d4.1	6.3	206.0	179.0	36	10	35	10	
Thiabendazole-d4.2	6.3	206.0	178.0	36	10	35	10	
TPP.1	10.5	326.9	214.9	1	10	35	24	
TPP.2	10.5	326.9	152.0	1	10	45	18	
TPP.3	10.5	326.9	77.0	1	10	71	10	
Zoxamide.1	10.2	336.1	187.0	61	10	29	12	
Zoxamide.2	10.2	336.1	159.0	61	10	55	10	

Table S2. (**a**): Validation data for 330 pesticides in groundwater: recovery% and precision (RSD%) at 10 and 100 ng/L levels. Results were calculated from ten replicates at each level. (**b**): Validation data for 330 pesticides in surface water: recovery% and precision (RSD%) at 10 and 100 ng/L levels. Results were calculated from ten replicates at each level.

(**a**)				
**Compound**	**Recovery% (10 ng/L)**	**RSD% (10 ng/L)**	**Recovery% (100 ng/L)**	**RSD% (100 ng/L)**
(Monceren)Pencycuron	92	6.57	80	2.20
3-Hydroxycarbofuran	88	6.06	93	2.58
Acephate	71	19.45	116	6.27
Acetamiprid	96	19.29	102	5.42
Acibenzolar-S-methyl	108	5.99	81	2.19
Aldicarb	108	9.68	94	2.42
Aldicarb-sulfone	110	12.46	95	3.50
Aldicarb-sulfoxide	89	12.46	96	3.21
Ametoctradin	96	8.44	89	4.58
Ametryn	86	7.24	92	4.42
Amidosulfuron	104	12.91	98	4.21
Aminocarb	88	8.91	97	4.69
Amitraz	102	6.38	86	2.32
Anilofos	88	3.42	83	2.23
Atrazine	102	5.42	102	4.37
Atrazine-desethyl	78	3.92	101	3.04
Atrazine-desisopropyl	106	4.89	96	2.90
Azaconazole	84	7.81	98	1.27
Azamethiphos	85	7.36	99	3.44
Azinphos-ethyl	115	14.36	90	2.00
Azinphos-methyl	100	10.62	92	3.40
Aziprotryne	73	10.90	92	11.77
Azoxystrobin	89	19.50	91	2.71

Benalaxyl	96	4.45	94	1.74
Bendiocarb	101	8.67	96	3.04
Benflutamid	76	8.08	93	3.69
Bensulfuron-metil	98	5.69	89	2.50
Benthiavalicarb-isopropyl	79	13.96	94	5.30
Benzolyprop-ethyl	92	9.12	97	3.13
Benzoximate	87	8.26	79	3.39
Benzthiazuron	81	7.25	79	4.20
Bifenazate	93	7.63	106	4.47
Bitertanol	95	9.19	93	3.59
Boscalid	120	10.05	97	4.92
Bromucanozole-Isomer	110	12.42	87	5.07
Bupirimate	87	6.11	92	2.56
Buprofezin	71	8.92	69	1.52
Butafenacil	104	4.18	95	2.71
Butocarboxim-sulfoxide	77	6.69	97	3.09
Butoxycarboxim	73	7.04	81	3.61
Buturon	88	19.44	97	4.19
Cadusafos	96	5.66	96	2.55
Carbaryl	90	12.82	74	2.73
Carbendazim	97	6.65	105	2.41
Carbetamide	113	6.25	108	1.87
Carbofuran	104	3.46	107	2.33
Carboxin	97	4.86	97	1.66
Carfentrazon	116	6.89	94	2.51
Carfentrazone-ethyl	79	7.03	74	4.09
Chlorantraniliprole	112	5.90	105	3.25
Chlorbromuron	64	19.96	88	5.54
Chlorfenvinphos	89	3.00	88	2.56

Chlorfluazuron	115	7.61	64	6.26
Chloridazon	107	13.84	104	4.26
Chlorotoluron	110	5.56	108	2.86
Chloroxuron	107	8.01	97	2.99
Chlorpyrifos	119	17.84	98	7.15
Chlorpyrifos-methyl	106	20.00	93	5.31
Chlorsulfuron	97	13.61	97	2.28
Chromafenozide	99	6.97	99	3.22
Cinosulfuron	99	3.05	100	2.39
Cirimidine	99	13.12	89	3.24
Clethodim-Isomer	91	16.23	81	2.40
Clomazone	96	3.56	94	2.75
Cloquintocet-mexyl	104	6.41	80	2.96
Clothianidin	111	12.44	106	2.60
Coumaphos	79	13.06	82	3.50
Crufomat(Ruelene)	78	7.71	87	4.53
Cyanazine	118	11.85	97	5.00
Cyazofamid	98	7.37	87	2.52
Cybutrine	96	11.91	99	1.46
Cyflufenamid	110	7.65	98	4.00
Cymiazole	117	5.68	96	4.22
Cymoxanil	99	12.17	106	5.12
Cyproconazole-Isomer	82	8.57	100	3.36
Cyprodinil	100	10.11	63	4.41
Cyromazine	90	10.54	102	3.52
Demeton-S	96	19.55	108	3.45
Demeton-S-methylsulphone	101	3.09	100	1.46
Demeton-S-sulfoxide	102	3.75	100	3.58
Desmedipham	115	4.53	104	3.14

Desmetryn	106	7.35	91	1.64
Diazinon	95	8.30	107	3.96
Diclobutrazol	94	6.35	100	3.45
Dicrotophos	84	5.53	83	3.29
Diethofencarb	105	5.79	104	1.99
Difenoconazole-Isomer	107	8.98	84	3.41
Difenoxuron	97	4.97	96	2.31
Diflubenzuron	99	8.92	79	3.42
Diflufenican	96	7.29	80	3.02
Dimefox	86	4.29	86	1.58
Dimefuron	70	10.56	82	4.02
Dimethachlor	91	7.58	86	4.02
Dimethenamid	100	5.38	92	2.55
Dimethirimol	97	5.63	90	1.96
Dimethoate	108	3.60	102	3.83
Dimethomorph-Isomer	98	12.38	86	5.57
Dimoxystrobin	113	3.77	99	2.44
Diniconazole	108	7.81	98	3.23
Dioxacarb	105	6.40	105	1.60
Diphenamid	97	2.90	91	1.53
Dipropetryn	86	7.29	91	3.14
Diuron	94	6.76	101	2.53
Dodemorph	89	8.34	110	7.43
Emamectin-benzoate b1a	96	4.73	110	6.93
Epoxiconazole	93	7.10	85	4.45
Etaconazole-Isomer	90	13.12	98	3.23
Ethidimuron	115	8.83	108	3.28
Ethiofencarb	108	8.04	100	2.56
Ethiofencarb-sulfone	115	5.77	109	1.75

Ethiofencarb-sulfoxide	104	4.87	117	2.22
Ethion	98	14.27	91	5.91
Ethiprole	113	18.96	84	5.28
Ethirimol	92	6.30	92	2.13
Ethoprophos	88	8.86	78	6.05
Etoxazole	93	8.17	85	3.99
Etrimfos	89	3.79	80	3.90
Fenamidone	92	7.59	97	2.19
Fenamiphos	94	6.32	84	1.46
Fenamiphos-sulfone	76	14.16	79	4.33
Fenamiphos-sulfoxide	77	13.39	85	4.16
Fenarimol	100	13.71	99	7.68
Fenbuconazole	96	10.76	83	3.64
Fenfuram	108	7.26	96	1.19
Fenhexamid	106	13.17	97	4.07
Fenobucarb	78	7.53	88	2.11
Fenoxycarb	87	12.78	81	5.26
Fenpropimorph	117	7.63	108	3.39
Fensulfothion	85	14.68	86	3.79
Fensulfothion-oxon	98	12.00	93	5.95
Fensulfothion-sulfone	95	10.58	92	4.71
Fenthion-sulfoxid	81	9.28	87	4.26
Fenuron	69	5.07	94	3.40
Flamprop-isopropyl	114	11.47	99	5.02
Flamprop-methyl	92	4.31	95	3.03
Flazasulfuron	96	5.58	98	3.31
Flonicamid	104	19.09	93	3.02
Florasulam	78	12.16	94	4.80
Fluazuron	114	13.50	77	13.02

Flubendiamide	100	16.54	90	5.99
Flufenacet	118	7.76	118	3.50
Flufenoxuron	109	12.64	84	4.38
Fluometuron	91	6.71	97	3.58
Fluopicolide	107	11.33	96	3.22
Fluopyram	97	11.03	110	4.50
Fluoxastrobin	100	5.70	80	3.71
Fluquinconazole	85	16.16	93	5.91
Fluridone	102	4.57	91	3.47
Flusilazole	96	5.35	100	2.51
Flutolanil	101	5.14	97	2.89
Flutriafol	81	18.94	106	2.15
Fluxapyroxad	111	5.36	109	3.95
Fonofos	82	16.50	71	6.92
Foramsulfuron	103	3.67	100	2.55
Forchlorfenuron	109	5.93	100	4.42
Formetanate HCl	104	3.22	92	2.06
Fosthiazat	117	5.74	109	2.38
Fuberidazole	93	10.10	96	1.20
Furalaxyl	92	5.24	97	2.23
Furathiocarb	95	4.59	71	2.82
Halofenozide	86	17.42	102	6.21
Haloxyfop-2-ethoxyethyl	100	7.59	73	2.96
Haloxyfop-P-methyl	82	9.98	92	2.10
Heptenophos	81	12.24	83	4.10
Hexaconazole	110	5.69	105	2.89
Hexaflumuron	119	10.19	114	4.99
Hexazionon(Velpar)	109	3.07	102	2.30
Hydramethylnon	91	4.48	95	2.52

Hydroxycarbofuran	98	10.56	101	3.73
Imazalil	105	7.22	82	4.17
Imazaquin	85	17.99	98	3.49
Imidacloprid	85	20.00	102	4.53
Indoxacarb	82	17.75	72	3.74
Ipconazole	94	6.34	94	3.53
Iprobenfos	98	6.74	97	3.38
Iprovalicarb	101	4.35	97	3.52
Isocarbamide	106	8.32	96	3.12
Isofenphos	108	7.33	97	7.27
Isofenphos-methyl	90	11.69	80	5.39
Isoprocarb	95	9.50	87	2.98
Isoprothiolane	117	7.94	96	3.19
Isoproturon	103	5.36	98	1.82
Isoxaflutole	109	9.58	98	3.35
Isoxathion	100	8.76	97	4.45
Ivermectin	112	8.83	77	10.31
izoxaben	106	8.19	90	2.88
Kresoxim-methyl	108	16.50	84	2.66
Lenacil	93	11.93	97	3.28
Lufenuron	101	13.84	110	4.96
Malaoxon	104	9.02	102	3.44
Malathion	104	14.31	112	8.05
Mandipropamid	107	4.13	99	3.94
Mecarbam	92	7.71	83	5.08
Mefenacet	86	5.87	73	3.19
Mepanipyrim	93	14.78	82	2.88
Mephosfolan	98	2.27	101	1.22
Mepronil	102	4.22	97	3.22

Mesotrione	92	2.66	84	3.21
Metaflumizone	113	11.15	75	3.23
Metalaxyl	97	7.68	108	4.74
Metamitron	92	9.90	94	4.49
Metazachlor	101	8.92	98	1.54
Metconazole	107	9.02	105	2.83
Methabenzthiazuron	105	3.89	91	1.71
Methamidophos	97	7.66	104	3.50
Methidathion	97	17.98	97	4.62
Methiocarb	88	5.25	85	3.50
Methiocarb-sulfone	84	16.53	94	4.72
Methomyl	109	6.36	102	3.70
Methoprotryne	99	4.97	94	2.69
Methoxyfenozide	101	6.86	102	1.93
Metobromuron	99	12.34	96	3.90
Metolachlor	109	4.13	99	4.49
Metolcarb	111	6.67	92	3.62
Metosulam	88	8.70	98	3.38
Metoxuron	92	8.48	92	3.13
Metrafenon	97	7.18	78	3.29
Metsulfuron-methyl	99	6.68	101	6.68
Mevinphos-Isomer	108	15.30	109	3.36
Mexacarbate	102	3.95	93	2.24
Molinate	107	11.11	96	8.36
Monocrotophos	75	7.33	82	3.03
Monolinuron	111	4.67	97	2.34
Moxidectin	109	14.79	92	4.16
Myclobutanil	101	6.16	90	3.09
Napropamide	110	5.82	106	3.92

Neburon	91	5.91	95	3.57
Nitenpyram	106	9.53	94	4.11
Nitralin	110	12.66	93	4.98
Norflurazon	96	7.43	96	4.26
Novaluron	110	6.68	110	4.58
Nuarimol	101	11.02	99	4.01
Omethoate	95	6.30	98	2.14
Oxadiazon	92	13.46	86	2.95
Oxadixyl	110	13.26	100	4.92
Oxamyl	113	6.28	98	2.45
Oxamyl-oxime	92	9.44	100	2.33
Oxydemeton-methyl	99	3.68	104	0.95
Paclobutrazol	91	8.35	95	2.90
Paraoxon	72	2.67	84	5.54
Paraoxon-ethyl	80	5.10	84	4.01
Penconazole	88	8.44	96	5.28
Penthiopyrad	106	5.19	107	2.54
Pethoxamid	96	4.21	86	3.37
Phenmedipham	81	6.68	79	5.69
Phenthoate	104	10.86	87	5.16
Phorate-sulfone	104	19.54	98	6.20
Phorate-sulfoxide	120	9.70	106	1.80
Phosalone	70	12.68	96	2.49
Phosmet	87	11.60	70	10.45
Phosphamidon	98	7.62	88	3.48
Phoxim	115	7.83	100	3.85
Picoxystrobin	103	7.72	91	2.13
Pinoxaden	102	3.01	96	2.53
Piperonyl-butoxide	100	7.53	93	3.49

Piperophos	103	3.50	83	2.11
Pirimicarb	98	4.61	95	2.16
Pirimicarb-desmethyl	98	5.81	92	2.74
Pirimiphos-ethyl	102	6.86	86	2.67
Pirimiphos-methyl	96	7.54	79	2.64
Pirimiszulfuron-metil	107	8.34	95	4.57
Prochloraz	115	5.83	93	2.30
Prochloraz-desimidazole-amino	92	6.45	100	6.94
Promecarb	93	10.06	86	2.95
Prometon	104	5.71	94	5.42
Prometryne	99	3.22	91	1.25
Propachlor	92	7.63	97	3.35
Propamocarb	100	4.86	98	1.06
Propanil	97	12.01	95	6.22
Propargite	108	4.36	108	3.44
Propetamphos	102	10.78	92	5.31
Propiconazole	84	10.54	97	2.94
Propoxur	116	3.64	107	2.74
Propyzamide	110	15.91	100	4.24
Prosulfocarb	92	4.50	83	2.30
Pymetrozine	98	3.99	98	3.15
Pyracarbolid	89	4.06	90	1.68
Pyraclostrobin	94	8.53	107	5.66
Pyraflufen-ethyl	86	9.94	83	6.28
Pyrazophos	89	9.53	91	4.00
Pyridafol	102	9.14	100	2.61
Pyridaphenthion	80	5.58	90	3.52
Pyrifenox	104	2.77	90	2.62
Pyrimethanil	90	9.12	83	3.81

Pyroquilon	98	11.53	87	4.31
Quinalphos	92	19.00	76	2.86
Sebuthylazin	97	8.37	95	2.94
Secbumeton	101	3.52	95	1.08
Silthiofam	98	6.32	90	2.81
Simazine	92	10.32	97	2.70
Simetryn	98	6.03	93	1.11
Spinetoram	118	10.87	102	2.98
Spiromesifen	84	13.66	85	6.04
Spirotetramat	109	4.76	98	3.89
Spiroxamine-Isomer	89	2.38	94	1.82
Sulfometuron-methyl	96	3.39	90	2.49
Sulfosulfuron	99	10.06	105	1.82
Tebuconazole	91	9.47	103	2.44
Tebufenozide	109	8.43	100	3.51
Tebufenpyrad	87	5.45	77	5.02
Tebutam	102	6.38	93	2.63
Tebuthiuron	93	4.12	92	1.65
Terbufos-sulfoxide	114	4.56	113	3.16
Terbumeton	96	2.22	94	2.01
Terbuthylazine-desethyl	88	15.63	93	1.42
Terbutryn	89	3.83	92	2.23
Tetrachlorvinphos	86	15.54	79	3.81
Tetraconazole	102	8.43	97	5.81
Thiabendazole	103	4.25	101	3.65
Thiacloprid	89	8.51	96	3.06
Thiamethoxam	100	7.73	103	2.37
Thidiazuron	109	6.55	106	4.64
Thifensulfuron-methyl	100	4.53	99	3.19

Thiodicarb	105	7.63	103	3.75
Thiofanox-sulfoxide	78	19.14	101	2.57
Thiophanate	114	6.49	84	4.87
Thiophanate-methyl	93	10.10	87	6.53
Tralkoxydim	97	12.28	77	4.84
Triadimefon	100	8.62	97	2.91
Triadimenol	107	12.20	99	6.73
Triasulforon	90	11.42	99	2.85
Triazophos	94	5.51	84	2.91
Tricyclazole	95	5.63	99	2.87
Trietazin	108	10.48	100	5.49
Trifloxystrobin	91	9.76	73	3.81
Triflumizole	89	4.20	99	3.18
Triflumuron	112	7.70	95	4.60
Triflusulfuron-methyl	90	6.23	94	1.93
Trimethylphenyl-methylcarbamate	86	10.69	92	11.97
Triticonazole	107	7.54	102	5.03
Vamidothion	109	2.98	104	1.72
Vamidothion-sulfoxide	113	6.68	101	1.29
Zoxamide	107	3.99	96	3.48
(**b**)				
**Compound**	**Recovery% (10 ng/L)**	**RSD% (10 ng/L)**	**Recovery% (100 ng/L)**	**RSD% (100 ng/L)**
(Monceren)Pencycuron	100	3.81	79	1.78
3-Hydroxycarbofuran	94	7.41	89	2.10
Acephate	65	19.70	116	1.45
Acetamiprid	94	4.47	105	2.36
Acibenzolar-S-methyl	87	18.65	81	8.50
Aldicarb	110	14.82	100	6.71
Aldicarb-sulfone	104	7.16	101	3.26

Aldicarb-sulfoxide	102	11.85	84	3.47
Ametoctradin	100	3.56	95	3.25
Ametryn	92	5.57	94	1.52
Amidosulfuron	108	6.56	98	1.56
Aminocarb	90	6.37	99	1.37
Amitraz	114	10.77	90	2.51
Anilofos	95	6.90	82	4.36
Atrazine	98	9.63	102	4.23
Atrazine-desethyl	87	11.34	100	3.14
Atrazine-desisopropyl	107	8.28	95	3.87
Azaconazole	89	9.06	94	4.76
Azamethiphos	86	7.78	98	3.37
Azinphos-ethyl	103	14.07	88	6.72
Azinphos-methyl	94	11.55	90	11.77
Aziprotryne	83	11.90	94	3.20
Azoxystrobin	89	8.68	89	1.78
Benalaxyl	99	4.82	96	4.00
Bendiocarb	102	9.15	95	2.13
Benflutamid	84	9.78	90	3.14
Bensulfuron-metil	103	7.57	91	2.81
Benthiavalicarb-isopropyl	90	11.47	92	2.35
Benzolyprop-ethyl	103	4.24	97	3.13
Benzoximate	84	4.55	84	3.63
Benzthiazuron	97	7.97	78	4.31
Bifenazate	90	12.25	109	5.08
Bitertanol	100	8.46	92	2.80
Boscalid	97	10.35	93	10.54
Bromucanozole-Isomer	102	15.18	84	4.55
Bupirimate	95	5.38	94	2.73

Buprofezin	85	9.43	70	3.94
Butafenacil	106	4.74	95	3.22
Butocarboxim-sulfoxide	77	14.96	87	2.24
Butoxycarboxim	88	14.04	68	2.17
Buturon	96	16.82	96	3.37
Cadusafos	89	5.92	95	1.75
Carbaryl	84	12.42	66	3.53
Carbendazim	93	7.98	100	3.49
Carbetamide	103	6.17	116	3.54
Carbofuran	97	3.39	108	1.62
Carboxin	97	5.42	101	1.73
Carfentrazon	107	6.24	94	3.75
Carfentrazone-ethyl	101	13.79	61	3.25
Chlorantraniliprole	116	6.07	101	3.01
Chlorbromuron	71	15.37	86	4.81
Chlorfenvinphos	100	7.59	78	2.61
Chlorfluazuron	104	8.60	74	4.95
Chloridazon	104	12.22	105	2.85
Chlorotoluron	107	6.00	105	3.67
Chloroxuron	112	6.40	95	3.06
Chlorpyrifos	117	15.61	107	8.78
Chlorpyrifos-methyl	107	18.90	85	12.47
Chlorsulfuron	91	13.22	96	2.44
Chromafenozide	99	7.75	98	2.62
Cinosulfuron	100	5.65	95	1.87
Cirimidine	109	9.27	89	3.62
Clethodim-Isomer	99	13.17	83	4.30
Clomazone	100	5.19	90	1.60
Cloquintocet-mexyl	108	5.74	78	3.14

Clothianidin	105	13.49	110	4.41
Coumaphos	97	9.27	77	2.29
Crufomat(Ruelene)	88	11.42	83	2.96
Cyanazine	117	8.50	98	4.24
Cyazofamid	105	6.79	83	3.79
Cybutrine	100	4.29	99	1.71
Cyflufenamid	102	7.16	95	2.13
Cymiazole	117	3.56	95	4.86
Cymoxanil	90	19.12	93	4.77
Cyproconazole-Isomer	79	15.46	97	3.87
Cyprodinil	110	10.89	68	4.99
Cyromazine	94	11.24	102	2.45
Demeton-S	106	13.38	120	7.25
Demeton-S-methylsulphone	101	3.22	99	1.19
Demeton-S-sulfoxide	101	5.37	100	2.64
Desmedipham	114	5.66	105	2.70
Desmetryn	111	5.86	92	2.01
Diazinon	90	7.66	104	4.09
Diclobutrazol	95	8.42	99	5.30
Dicrotophos	94	14.33	69	1.61
Diethofencarb	98	7.29	106	2.61
Difenoconazole-Isomer	111	6.70	82	2.66
Difenoxuron	96	9.21	96	2.43
Diflubenzuron	104	10.05	85	2.57
Diflufenican	95	5.86	80	2.80
Dimefox	95	7.52	77	2.63
Dimefuron	76	18.20	72	2.11
Dimethachlor	104	11.47	88	5.44
Dimethenamid	109	4.00	94	2.05

Dimethirimol	101	4.92	92	2.96
Dimethoate	107	4.19	100	2.04
Dimethomorph-Isomer	106	15.90	85	4.70
Dimoxystrobin	113	4.19	99	3.35
Diniconazole	111	6.04	95	4.34
Dioxacarb	101	8.21	109	2.11
Diphenamid	104	4.12	91	1.98
Dipropetryn	95	7.77	94	3.04
Diuron	92	6.50	95	2.22
Dodemorph	119	19.16	89	3.62
Emamectin-benzoate b1a	102	9.40	97	2.65
Epoxiconazole	100	5.55	87	4.44
Etaconazole-Isomer	95	7.06	96	3.64
Ethidimuron	111	6.83	105	2.51
Ethiofencarb	102	7.78	104	2.66
Ethiofencarb-sulfone	117	7.80	112	2.26
Ethiofencarb-sulfoxide	97	10.55	116	3.46
Ethion	90	16.44	95	7.35
Ethiprole	118	4.91	70	3.09
Ethirimol	100	6.24	91	1.87
Ethoprophos	95	7.83	80	3.50
Etoxazole	95	6.79	91	2.15
Etrimfos	107	9.04	79	2.90
Fenamidone	93	7.13	98	3.27
Fenamiphos	105	6.72	79	4.88
Fenamiphos-sulfone	94	11.93	67	5.90
Fenamiphos-sulfoxide	96	16.90	74	6.79
Fenarimol	106	11.29	89	5.59
Fenbuconazole	99	12.72	80	2.26

Fenfuram	109	8.14	97	2.70
Fenhexamid	113	12.98	97	3.32
Fenobucarb	89	11.61	83	3.52
Fenoxycarb	92	10.83	80	4.94
Fenpropimorph	111	9.39	105	5.31
Fensulfothion	93	12.89	75	4.14
Fensulfothion-oxon	107	10.59	79	2.88
Fensulfothion-sulfone	101	7.92	78	4.42
Fenthion-sulfoxid	100	11.01	79	3.39
Fenuron	90	16.96	100	2.60
Flamprop-isopropyl	117	9.62	101	3.01
Flamprop-methyl	101	14.04	100	5.40
Flazasulfuron	101	2.39	96	3.06
Flonicamid	102	6.77	77	3.35
Florasulam	78	13.91	89	1.77
Fluazuron	107	8.05	75	4.82
Flubendiamide	113	13.62	86	12.05
Flufenacet	110	6.74	104	6.28
Flufenoxuron	96	12.03	95	3.92
Fluometuron	97	7.79	95	2.41
Fluopicolide	116	8.56	95	2.78
Fluopyram	97	11.53	112	8.06
Fluoxastrobin	101	8.04	77	2.53
Fluquinconazole	99	20.00	109	7.22
Fluridone	108	4.55	91	2.56
Flusilazole	102	7.41	97	2.88
Flutolanil	104	5.85	94	2.17
Flutriafol	91	16.55	102	4.10
Fluxapyroxad	102	7.14	109	3.29

Fonofos	104	12.62	75	6.54
Foramsulfuron	104	3.87	97	1.83
Forchlorfenuron	108	6.34	102	2.50
Formetanate HCl	109	4.78	97	2.19
Fosthiazat	112	7.95	113	1.93
Fuberidazole	100	7.58	97	1.53
Furalaxyl	94	7.20	97	2.05
Furathiocarb	101	5.30	72	2.45
Halofenozide	97	19.76	99	9.03
Haloxyfop-2-ethoxyethyl	104	6.05	76	3.10
Haloxyfop-P-methyl	92	11.34	89	1.81
Heptenophos	98	11.19	76	9.26
Hexaconazole	113	10.20	100	3.49
Hexaflumuron	108	16.71	88	9.13
Hexazionon(Velpar)	101	7.32	107	2.86
Hydramethylnon	102	6.26	97	2.69
Hydroxycarbofuran	99	10.79	98	2.81
Imazalil	109	9.14	83	4.07
Imazaquin	92	13.33	96	4.04
Imidacloprid	80	14.90	100	3.78
Indoxacarb	90	11.27	73	5.02
Ipconazole	89	4.34	96	2.12
Iprobenfos	100	6.59	99	2.20
Iprovalicarb	103	8.06	97	3.81
Isocarbamide	101	11.82	102	3.21
Isofenphos	106	10.41	100	5.66
Isofenphos-methyl	93	16.33	88	8.19
Isoprocarb	98	10.79	82	1.89
Isoprothiolane	113	8.76	94	1.84

Isoproturon	105	5.55	97	1.72
Isoxaflutole	104	10.70	78	5.60
Isoxathion	111	14.36	99	4.59
Ivermectin	105	10.80	85	8.81
izoxaben	105	6.84	96	2.64
Kresoxim-methyl	104	18.49	90	6.60
Lenacil	93	8.57	90	2.26
Lufenuron	83	13.76	98	5.35
Malaoxon	107	3.13	102	3.99
Malathion	101	16.77	109	11.23
Mandipropamid	110	6.32	98	4.64
Mecarbam	95	11.13	94	4.67
Mefenacet	97	4.42	73	4.37
Mepanipyrim	107	15.09	80	3.93
Mephosfolan	97	4.10	101	3.37
Mepronil	107	2.14	96	1.80
Mesotrione	104	8.92	70	2.19
Metaflumizone	105	16.87	77	5.75
Metalaxyl	102	8.51	110	7.27
Metamitron	105	16.07	93	3.76
Metazachlor	109	6.03	104	3.94
Metconazole	107	3.71	101	2.75
Methabenzthiazuron	112	3.53	91	1.87
Methamidophos	102	9.66	106	2.72
Methidathion	85	19.55	100	3.52
Methiocarb	95	8.91	78	2.88
Methiocarb-sulfone	98	12.48	81	3.99
Methomyl	101	9.83	110	3.06
Methoprotryne	106	7.72	93	2.56

Methoxyfenozide	109	2.96	99	5.44
Metobromuron	113	11.29	92	2.58
Metolachlor	108	4.72	96	3.40
Metolcarb	120	7.30	88	6.37
Metosulam	91	9.16	97	4.83
Metoxuron	100	4.99	93	1.81
Metrafenon	103	6.12	76	2.96
Metsulfuron-methyl	100	5.38	100	5.38
Mevinphos-Isomer	112	11.34	114	3.71
Mexacarbate	107	4.33	93	2.00
Molinate	93	16.79	93	6.27
Monocrotophos	93	12.24	70	2.32
Monolinuron	117	8.79	96	2.90
Moxidectin	113	8.73	96	4.69
Myclobutanil	107	6.61	90	4.33
Napropamide	105	6.57	105	2.94
Neburon	101	5.10	94	2.62
Nitenpyram	102	8.57	98	2.97
Nitralin	108	10.72	85	4.64
Norflurazon	104	5.78	96	5.44
Novaluron	100	8.62	103	5.22
Nuarimol	108	7.71	94	5.80
Omethoate	88	3.24	103	2.14
Oxadiazon	83	18.74	72	3.31
Oxadixyl	113	10.53	95	3.64
Oxamyl	104	9.31	103	2.63
Oxamyl-oxime	92	4.33	99	3.22
Oxydemeton-methyl	93	2.70	107	0.90
Paclobutrazol	98	5.48	95	3.06

Paraoxon	87	9.57	70	3.52
Paraoxon-ethyl	94	12.43	70	2.32
Penconazole	91	13.21	93	2.51
Penthiopyrad	103	3.61	107	4.13
Pethoxamid	104	6.88	87	1.53
Phenmedipham	93	18.72	60	3.25
Phenthoate	98	15.17	93	3.48
Phorate-sulfone	98	17.07	90	5.48
Phorate-sulfoxide	113	16.07	110	3.34
Phosalone	80	17.43	89	2.31
Phosmet	95	10.12	97	7.05
Phosphamidon	106	5.89	79	2.23
Phoxim	111	9.05	100	3.86
Picoxystrobin	105	6.32	92	2.70
Pinoxaden	106	2.72	97	3.04
Piperonyl-butoxide	99	5.45	94	3.13
Piperophos	107	3.09	83	2.58
Pirimicarb	104	4.70	94	2.45
Pirimicarb-desmethyl	103	3.19	98	2.09
Pirimiphos-ethyl	106	5.56	86	2.51
Pirimiphos-methyl	102	5.86	79	3.52
Pirimiszulfuron-metil	108	6.44	96	3.75
Prochloraz	113	5.01	95	2.58
Prochloraz-desimidazole-amino	90	7.57	100	4.57
Promecarb	102	5.03	82	2.01
Prometon	105	3.44	92	1.12
Prometryne	106	2.83	92	1.55
Propachlor	92	6.82	96	2.58
Propamocarb	101	3.41	97	2.43

Propanil	88	9.58	81	6.28
Propargite	101	4.94	120	3.95
Propetamphos	105	10.92	85	3.92
Propiconazole	96	10.62	93	3.07
Propoxur	108	8.66	112	1.42
Propyzamide	114	9.17	96	4.94
Prosulfocarb	93	6.60	88	3.15
Pymetrozine	99	3.49	98	1.45
Pyracarbolid	92	6.41	93	3.33
Pyraclostrobin	96	6.71	108	5.85
Pyraflufen-ethyl	96	9.30	77	3.39
Pyrazophos	95	5.96	95	3.83
Pyridafol	111	6.96	96	2.51
Pyridaphenthion	82	7.36	91	2.59
Pyrifenox	105	5.70	90	2.79
Pyrimethanil	96	10.59	93	8.05
Pyroquilon	91	8.57	83	4.50
Quinalphos	94	19.25	79	2.16
Sebuthylazin	102	6.36	93	4.47
Secbumeton	106	3.39	93	2.09
Silthiofam	103	6.80	92	3.52
Simazine	106	14.91	96	3.98
Simetryn	102	4.15	93	2.68
Spinetoram	95	10.77	110	4.44
Spiromesifen	100	13.57	78	5.95
Spirotetramat	118	7.72	99	4.69
Spiroxamine-Isomer	97	4.70	97	2.51
Sulfometuron-methyl	102	3.86	90	3.06
Sulfosulfuron	93	11.97	104	3.31

Tebuconazole	91	7.04	103	2.88
Tebufenozide	104	5.73	100	4.79
Tebufenpyrad	95	6.48	77	4.67
Tebutam	105	6.69	92	1.45
Tebuthiuron	100	4.34	95	2.38
Terbufos-sulfoxide	107	13.32	114	2.49
Terbumeton	101	4.15	94	1.46
Terbuthylazine-desethyl	90	9.12	92	2.10
Terbutryn	97	3.52	95	2.10
Tetrachlorvinphos	110	14.09	70	6.60
Tetraconazole	105	9.83	90	2.80
Thiabendazole	103	3.23	100	2.29
Thiacloprid	99	5.14	92	2.32
Thiamethoxam	101	4.89	102	2.43
Thidiazuron	103	9.31	101	3.57
Thifensulfuron-methyl	100	4.04	97	2.40
Thiodicarb	102	7.35	101	3.57
Thiofanox-sulfoxide	90	13.33	104	3.65
Thiophanate	114	6.21	89	3.71
Thiophanate-methyl	101	10.28	96	3.59
Tralkoxydim	102	12.55	78	3.52
Triadimefon	106	7.19	92	3.29
Triadimenol	112	8.64	99	4.26
Triasulforon	88	14.47	96	3.04
Triazophos	103	3.74	86	3.16
Tricyclazole	99	4.66	97	3.52
Trietazin	114	5.77	94	4.19
Trifloxystrobin	100	5.06	74	3.41
Triflumizole	91	6.12	99	3.69

Triflumuron	103	7.31	94	3.36
Triflusulfuron-methyl	97	3.74	94	3.13
Trimethylphenyl-methylcarbamate	99	9.72	85	12.63
Triticonazole	112	4.55	101	3.20
Vamidothion	104	2.92	107	2.45
Vamidothion-sulfoxide	103	7.12	102	2.25
Zoxamide	111	3.94	96	4.24

Table S3: Recovery% and precision (RSD%) in groundwater at 10 ng/L spiking level using automated SPE enrichment.

**Compounds**	**Recovery% (10 ng/L)**	**RSD% (10 ng/L)**
(Monceren)Pencycuron	103.6	9.0
3-Hydroxycarbofuran	106.0	5.1
Acephate	119.6	14.7
Acetamiprid	93.6	2.8
Acibenzolar-S-methyl	95.6	15.6
Aldicarb	107.2	19.1
Aldicarb-sulfone	114.3	6.2
Aldicarb-sulfoxide	117.5	5.4

Ametoctradin	96.6	6.7
Ametryn	105.6	7.2
Amidosulfuron	95.7	5.4
Aminocarb	106.3	9.9
Amitraz	117.8	18.6
Anilofos	106.3	7.1
Atrazine	106.9	7.6
Atrazine-desethyl	119.9	9.3
Atrazine-desisopropyl	96.4	15.2
Azaconazole	120.0	7.9
Azamethiphos	115.0	8.3
Azinphos-ethyl	117.1	12.3
Azinphos-methyl	118.2	13.0
Aziprotryne	100.4	8.6
Azoxystrobin	119.8	11.0
Benalaxyl	105.3	7.6
Bendiocarb	108.8	8.6
Benflutamid	104.9	8.0
Bensulfuron-metil	114.3	8.1
Benthiavalicarb-isopropyl	106.0	10.9
Benzolyprop-ethyl	105.3	7.7
Benzoximate	107.5	11.7
Benzthiazuron	102.3	9.9
Bifenazate	118.2	8.4
Bitertanol	102.0	10.5
Boscalid	112.9	9.2
Bromucanozole-Isomer	110.9	13.3
Bupirimate	106.0	5.6
Buprofezin	93.1	10.2

Butafenacil	111.6	6.4
Butocarboxim-sulfoxide	117.5	10.7
Butoxycarboxim	116.0	5.8
Buturon	116.7	7.8
Cadusafos	96.3	6.3
Carbaryl	118.0	5.7
Carbendazim	110.2	4.2
Carbetamide	115.4	6.3
Carbofuran	109.8	7.4
Carboxin	112.9	8.8
Carfentrazon	108.5	8.0
Carfentrazone-ethyl	110.1	10.0
Chlorantraniliprole	112.4	8.7
Chlorbromuron	105.9	8.7
Chlorfenvinphos	105.9	6.1
Chlorfluazuron	103.4	16.1
Chloridazon	118.0	4.5
Chlorotoluron	93.6	3.2
Chloroxuron	114.7	7.9
Chlorpyrifos	82.5	19.9
Chlorsulfuron	119.9	6.3
Chromafenozide	109.9	7.0
Cinosulfuron	102.4	6.9
Cirimidine	94.7	9.2
Clethodim-Isomer	119.3	19.7
Clomazone	102.9	8.8
Cloquintocet-mexyl	92.6	9.4
Clothianidin	118.8	10.5
Coumaphos	103.6	13.3

Crufomat(Ruelene)	107.2	5.8
Cyanazine	116.2	6.4
Cyazofamid	104.9	6.6
Cybutrine	100.9	11.6
Cyflufenamid	100.2	10.1
Cymiazole	75.5	17.1
Cymoxanil	111.6	7.4
Cyproconazole-Isomer	114.0	10.7
Cyprodinil	119.9	19.0
Cyromazine	119.8	15.5
Demeton-S	108.7	9.9
Demeton-S-methylsulphone	95.2	5.6
Demeton-S-sulfoxide	100.7	7.1
Desmedipham	108.5	6.1
Desmetryn	107.1	6.7
Diazinon	96.0	5.3
Diclobutrazol	104.5	11.4
Dicrotophos	97.4	6.7
Diethofencarb	106.7	7.7
Difenoconazole-Isomer	98.9	13.3
Difenoxuron	118.6	8.6
Diflubenzuron	111.5	9.0
Diflufenican	104.9	14.2
Dimefox	120.0	9.8
Dimefuron	114.2	8.6
Dimethachlor	104.0	8.3
Dimethenamid	111.1	8.8
Dimethirimol	117.8	7.8
Dimethoate	99.6	4.4

Dimethomorph-Isomer	104.5	12.7
Dimoxystrobin	107.0	8.0
Diniconazole	110.8	12.7
Dioxacarb	105.5	5.7
Diphenamid	108.6	7.9
Dipropetryn	105.7	7.7
Diuron	112.3	5.2
Dodemorph	91.3	5.0
Emamectin-benzoate b1a	91.4	12.7
Epoxiconazole	110.5	6.6
Etaconazole-Isomer	109.4	11.5
Ethidimuron	116.0	6.8
Ethiofencarb	118.8	13.7
Ethiofencarb-sulfone	97.6	6.4
Ethiofencarb-sulfoxide	98.6	7.6
Ethion	83.7	17.7
Ethiprole	113.5	7.7
Ethirimol	112.2	7.2
Ethoprophos	101.6	10.0
Etoxazole	93.0	14.1
Etrimfos	91.0	6.2
Fenamidone	119.9	5.2
Fenamiphos	119.9	7.9
Fenamiphos-sulfone	117.6	7.0
Fenamiphos-sulfoxide	104.1	7.8
Fenarimol	112.3	7.6
Fenbuconazole	106.0	9.0
Fenfuram	110.1	10.1
Fenhexamid	111.0	7.3

Fenobucarb	101.4	8.1
Fenoxycarb	108.2	10.6
Fenpropimorph	92.6	6.2
Fensulfothion	112.6	9.0
Fensulfothion-oxon	109.1	6.4
Fensulfothion-sulfone	112.1	5.7
Fenthion-sulfoxid	112.3	7.5
Fenuron	102.2	6.4
Flamprop-isopropyl	106.8	10.5
Flamprop-methyl	110.0	9.7
Flazasulfuron	108.9	5.1
Flonicamid	108.6	9.0
Florasulam	118.4	5.7
Fluazuron	91.2	17.2
Flubendiamide	117.1	19.9
Flufenacet	108.0	8.4
Flufenoxuron	94.9	14.4
Fluometuron	108.8	7.4
Fluopicolide	108.3	7.5
Fluopyram	106.7	8.1
Fluoxastrobin	107.6	9.7
Fluquinconazole	97.2	8.7
Fluridone	105.2	7.9
Flusilazole	107.2	7.1
Flutolanil	112.2	4.8
Flutriafol	118.3	8.3
Fluxapyroxad	113.5	6.7
Fonofos	96.2	9.1
Foramsulfuron	104.4	6.2

Forchlorfenuron	116.6	5.7
Formetanate HCl	119.9	13.8
Fosthiazat	105.4	8.8
Fuberidazole	112.9	6.7
Furalaxyl	112.0	8.0
Furathiocarb	93.5	10.0
Halofenozide	118.3	16.1
Haloxyfop-2-ethoxyethyl	91.5	8.6
Haloxyfop-P-methyl	100.7	7.7
Heptenophos	105.3	9.9
Hexaconazole	103.0	8.3
Hexaflumuron	105.7	18.3
Hexazionon(Velpar)	109.7	6.4
Hydramethylnon	101.3	18.2
Hydroxycarbofuran	106.7	5.4
Imazalil	100.7	6.5
Imazaquin	99.1	6.3
Imidacloprid	98.7	3.9
Indoxacarb	95.3	11.3
Ipconazole	96.7	9.6
Iprobenfos	98.9	8.7
Iprovalicarb	107.2	7.8
Isocarbamide	118.0	16.0
Isofenphos	114.5	11.4
Isofenphos-methyl	115.6	19.7
Isoprocarb	102.5	10.0
Isoprothiolane	109.1	7.6
Isoproturon	110.5	7.6
Isoxaflutole	118.1	10.7

Isoxathion	99.3	13.2
Ivermectin	96.4	16.2
izoxaben	101.9	6.4
Kresoxim-methyl	109.3	9.8
Lufenuron	98.0	10.2
Malaoxon	109.4	9.1
Malathion	92.3	10.9
Mandipropamid	106.8	7.9
Mecarbam	104.2	12.5
Mefenacet	108.3	10.2
Mepanipyrim	108.6	11.7
Mephosfolan	104.2	8.4
Mepronil	111.4	7.4
Mesotrione	90.3	4.6
Metaflumizone	97.2	14.3
Metalaxyl	113.5	9.7
Metamitron	109.5	6.5
Metazachlor	111.1	8.1
Metconazole	106.9	9.1
Methabenzthiazuron	110.0	8.0
Methamidophos	118.5	18.0
Methidathion	109.7	8.1
Methiocarb	109.3	7.0
Methiocarb-sulfone	115.3	5.7
Methomyl	120.0	7.9
Methoprotryne	108.8	6.1
Methoxyfenozide	114.6	5.8
Metobromuron	104.7	7.5
Metolachlor	103.8	8.3

Metolcarb	104.3	15.9
Metosulam	110.2	6.3
Metoxuron	113.4	8.1
Metrafenon	102.2	8.4
Metsulfuron-methyl	105.9	7.2
Mevinphos-Isomer	98.6	11.9
Mexacarbate	104.6	8.3
Molinate	102.2	19.9
Monocrotophos	108.9	5.5
Monolinuron	102.9	7.4
Moxidectin	100.7	19.7
Myclobutanil	107.3	7.5
Napropamide	92.0	8.0
Neburon	108.2	6.5
Nitenpyram	90.5	6.1
Nitralin	105.9	14.4
Norflurazon	116.9	8.6
Novaluron	99.1	11.4
Nuarimol	108.5	8.9
Omethoate	117.5	6.3
Oxadiazon	92.2	8.8
Oxadixyl	108.0	8.2
Oxamyl	104.0	8.9
Oxamyl-oxime	119.5	19.8
Oxydemeton-methyl	105.9	6.7
Paclobutrazol	113.0	8.3
Paraoxon	115.2	6.7
Paraoxon-ethyl	118.0	8.4
Penconazole	104.9	5.5

Penthiopyrad	112.5	7.9
Pethoxamid	103.1	8.6
Phenmedipham	113.3	5.9
Phenthoate	113.0	9.0
Phorate-sulfone	113.2	11.3
Phorate-sulfoxide	112.6	7.5
Phosalone	104.2	15.1
Phosmet	115.8	10.9
Phosphamidon	98.7	6.1
Phoxim	96.6	8.8
Picoxystrobin	102.9	6.6
Pinoxaden	102.8	7.3
Piperonyl-butoxide	98.6	14.9
Piperophos	100.3	8.9
Pirimicarb	105.0	8.6
Pirimicarb-desmethyl	109.1	4.9
Pirimiphos-ethyl	89.7	7.4
Pirimiphos-methyl	93.3	8.1
Pirimiszulfuron-metil	117.9	10.8
Prochloraz	100.2	9.3
Prochloraz-desimidazole-amino	109.9	10.4
Promecarb	101.6	8.1
Prometon	106.8	7.1
Prometryne	104.8	5.9
Propachlor	104.9	9.2
Propamocarb	103.8	18.7
Propanil	107.3	7.6
Propargite	86.4	17.9
Propetamphos	100.7	10.2

Propiconazole	118.4	11.6
Propoxur	110.5	7.2
Propyzamide	108.5	8.6
Prosulfocarb	91.4	9.7
Pymetrozine	117.8	5.3
Pyracarbolid	108.4	6.4
Pyraclostrobin	101.7	6.6
Pyraflufen-ethyl	109.2	7.8
Pyrazophos	99.0	14.5
Pyridafol	100.0	4.2
Pyridaphenthion	110.3	7.4
Pyrifenox	101.8	7.5
Pyrimethanil	92.3	7.2
Pyroquilon	111.8	6.3
Quinalphos	99.8	9.6
Sebuthylazin	116.3	5.8
Secbumeton	110.4	6.4
Silthiofam	105.6	5.9
Simazine	114.6	8.0
Simetryn	109.6	6.6
Spinetoram	93.1	11.6
Spiromesifen	97.2	17.5
Spirotetramat	119.2	9.1
Spiroxamine-Isomer	97.0	8.0
Sulfometuron-methyl	103.8	9.7
Sulfosulfuron	116.8	9.0
Tebuconazole	108.5	6.3
Tebufenozide	101.8	8.6
Tebufenpyrad	96.1	8.4

Tebutam	98.9	12.9
Tebuthiuron	112.7	5.7
Terbufos-sulfoxide	110.8	8.6
Terbumeton	108.4	7.5
Terbuthylazine-desethyl	118.6	6.6
Terbutryn	110.0	9.2
Tetrachlorvinphos	108.3	8.6
Tetraconazole	107.5	7.0
Thiabendazole	119.5	19.9
Thiacloprid	112.5	4.9
Thiamethoxam	118.8	18.8
Thidiazuron	119.9	7.9
Thifensulfuron-methyl	103.0	7.0
Thiodicarb	101.7	9.3
Thiofanox-sulfoxide	93.0	14.4
Thiophanate	115.6	8.9
Thiophanate-methyl	119.5	17.9
Tralkoxydim	110.5	10.2
Triadimefon	109.1	8.2
Triadimenol	116.5	11.6
Triasulforon	117.4	6.9
Triazophos	108.0	5.7
Tricyclazole	111.4	4.9
Trietazin	106.6	8.8
Trifloxystrobin	100.0	8.4
Triflumizole	101.9	8.7
Triflumuron	107.1	7.3
Triflusulfuron-methyl	107.9	9.3
Trimethylphenyl-methylcarbamate	119.5	16.4

Triticonazole	106.5	10.0
Vamidothion	97.9	7.5
Vamidothion-sulfoxide	97.2	6.7
Zoxamide	108.7	6.2

Table S4: Results of real sample analysis. Individual concentrations and sum concentrations including or excluding results under 10 ng/L. G: groundwater, nd: non-detected, S: surface water. Single analysis was carried out for each sample.

**Compounds**	**G1**	**S1**	**S2**	**S3**	**S4**	**S5**	**S6**	**S7**	**S8**	**S9**	**S10**	**S11**	**S12**	**S13**	**S14**	**S15**	**S16**	**S17**	**S18**	**S19**
(Monceren)Pencycuron	nd	nd	nd	nd	nd	nd	nd	nd	nd	nd	nd	nd	nd	nd	nd	nd	nd	nd	1.44	nd
3-Hydroxycarbofuran	nd	nd	nd	nd	nd	nd	nd	nd	nd	nd	nd	nd	nd	nd	nd	nd	nd	nd	nd	nd
Acephate	nd	nd	nd	nd	nd	nd	nd	nd	nd	nd	nd	nd	nd	nd	nd	nd	nd	nd	nd	nd
Acetamiprid	nd	nd	nd	20.50	nd	nd	nd	nd	nd	1.95	nd	3.67	nd	nd	nd	nd	nd	nd	1.46	nd

Acibenzolar-S-methyl	nd	nd	nd	nd	nd	nd	nd	nd	nd	nd	nd	nd	nd	nd	nd	nd	nd	nd	nd	nd
Aldicarb	nd	nd	nd	nd	nd	nd	nd	nd	nd	nd	nd	nd	nd	nd	nd	nd	nd	nd	nd	nd
Aldicarb-sulfone	nd	nd	nd	nd	nd	nd	nd	nd	nd	nd	nd	nd	nd	nd	nd	nd	nd	nd	nd	nd
Aldicarb-sulfoxide	nd	nd	nd	nd	nd	nd	nd	nd	nd	nd	nd	nd	nd	nd	nd	nd	nd	nd	nd	nd
Ametoctradin	nd	nd	nd	nd	nd	nd	nd	nd	nd	nd	nd	nd	nd	nd	nd	nd	nd	nd	nd	nd
Ametryn	nd	nd	nd	nd	nd	nd	nd	nd	nd	nd	nd	nd	nd	nd	nd	nd	nd	nd	1.62	nd
Amidosulfuron	nd	nd	nd	nd	nd	nd	nd	nd	nd	nd	nd	nd	nd	nd	nd	nd	nd	nd	1.36	nd
Aminocarb	nd	nd	nd	nd	nd	nd	nd	nd	nd	nd	nd	nd	nd	nd	nd	nd	nd	nd	1.93	nd
Amitraz	nd	nd	nd	nd	nd	nd	nd	nd	nd	nd	nd	nd	nd	nd	nd	nd	nd	nd	nd	nd
Anilofos	nd	nd	nd	nd	nd	nd	nd	nd	nd	nd	nd	nd	nd	nd	nd	nd	nd	nd	1.42	nd
Atrazine	nd	4.33	2.80	3.76	5.61	5.66	3.83	4.63	3.75	8.27	4.29	8.10	2.83	4.33	4.37	3.09	4.58	3.45	4.94	4.18
Atrazine-desethyl	nd	nd	nd	nd	nd	nd	nd	nd	nd	31.10	nd	nd	nd	nd	31.94	nd	nd	nd	nd	nd
Atrazine-desisopropyl	nd	nd	nd	nd	nd	nd	nd	nd	nd	nd	nd	nd	nd	nd	nd	nd	nd	nd	nd	nd
Azaconazole	nd	nd	nd	nd	nd	nd	nd	nd	nd	nd	nd	nd	nd	nd	nd	nd	nd	nd	1.38	
Azamethiphos	nd	nd	nd	nd	nd	nd	nd	nd	nd	nd	nd	nd	nd	nd	nd	nd	nd	nd	nd	nd
Azinphos-ethyl	nd	nd	nd	nd	nd	nd	nd	nd	nd	nd	nd	nd	nd	nd	nd	nd	nd	nd	nd	nd
Azinphos-methyl	nd	nd	nd	nd	nd	nd	nd	nd	nd	nd	nd	nd	nd	nd	nd	nd	nd	nd	1.03	nd
Aziprotryne	nd	nd	nd	nd	nd	nd	nd	nd	nd	nd	nd	nd	nd	nd	nd	nd	nd	nd	nd	nd
Azoxystrobin	nd	nd	nd	5.67	nd	11.42	1.07	3.50	1.30	3.90	3.94	10.45	2.16	2.08	1.43	1.07	1.59	1.84	2.78	1.53

Benalaxyl	nd	nd	nd	nd	nd	nd	nd	nd	nd	nd	nd	nd	nd	nd	nd	nd	nd	nd	1.75	nd
Bendiocarb	nd	nd	nd	nd	nd	nd	nd	nd	nd	nd	nd	nd	nd	nd	nd	nd	nd	nd	1.84	nd
Benflutamid	nd	nd	nd	nd	nd	nd	nd	nd	nd	nd	nd	nd	nd	nd	nd	nd	nd	nd	nd	nd
Bensulfuron-metil	nd	nd	nd	nd	nd	nd	nd	nd	nd	nd	nd	nd	nd	nd	nd	nd	nd	nd	1.41	nd
Benthiavalicarb-isopropyl	nd	nd	nd	nd	nd	nd	nd	nd	nd	nd	nd	nd	nd	nd	nd	nd	nd	nd	1.81	nd
Benzolyprop-ethyl	nd	nd	nd	nd	nd	nd	nd	nd	nd	nd	nd	nd	nd	nd	nd	nd	nd	nd	1.81	nd
Benzoximate	nd	nd	nd	nd	nd	nd	nd	nd	nd	nd	nd	nd	nd	nd	nd	nd	nd	nd	nd	nd
Benzthiazuron	nd	nd	nd	nd	nd	nd	nd	nd	nd	nd	nd	nd	nd	nd	nd	nd	nd	nd	nd	nd
Bifenazate	nd	nd	nd	nd	nd	nd	nd	nd	nd	nd	nd	nd	nd	nd	nd	nd	nd	nd	nd	nd
Bitertanol	nd	nd	nd	nd	nd	nd	nd	nd	nd	nd	nd	nd	nd	nd	nd	nd	nd	nd	nd	nd
Boscalid	nd	nd	nd	15.32	nd	6.71	nd	nd	nd	6.15	nd	14.19	nd	nd	nd	nd	nd	nd	nd	nd
Bromucanozole-Isomer	nd	nd	nd	nd	nd	nd	nd	nd	nd	nd	nd	nd	nd	nd	nd	nd	nd	nd	nd	nd
Bupirimate	nd	nd	nd	nd	nd	nd	nd	nd	nd	nd	nd	nd	nd	nd	nd	nd	nd	nd	1.55	nd
Buprofezin	nd	nd	nd	nd	nd	nd	nd	nd	nd	nd	nd	nd	nd	nd	nd	nd	nd	nd	1.46	nd
Butafenacil	nd	nd	nd	nd	nd	nd	nd	nd	nd	nd	nd	nd	nd	nd	nd	nd	nd	nd	1.09	nd
Butocarboxim-sulfoxide	nd	nd	nd	nd	nd	nd	nd	nd	nd	nd	nd	nd	nd	nd	nd	nd	nd	nd	nd	nd
Butoxycarboxim	nd	nd	nd	nd	nd	nd	nd	nd	nd	nd	nd	nd	nd	nd	nd	nd	nd	nd	nd	nd
Buturon	nd	nd	nd	nd	nd	nd	nd	nd	nd	nd	nd	nd	nd	nd	nd	nd	nd	nd	nd	nd
Cadusafos	nd	nd	nd	nd	nd	nd	nd	nd	nd	nd	nd	nd	nd	nd	nd	nd	nd	nd	1.25	nd

Carbaryl	nd	nd	nd	nd	nd	nd	nd	nd	nd	nd	nd	nd	nd	nd	nd	nd	nd	nd	1.53	nd
Carbendazim	2.77	15.35	8.46	28.52	1.93	5.92	9.28	14.08	10.39	3.37	16.81	19.36	10.08	16.27	7.45	10.34	11.96	12.29	18.79	13.75
Carbetamide	nd	nd	nd	nd	nd	nd	nd	nd	nd	nd	nd	nd	nd	nd	nd	nd	nd	nd	1.13	nd
Carbofuran	nd	nd	nd	nd	nd	nd	nd	nd	nd	nd	nd	nd	nd	nd	nd	nd	nd	nd	1.55	nd
Carboxin	nd	nd	nd	nd	nd	nd	nd	nd	nd	nd	nd	nd	nd	nd	nd	nd	nd	nd	1.26	nd
Carfentrazon	nd	nd	nd	nd	nd	nd	nd	nd	nd	nd	nd	nd	nd	nd	nd	nd	nd	nd	1.23	nd
Carfentrazone-ethyl	nd	nd	nd	nd	nd	nd	nd	nd	nd	nd	nd	nd	nd	nd	nd	nd	nd	nd	1.66	nd
Chlorantraniliprole	nd	nd	nd	9.52	nd	3.26	nd	nd	nd	nd	nd	nd	nd	nd	nd	nd	nd	nd	1.38	nd
Chlorbromuron	nd	nd	nd	nd	nd	nd	nd	nd	nd	nd	nd	nd	nd	nd	nd	nd	nd	nd	nd	nd
Chlorfenvinphos	nd	nd	nd	nd	nd	nd	nd	nd	nd	nd	nd	nd	nd	nd	nd	nd	nd	nd	1.33	nd
Chlorfluazuron	nd	nd	nd	nd	nd	nd	nd	nd	nd	nd	nd	nd	nd	nd	nd	nd	nd	nd	nd	nd
Chloridazon	nd	nd	nd	nd	nd	nd	nd	nd	nd	nd	nd	nd	nd	nd	nd	nd	nd	nd	nd	nd
Chlorotoluron	nd	nd	nd	nd	nd	5.23	nd	nd	nd	nd	nd	nd	nd	nd	nd	nd	nd	nd	1.95	nd
Chloroxuron	nd	nd	nd	nd	nd	nd	nd	nd	nd	nd	nd	nd	nd	nd	nd	nd	nd	nd	nd	nd
Chlorpyrifos	nd	nd	nd	nd	nd	nd	nd	nd	nd	nd	nd	nd	nd	nd	nd	nd	nd	nd	nd	nd
Chlorsulfuron	nd	nd	nd	nd	nd	nd	nd	nd	nd	nd	nd	nd	nd	nd	nd	nd	nd	nd	nd	nd
Chromafenozide	nd	nd	nd	nd	nd	nd	nd	nd	nd	nd	nd	nd	nd	nd	nd	nd	nd	nd	1.34	nd
Cinosulfuron	nd	nd	nd	nd	nd	nd	nd	nd	nd	nd	nd	nd	nd	nd	nd	nd	nd	nd	1.22	nd
Cirimidine	nd	nd	nd	nd	nd	nd	nd	nd	nd	nd	nd	nd	nd	nd	nd	nd	nd	nd	nd	nd

Clethodim-Isomer	nd	nd	nd	nd	nd	nd	nd	nd	nd	nd	nd	nd	nd	nd	nd	nd	nd	nd	nd	nd
Clomazone	nd	nd	nd	nd	nd	nd	nd	nd	nd	nd	nd	nd	nd	nd	nd	nd	nd	nd	1.33	nd
Cloquintocet-mexyl	nd	nd	nd	nd	nd	nd	nd	nd	nd	nd	nd	nd	nd	nd	nd	nd	nd	nd	1.38	nd
Clothianidin	nd	nd	nd	nd	nd	4.00	nd	nd	nd	4.08	nd	8.71	nd	nd	nd	nd	nd	nd	nd	nd
Coumaphos	nd	nd	nd	nd	nd	nd	nd	nd	nd	nd	nd	nd	nd	nd	nd	nd	nd	nd	nd	nd
Crufomat(Ruelene)	nd	nd	nd	nd	nd	nd	nd	nd	nd	nd	nd	nd	nd	nd	nd	nd	nd	nd	nd	nd
Cyanazine	nd	nd	nd	nd	nd	nd	nd	nd	nd	nd	nd	nd	nd	nd	nd	nd	nd	nd	1.91	nd
Cyazofamid	nd	nd	nd	nd	nd	nd	nd	nd	nd	nd	nd	nd	nd	nd	nd	nd	nd	nd	nd	nd
Cybutrine	nd	nd	nd	nd	nd	nd	nd	nd	nd	nd	nd	nd	nd	nd	nd	nd	nd	nd	nd	nd
Cyflufenamid	nd	nd	nd	nd	nd	nd	nd	nd	nd	nd	nd	nd	nd	nd	nd	nd	nd	nd	nd	nd
Cymiazole	nd	nd	nd	nd	nd	nd	nd	nd	nd	nd	nd	nd	nd	nd	nd	nd	nd	nd	nd	nd
Cymoxanil	nd	nd	nd	nd	nd	nd	nd	nd	nd	nd	nd	nd	nd	nd	nd	nd	nd	nd	nd	nd
Cyproconazole-Isomer	nd	nd	nd	nd	nd	nd	nd	nd	nd	nd	nd	nd	nd	nd	nd	nd	nd	nd	nd	nd
Cyprodinil	nd	nd	nd	nd	nd	nd	nd	nd	nd	nd	nd	nd	nd	nd	nd	nd	nd	nd	nd	nd
Cyromazine	nd	nd	nd	nd	nd	nd	nd	nd	nd	nd	nd	nd	nd	nd	nd	nd	nd	nd	nd	nd
Demeton-S	nd	nd	nd	nd	nd	nd	nd	nd	nd	nd	nd	nd	nd	nd	nd	nd	nd	nd	nd	nd
Demeton-S-methylsulphone	nd	nd	nd	nd	nd	nd	nd	nd	nd	nd	nd	nd	nd	nd	nd	nd	nd	nd	1.42	nd
Demeton-S-sulfoxide	nd	nd	nd	nd	nd	nd	nd	nd	nd	nd	nd	nd	nd	nd	nd	nd	nd	nd	nd	nd
Desmedipham	nd	nd	nd	nd	nd	nd	nd	nd	nd	nd	nd	nd	nd	nd	nd	nd	nd	nd	1.39	nd

Desmetryn	nd	nd	nd	nd	nd	nd	nd	nd	nd	nd	nd	nd	nd	nd	nd	nd	nd	nd	1.56	nd
Diazinon	nd	nd	nd	nd	nd	nd	nd	nd	nd	nd	nd	nd	nd	nd	nd	nd	nd	nd	1.42	nd
Diclobutrazol	nd	nd	nd	nd	nd	nd	nd	nd	nd	nd	nd	nd	nd	nd	nd	nd	nd	nd	nd	nd
Dicrotophos	nd	nd	nd	nd	nd	nd	nd	nd	nd	nd	nd	nd	nd	nd	nd	nd	nd	nd	1.63	nd
Diethofencarb	nd	nd	nd	nd	nd	nd	nd	nd	nd	nd	nd	nd	nd	nd	nd	nd	nd	nd	1.38	nd
Difenoconazole-Isomer	nd	nd	nd	nd	nd	nd	nd	nd	nd	nd	nd	nd	nd	nd	nd	nd	1.71	nd	2.19	nd
Difenoxuron	nd	nd	nd	nd	nd	nd	nd	nd	nd	nd	nd	nd	nd	nd	nd	nd	nd	nd	1.65	nd
Diflubenzuron	nd	nd	nd	nd	nd	nd	nd	nd	nd	nd	nd	nd	nd	nd	nd	nd	nd	nd	1.02	nd
Diflufenican	nd	nd	nd	nd	nd	nd	nd	nd	nd	nd	nd	nd	nd	nd	nd	nd	nd	nd	nd	nd
Dimefox	nd	nd	nd	nd	nd	nd	nd	nd	nd	nd	nd	nd	nd	nd	nd	nd	nd	nd	nd	nd
Dimefuron	nd	nd	nd	nd	nd	nd	nd	nd	nd	nd	nd	nd	nd	nd	nd	nd	nd	nd	nd	nd
Dimethachlor	nd	nd	nd	nd	nd	nd	nd	nd	nd	nd	nd	nd	nd	nd	nd	nd	nd	nd	nd	nd
Dimethenamid	nd	nd	nd	nd	nd	nd	nd	nd	nd	nd	nd	1.50	nd	nd	1.24	nd	nd	nd	1.79	nd
Dimethirimol	nd	nd	nd	nd	nd	nd	nd	nd	nd	nd	nd	nd	nd	nd	nd	nd	nd	nd	1.52	nd
Dimethoate	nd	nd	nd	nd	nd	nd	nd	nd	nd	nd	nd	nd	nd	nd	nd	nd	nd	nd	1.42	nd
Dimethomorph-Isomer	nd	nd	nd	nd	4.04	nd	nd	nd	nd	nd	nd	nd	nd	nd	nd	nd	nd	nd	nd	nd
Dimoxystrobin	nd	nd	nd	nd	nd	nd	nd	nd	nd	nd	nd	nd	nd	nd	nd	nd	nd	nd	1.60	nd
Diniconazole	nd	nd	nd	nd	nd	nd	nd	nd	nd	nd	nd	nd	nd	nd	nd	nd	nd	nd	nd	nd
Dioxacarb	nd	nd	nd	nd	nd	nd	nd	nd	nd	nd	nd	nd	nd	nd	nd	nd	nd	nd	1.24	nd

Diphenamid	nd	nd	nd	nd	nd	nd	nd	nd	nd	nd	nd	nd	nd	nd	nd	nd	nd	nd	1.51	nd
Dipropetryn	nd	nd	nd	nd	nd	nd	nd	nd	nd	nd	nd	nd	nd	nd	nd	nd	nd	nd	nd	nd
Diuron	nd	nd	nd	5.40	nd	2.51	nd	nd	nd	3.16	nd	nd	nd	nd	nd	nd	nd	nd	nd	nd
Dodemorph	nd	nd	nd	nd	nd	nd	1.63	nd	nd	nd	nd	nd	nd	nd	nd	nd	nd	nd	3.12	2.01
Emamectin-benzoate b1a	nd	nd	nd	nd	nd	nd	nd	nd	nd	nd	nd	nd	nd	nd	nd	nd	nd	nd	nd	nd
Epoxiconazole	nd	nd	nd	nd	nd	nd	nd	nd	nd	nd	nd	8.40	nd	nd	nd	nd	nd	nd	nd	nd
Etaconazole-Isomer	nd	nd	nd	nd	nd	nd	nd	nd	nd	nd	nd	nd	nd	nd	nd	nd	nd	nd	nd	nd
Ethidimuron	nd	nd	nd	nd	nd	nd	nd	nd	nd	nd	nd	nd	nd	nd	nd	nd	nd	nd	1.50	nd
Ethiofencarb	nd	nd	nd	nd	nd	nd	nd	nd	nd	nd	nd	nd	nd	nd	nd	nd	nd	nd	nd	nd
Ethiofencarb-sulfone	nd	nd	nd	nd	nd	nd	nd	nd	nd	nd	nd	nd	nd	nd	nd	nd	nd	nd	1.74	nd
Ethiofencarb-sulfoxide	nd	nd	nd	nd	nd	nd	nd	nd	nd	nd	nd	nd	nd	nd	nd	nd	nd	nd	nd	nd
Ethion	nd	nd	nd	nd	nd	nd	nd	nd	nd	nd	nd	nd	nd	nd	nd	nd	nd	nd	nd	nd
Ethiprole	nd	nd	nd	nd	nd	nd	nd	nd	nd	nd	nd	nd	nd	nd	nd	nd	nd	nd	nd	nd
Ethirimol	nd	nd	nd	nd	nd	nd	nd	nd	nd	nd	nd	nd	nd	nd	nd	nd	nd	nd	nd	nd
Ethoprophos	nd	nd	nd	nd	nd	nd	nd	nd	nd	nd	nd	nd	nd	nd	nd	nd	nd	nd	nd	nd
Etoxazole	nd	nd	nd	nd	nd	nd	nd	nd	nd	nd	nd	nd	nd	nd	nd	nd	nd	nd	1.51	nd
Etrimfos	nd	nd	nd	nd	nd	nd	nd	nd	nd	nd	nd	nd	nd	nd	nd	nd	nd	nd	nd	nd
Fenamidone	nd	nd	nd	nd	nd	nd	nd	nd	nd	nd	nd	nd	nd	nd	nd	nd	nd	nd	1.48	nd
Fenamiphos	nd	nd	nd	nd	nd	nd	nd	nd	nd	nd	nd	nd	nd	nd	nd	nd	nd	nd	1.41	nd

Fenamiphos-sulfone	nd	nd	nd	nd	nd	nd	nd	nd	nd	nd	nd	nd	nd	nd	nd	nd	nd	nd	1.61	nd
Fenamiphos-sulfoxide	nd	nd	nd	nd	nd	nd	nd	nd	nd	nd	nd	nd	nd	nd	nd	nd	1.33	nd	1.63	nd
Fenarimol	nd	nd	nd	7.58	nd	nd	nd	nd	nd	nd	nd	nd	nd	nd	nd	nd	nd	nd	nd	nd
Fenbuconazole	nd	nd	nd	nd	nd	nd	nd	nd	nd	nd	nd	nd	nd	nd	nd	nd	nd	nd	nd	nd
Fenfuram	nd	nd	nd	nd	nd	nd	nd	nd	nd	nd	nd	nd	nd	nd	nd	nd	nd	nd	nd	nd
Fenhexamid	nd	nd	nd	36.32	nd	nd	nd	nd	nd	nd	nd	nd	nd	nd	nd	nd	nd	nd	nd	nd
Fenobucarb	nd	nd	nd	nd	nd	nd	nd	nd	nd	nd	nd	nd	nd	nd	nd	nd	nd	nd	1.33	nd
Fenoxycarb	nd	nd	nd	nd	nd	nd	nd	nd	nd	nd	nd	nd	nd	nd	nd	nd	nd	nd	nd	nd
Fenpropimorph	nd	nd	nd	nd	nd	nd	1.37	nd	nd	nd	nd	nd	nd	nd	nd	nd	nd	nd	2.65	1.53
Fensulfothion	nd	nd	nd	nd	nd	nd	nd	nd	nd	nd	nd	nd	nd	nd	nd	nd	nd	nd	1.54	nd
Fensulfothion-oxon	nd	nd	nd	nd	nd	nd	nd	nd	nd	nd	nd	nd	nd	nd	nd	nd	nd	nd	nd	nd
Fensulfothion-sulfone	nd	nd	nd	nd	nd	nd	nd	nd	nd	nd	nd	nd	nd	nd	nd	nd	nd	nd	nd	nd
Fenthion-sulfoxid	nd	nd	nd	nd	nd	nd	nd	nd	nd	nd	nd	nd	nd	nd	nd	nd	nd	nd	1.54	nd
Fenuron	nd	nd	nd	nd	nd	nd	nd	nd	nd	nd	nd	nd	nd	nd	nd	nd	nd	nd	nd	nd
Flamprop-isopropyl	nd	nd	nd	nd	nd	nd	nd	nd	nd	nd	nd	nd	nd	nd	nd	nd	nd	nd	nd	nd
Flamprop-methyl	nd	nd	nd	nd	nd	nd	nd	nd	nd	nd	nd	nd	nd	nd	nd	nd	nd	nd	nd	nd
Flazasulfuron	nd	nd	nd	nd	nd	nd	nd	nd	nd	nd	nd	nd	nd	nd	nd	nd	nd	nd	1.62	nd
Flonicamid	nd	nd	nd	nd	nd	nd	nd	nd	nd	nd	nd	nd	nd	nd	nd	nd	nd	nd	nd	nd
Florasulam	nd	nd	nd	nd	nd	nd	nd	nd	nd	nd	nd	nd	nd	nd	nd	nd	nd	nd	nd	nd

Fluazuron	nd	nd	nd	nd	nd	nd	nd	nd	nd	nd	nd	nd	nd	nd	nd	nd	nd	nd	nd	nd
Flubendiamide	nd	nd	nd	nd	nd	nd	nd	nd	nd	nd	nd	nd	nd	nd	nd	nd	nd	nd	nd	nd
Flufenacet	nd	nd	nd	nd	nd	nd	nd	nd	nd	nd	nd	nd	nd	nd	nd	nd	nd	nd	1.35	nd
Flufenoxuron	nd	nd	nd	nd	nd	nd	nd	nd	nd	nd	nd	nd	nd	nd	nd	nd	nd	nd	nd	nd
Fluometuron	nd	nd	nd	nd	nd	nd	nd	nd	nd	nd	nd	nd	nd	nd	nd	nd	nd	nd	nd	nd
Fluopicolide	nd	nd	nd	nd	nd	nd	nd	nd	nd	nd	nd	nd	nd	nd	nd	nd	nd	nd	1.28	nd
Fluopyram	nd	nd	nd	nd	nd	3.98	nd	nd	nd	nd	nd	5.71	nd	nd	nd	nd	1.10	nd	1.53	nd
Fluoxastrobin	nd	nd	nd	nd	nd	nd	nd	nd	nd	nd	nd	nd	nd	nd	nd	nd	nd	nd	1.53	nd
Fluquinconazole	nd	nd	nd	nd	nd	nd	nd	nd	nd	nd	nd	nd	nd	nd	nd	nd	nd	nd	nd	nd
Fluridone	nd	nd	nd	nd	nd	nd	nd	nd	nd	nd	nd	nd	nd	nd	nd	nd	nd	nd	1.49	nd
Flusilazole	nd	nd	nd	nd	nd	nd	nd	nd	nd	nd	nd	nd	nd	nd	nd	nd	nd	nd	1.65	nd
Flutolanil	nd	nd	nd	nd	nd	nd	nd	nd	nd	nd	nd	nd	nd	nd	nd	nd	nd	nd	1.80	nd
Flutriafol	nd	nd	nd	nd	nd	nd	nd	nd	nd	nd	nd	nd	nd	nd	nd	nd	nd	nd	nd	nd
Fluxapyroxad	nd	nd	nd	nd	nd	nd	nd	nd	nd	nd	nd	1.31	nd	nd	nd	nd	nd	nd	1.59	nd
Fonofos	nd	nd	nd	nd	nd	nd	nd	nd	nd	nd	nd	nd	nd	nd	nd	nd	nd	nd	nd	nd
Foramsulfuron	nd	nd	nd	nd	nd	nd	nd	nd	nd	nd	nd	nd	nd	nd	nd	nd	nd	nd	1.96	nd
Forchlorfenuron	nd	nd	nd	nd	nd	nd	nd	nd	nd	nd	nd	nd	nd	nd	nd	nd	nd	nd	nd	nd
Formetanate HCl	nd	nd	nd	nd	nd	nd	nd	nd	nd	nd	nd	nd	nd	nd	nd	nd	nd	nd	nd	nd
Fosthiazat	nd	nd	nd	nd	nd	nd	nd	nd	nd	nd	nd	nd	nd	nd	nd	nd	nd	nd	1.47	nd

Fuberidazole	nd	nd	nd	nd	nd	nd	nd	nd	nd	nd	nd	nd	nd	nd	nd	nd	nd	nd	1.60	nd
Furalaxyl	nd	nd	nd	nd	nd	nd	nd	nd	nd	nd	nd	nd	nd	nd	nd	nd	nd	nd	1.54	nd
Furathiocarb	nd	nd	nd	nd	nd	nd	nd	nd	nd	nd	nd	nd	nd	nd	nd	nd	nd	nd	nd	nd
Halofenozide	nd	nd	nd	nd	nd	nd	nd	nd	nd	nd	nd	nd	nd	nd	nd	nd	nd	nd	nd	nd
Haloxyfop-2-ethoxyethyl	nd	nd	nd	nd	nd	nd	nd	nd	nd	nd	nd	nd	nd	nd	nd	nd	nd	nd	1.48	nd
Haloxyfop-P-methyl	nd	nd	nd	nd	nd	nd	nd	nd	nd	nd	nd	nd	nd	nd	nd	nd	nd	nd	1.69	nd
Heptenophos	nd	nd	nd	nd	nd	nd	nd	nd	nd	nd	nd	nd	nd	nd	nd	nd	nd	nd	nd	nd
Hexaconazole	nd	nd	nd	nd	nd	nd	nd	nd	nd	nd	nd	nd	nd	nd	nd	nd	nd	nd	nd	nd
Hexaflumuron	nd	nd	nd	nd	nd	nd	nd	nd	nd	nd	nd	nd	nd	nd	nd	nd	nd	nd	nd	nd
Hexazionon(Velpar)	nd	nd	nd	nd	nd	nd	nd	nd	nd	nd	nd	nd	nd	nd	nd	nd	nd	nd	2.04	nd
Hydramethylnon	nd	nd	nd	nd	nd	nd	nd	nd	nd	nd	nd	nd	nd	nd	nd	nd	nd	nd	nd	nd
Hydroxycarbofuran	nd	nd	nd	nd	nd	nd	nd	nd	nd	nd	nd	nd	nd	nd	nd	nd	nd	nd	1.45	nd
Imazalil	nd	nd	nd	nd	nd	nd	nd	nd	nd	nd	nd	nd	nd	nd	nd	nd	nd	nd	nd	nd
Imazaquin	nd	nd	nd	nd	nd	nd	nd	nd	nd	nd	nd	nd	nd	nd	nd	nd	nd	nd	nd	nd
Imidacloprid	nd	nd	nd	32.21	4.11	27.85	nd	nd	nd	10.85	nd	37.13	nd	nd	1.85	nd	nd	nd	nd	nd
Indoxacarb	nd	nd	nd	nd	nd	nd	nd	nd	nd	nd	nd	nd	nd	nd	nd	nd	nd	nd	nd	nd
Ipconazole	nd	nd	nd	nd	nd	nd	nd	nd	nd	nd	nd	nd	nd	nd	nd	nd	nd	nd	nd	nd
Iprobenfos	nd	nd	nd	nd	nd	nd	nd	nd	nd	nd	nd	nd	nd	nd	nd	nd	nd	nd	nd	nd
Iprovalicarb	nd	nd	nd	nd	nd	nd	nd	nd	nd	nd	nd	nd	nd	nd	nd	nd	nd	nd	nd	nd

Isocarbamide	nd	nd	nd	nd	nd	nd	nd	nd	nd	nd	nd	nd	nd	nd	nd	nd	nd	nd	nd	nd
Isofenphos	nd	nd	nd	nd	nd	nd	nd	nd	nd	nd	nd	nd	nd	nd	nd	nd	nd	nd	nd	nd
Isofenphos-methyl	nd	nd	nd	nd	nd	nd	nd	nd	nd	nd	nd	nd	nd	nd	nd	nd	nd	nd	nd	nd
Isoprocarb	nd	nd	nd	nd	nd	nd	nd	nd	nd	nd	nd	nd	nd	nd	nd	nd	nd	nd	nd	nd
Isoprothiolane	nd	nd	nd	nd	nd	nd	nd	nd	nd	nd	nd	nd	nd	nd	nd	nd	nd	nd	nd	nd
Isoproturon	nd	nd	nd	1.90	nd	3.85	nd	nd	nd	nd	nd	10.89	nd	nd	nd	nd	nd	nd	1.95	nd
Isoxaflutole	nd	nd	nd	nd	nd	nd	nd	nd	nd	nd	nd	nd	nd	nd	nd	nd	nd	nd	nd	nd
Isoxathion	nd	nd	nd	nd	nd	nd	nd	nd	nd	nd	nd	nd	nd	nd	nd	nd	nd	nd	nd	nd
Ivermectin	nd	nd	nd	nd	nd	nd	nd	nd	nd	nd	nd	nd	nd	nd	nd	nd	nd	nd	nd	nd
izoxaben	nd	nd	nd	nd	nd	nd	nd	nd	nd	nd	nd	nd	nd	nd	nd	nd	nd	nd	nd	nd
Kresoxim-methyl	nd	nd	nd	nd	nd	nd	nd	nd	nd	nd	nd	nd	nd	nd	nd	nd	nd	nd	nd	nd
Lenacil	nd	nd	nd	nd	nd	nd	nd	nd	nd	nd	nd	nd	nd	nd	nd	nd	nd	nd	nd	nd
Lufenuron	nd	nd	nd	nd	nd	nd	nd	nd	nd	nd	nd	nd	nd	nd	nd	nd	nd	nd	nd	nd
Malaoxon	nd	nd	nd	nd	nd	nd	nd	nd	nd	nd	nd	nd	nd	nd	nd	nd	nd	nd	1.34	nd
Malathion	nd	nd	nd	nd	nd	nd	nd	nd	nd	nd	nd	nd	nd	nd	nd	nd	nd	nd	nd	nd
Mandipropamid	nd	nd	nd	nd	nd	nd	nd	nd	nd	nd	nd	nd	nd	nd	nd	nd	nd	nd	1.86	nd
Mecarbam	nd	nd	nd	nd	nd	nd	nd	nd	nd	nd	nd	nd	nd	nd	nd	nd	nd	nd	nd	nd
Mefenacet	nd	nd	nd	nd	nd	nd	nd	nd	nd	nd	nd	nd	nd	nd	nd	nd	nd	nd	1.58	nd
Mepanipyrim	nd	nd	nd	nd	nd	nd	nd	nd	nd	nd	nd	nd	nd	nd	nd	nd	nd	nd	nd	nd

Mephosfolan	nd	nd	nd	nd	nd	nd	nd	nd	nd	nd	nd	nd	nd	nd	nd	nd	nd	nd	1.38	nd
Mepronil	nd	nd	nd	nd	nd	nd	nd	nd	nd	nd	nd	nd	nd	nd	nd	nd	nd	nd	nd	nd
Mesotrione	nd	nd	nd	nd	nd	nd	nd	nd	nd	nd	nd	nd	nd	nd	nd	nd	nd	nd	nd	nd
Metaflumizone	nd	nd	nd	nd	nd	nd	nd	nd	nd	nd	nd	nd	nd	nd	nd	nd	nd	nd	nd	nd
Metalaxyl	nd	nd	nd	2.71	nd	nd	nd	nd	nd	3.69	nd	9.39	nd	nd	nd	nd	nd	nd	1.63	nd
Metamitron	nd	nd	nd	nd	nd	nd	nd	nd	nd	nd	nd	nd	nd	nd	nd	nd	nd	nd	nd	nd
Metazachlor	nd	nd	nd	nd	nd	nd	nd	nd	nd	nd	nd	nd	nd	nd	nd	nd	nd	nd	1.43	nd
Metconazole	nd	nd	nd	nd	nd	nd	nd	nd	nd	nd	nd	nd	nd	nd	nd	nd	nd	nd	nd	nd
Methabenzthiazuron	nd	nd	nd	nd	nd	nd	nd	nd	nd	nd	nd	nd	nd	nd	nd	nd	nd	nd	nd	nd
Methamidophos	nd	nd	nd	nd	nd	nd	nd	nd	nd	nd	nd	nd	nd	nd	nd	nd	nd	nd	nd	nd
Methidathion	nd	nd	nd	nd	nd	nd	nd	nd	nd	nd	nd	nd	nd	nd	nd	nd	nd	nd	nd	nd
Methiocarb	nd	nd	nd	nd	nd	nd	nd	nd	nd	nd	nd	nd	nd	nd	nd	nd	nd	nd	nd	nd
Methiocarb-sulfone	nd	nd	nd	nd	nd	nd	nd	nd	nd	nd	nd	nd	nd	nd	nd	nd	nd	nd	nd	nd
Methomyl	nd	nd	nd	nd	nd	nd	nd	nd	nd	nd	nd	nd	nd	nd	nd	nd	nd	nd	nd	nd
Methoprotryne	nd	nd	nd	nd	nd	nd	nd	nd	nd	nd	nd	nd	nd	nd	nd	nd	nd	nd	1.61	nd
Methoxyfenozide	nd	nd	nd	nd	nd	nd	nd	nd	nd	nd	nd	nd	nd	nd	nd	nd	nd	nd	1.30	nd
Metobromuron	nd	nd	nd	nd	nd	nd	nd	nd	nd	nd	nd	nd	nd	nd	nd	nd	nd	nd	1.55	nd
Metolachlor	7.55	2.25	1.79	1.94	nd	11.23	1.34	2.25	2.11	2.77	2.58	10.77	1.85	2.48	1.59	1.39	2.43	1.93	3.77	3.21
Metolcarb	nd	nd	nd	nd	nd	nd	nd	nd	nd	nd	nd	nd	nd	nd	nd	nd	nd	nd	nd	nd

Metosulam	nd	nd	nd	nd	nd	nd	nd	nd	nd	nd	nd	nd	nd	nd	nd	nd	nd	nd	2.03	nd
Metoxuron	nd	nd	nd	nd	nd	nd	nd	nd	nd	nd	nd	nd	nd	nd	nd	nd	nd	nd	nd	nd
Metrafenon	nd	nd	nd	nd	nd	nd	nd	nd	nd	nd	nd	nd	nd	nd	nd	nd	nd	nd	1.59	nd
Metsulfuron-methyl	nd	nd	nd	nd	nd	nd	nd	nd	nd	nd	nd	nd	nd	nd	nd	nd	nd	nd	1.64	nd
Mevinphos-Isomer	nd	nd	nd	nd	nd	nd	nd	nd	nd	nd	nd	nd	nd	nd	nd	nd	nd	nd	nd	nd
Mexacarbate	nd	nd	nd	nd	nd	nd	nd	nd	nd	nd	nd	nd	nd	nd	nd	nd	nd	nd	1.39	nd
Molinate	nd	nd	nd	nd	nd	nd	nd	nd	nd	nd	nd	nd	nd	nd	nd	nd	nd	nd	nd	nd
Monocrotophos	nd	nd	nd	nd	nd	nd	nd	nd	nd	nd	nd	nd	nd	nd	nd	nd	nd	nd	1.54	nd
Monolinuron	nd	nd	nd	nd	nd	nd	nd	nd	nd	nd	nd	nd	nd	nd	nd	nd	nd	nd	nd	nd
Moxidectin	nd	nd	nd	nd	nd	nd	nd	nd	nd	nd	nd	nd	nd	nd	nd	nd	nd	nd	nd	nd
Myclobutanil	nd	nd	nd	nd	nd	nd	nd	nd	nd	nd	nd	nd	nd	nd	nd	nd	nd	nd	nd	nd
Napropamide	nd	nd	nd	nd	nd	nd	nd	nd	nd	nd	nd	nd	nd	nd	nd	nd	nd	nd	nd	nd
Neburon	nd	nd	nd	nd	nd	nd	nd	nd	nd	nd	nd	nd	nd	nd	nd	nd	nd	nd	nd	nd
Nitenpyram	nd	nd	nd	nd	nd	nd	nd	nd	nd	nd	nd	nd	nd	nd	nd	nd	nd	nd	nd	nd
Nitralin	nd	nd	nd	nd	nd	nd	nd	nd	nd	nd	nd	nd	nd	nd	nd	nd	nd	nd	nd	nd
Norflurazon	nd	nd	nd	nd	nd	nd	nd	nd	nd	nd	nd	nd	nd	nd	nd	nd	nd	nd	nd	nd
Novaluron	nd	nd	nd	nd	nd	nd	nd	nd	nd	nd	nd	nd	nd	nd	nd	nd	nd	nd	nd	nd
Nuarimol	nd	nd	nd	nd	nd	nd	nd	nd	nd	nd	nd	nd	nd	nd	nd	nd	nd	nd	2.75	nd
Omethoate	nd	nd	nd	nd	nd	nd	nd	nd	nd	nd	nd	nd	nd	nd	nd	nd	nd	nd	1.35	nd

Oxadiazon	nd	nd	nd	nd	nd	nd	nd	nd	nd	nd	nd	nd	nd	nd	nd	nd	nd	nd	nd	nd
Oxadixyl	nd	nd	nd	nd	nd	nd	nd	nd	nd	nd	nd	nd	nd	nd	nd	nd	nd	nd	nd	nd
Oxamyl	nd	nd	nd	nd	nd	nd	nd	nd	nd	nd	nd	nd	nd	nd	nd	nd	nd	nd	nd	nd
Oxamyl-oxime	nd	nd	nd	nd	nd	nd	nd	nd	nd	nd	nd	nd	nd	nd	nd	nd	nd	nd	nd	nd
Oxydemeton-methyl	nd	nd	nd	nd	nd	nd	nd	nd	nd	nd	nd	nd	nd	nd	nd	nd	nd	nd	1.52	nd
Paclobutrazol	nd	nd	nd	nd	nd	nd	nd	nd	nd	nd	nd	nd	nd	nd	nd	nd	nd	nd	nd	nd
Paraoxon	nd	nd	nd	nd	nd	nd	nd	nd	nd	nd	nd	nd	nd	nd	nd	nd	nd	nd	1.54	nd
Paraoxon-ethyl	nd	nd	nd	nd	nd	nd	nd	nd	nd	nd	nd	nd	nd	nd	nd	nd	nd	nd	1.60	nd
Penconazole	nd	nd	nd	12.20	nd	2.51	nd	nd	nd	nd	nd	4.21	nd	nd	nd	nd	nd	nd	nd	nd
Penthiopyrad	nd	nd	nd	nd	nd	nd	nd	nd	nd	nd	nd	nd	nd	nd	nd	nd	nd	nd	1.44	nd
Pethoxamid	nd	nd	nd	nd	nd	nd	nd	nd	nd	nd	nd	nd	nd	nd	nd	nd	nd	nd	nd	nd
Phenmedipham	nd	nd	nd	nd	nd	nd	nd	nd	nd	nd	nd	nd	nd	nd	nd	nd	nd	nd	nd	nd
Phenthoate	nd	nd	nd	nd	nd	nd	nd	nd	nd	nd	nd	nd	nd	nd	nd	nd	nd	nd	nd	nd
Phorate-sulfone	nd	nd	nd	nd	nd	nd	nd	nd	nd	nd	nd	nd	nd	nd	nd	nd	nd	nd	nd	nd
Phorate-sulfoxide	nd	nd	nd	nd	nd	nd	nd	nd	nd	nd	nd	nd	nd	nd	nd	nd	nd	nd	nd	nd
Phosalone	nd	nd	nd	nd	nd	nd	nd	nd	nd	nd	nd	nd	nd	nd	nd	nd	nd	nd	nd	nd
Phosmet	nd	nd	nd	nd	nd	nd	nd	nd	nd	nd	nd	nd	nd	nd	nd	nd	nd	nd	nd	nd
Phosphamidon	nd	nd	nd	nd	nd	nd	nd	nd	nd	nd	nd	nd	nd	nd	nd	nd	nd	nd	nd	nd
Phoxim	nd	nd	nd	nd	nd	nd	nd	nd	nd	nd	nd	nd	nd	nd	nd	nd	nd	nd	nd	nd

Picoxystrobin	nd	nd	nd	nd	nd	nd	nd	nd	nd	nd	nd	nd	nd	nd	nd	nd	nd	nd	1.27	nd
Pinoxaden	nd	nd	nd	nd	nd	nd	nd	nd	nd	nd	nd	nd	nd	nd	nd	nd	nd	nd	1.37	nd
Piperonyl-butoxide	nd	nd	nd	nd	nd	nd	nd	nd	nd	nd	nd	nd	nd	nd	nd	nd	nd	nd	1.46	nd
Piperophos	nd	nd	nd	nd	nd	nd	nd	nd	nd	nd	nd	nd	nd	nd	nd	nd	nd	nd	1.31	nd
Pirimicarb	nd	nd	nd	1.69	nd	nd	nd	nd	nd	nd	nd	2.67	nd	nd	nd	nd	nd	nd	1.87	nd
Pirimicarb-desmethyl	nd	nd	nd	nd	nd	nd	nd	nd	nd	nd	nd	nd	nd	nd	nd	nd	nd	nd	1.52	nd
Pirimiphos-ethyl	nd	nd	nd	nd	nd	nd	nd	nd	nd	nd	nd	nd	nd	nd	nd	nd	nd	nd	1.55	nd
Pirimiphos-methyl	nd	nd	nd	nd	nd	nd	nd	nd	nd	nd	nd	nd	nd	nd	nd	nd	nd	nd	1.97	nd
Pirimiszulfuron-metil	nd	nd	nd	nd	nd	nd	nd	nd	nd	nd	nd	nd	nd	nd	nd	nd	nd	nd	nd	nd
Prochloraz	nd	nd	nd	nd	nd	nd	nd	nd	nd	nd	nd	nd	nd	nd	nd	nd	nd	nd	nd	nd
Prochloraz-desimidazole-amino	nd	nd	nd	nd	nd	nd	nd	nd	nd	nd	nd	nd	nd	nd	nd	nd	nd	nd	nd	nd
Promecarb	nd	nd	nd	nd	nd	nd	nd	nd	nd	nd	nd	nd	nd	nd	nd	nd	nd	nd	nd	nd
Prometon	nd	nd	nd	nd	nd	nd	nd	nd	nd	nd	nd	nd	nd	nd	nd	nd	nd	nd	1.79	nd
Prometryne	nd	nd	nd	nd	nd	7.87	nd	nd	nd	nd	nd	nd	nd	nd	nd	nd	nd	nd	1.53	nd
Propachlor	nd	nd	nd	nd	nd	nd	nd	nd	nd	nd	nd	nd	nd	nd	nd	nd	nd	nd	nd	nd
Propamocarb	nd	nd	nd	nd	nd	nd	nd	nd	nd	nd	nd	nd	nd	nd	nd	nd	nd	nd	nd	nd
Propanil	nd	nd	nd	nd	nd	nd	nd	nd	nd	nd	nd	nd	nd	nd	nd	nd	nd	nd	nd	nd
Propargite	nd	nd	nd	nd	nd	nd	nd	nd	nd	nd	nd	nd	nd	nd	nd	nd	nd	nd	nd	nd
Propetamphos	nd	nd	nd	nd	nd	nd	nd	nd	nd	nd	nd	nd	nd	nd	nd	nd	nd	nd	nd	nd

Propiconazole	nd	nd	nd	nd	nd	nd	2.22	1.84	1.74	nd	3.23	3.57	2.91	4.37	2.04	nd	nd	nd	nd	nd
Propoxur	nd	nd	nd	nd	nd	nd	nd	nd	nd	nd	nd	nd	nd	nd	nd	nd	nd	nd	1.59	nd
Propyzamide	nd	nd	nd	nd	nd	nd	nd	nd	nd	nd	nd	nd	nd	nd	nd	nd	nd	nd	nd	nd
Prosulfocarb	nd	nd	nd	nd	nd	nd	nd	nd	nd	nd	nd	nd	nd	nd	nd	nd	nd	nd	1.66	nd
Pymetrozine	nd	nd	nd	nd	nd	nd	nd	nd	nd	nd	nd	nd	nd	nd	nd	nd	nd	nd	1.24	nd
Pyracarbolid	nd	nd	nd	nd	nd	nd	nd	nd	nd	nd	nd	nd	nd	nd	nd	nd	nd	nd	1.04	nd
Pyraclostrobin	nd	nd	nd	nd	nd	nd	nd	nd	nd	nd	nd	nd	nd	nd	nd	nd	nd	nd	1.52	nd
Pyraflufen-ethyl	nd	nd	nd	nd	nd	nd	nd	nd	nd	nd	nd	nd	nd	nd	nd	nd	nd	nd	nd	nd
Pyrazophos	nd	nd	nd	nd	nd	nd	nd	nd	nd	nd	nd	nd	nd	nd	nd	nd	nd	nd	nd	nd
Pyridafol	nd	nd	nd	nd	nd	nd	nd	nd	nd	nd	nd	nd	nd	nd	nd	nd	nd	nd	nd	nd
Pyridaphenthion	nd	nd	nd	nd	nd	nd	nd	nd	nd	nd	nd	nd	nd	nd	nd	nd	nd	nd	1.28	nd
Pyrifenox	nd	nd	nd	nd	nd	nd	nd	nd	nd	nd	nd	nd	nd	nd	nd	nd	nd	nd	nd	nd
Pyrimethanil	nd	nd	nd	nd	nd	nd	nd	nd	nd	nd	nd	nd	nd	nd	nd	nd	nd	nd	nd	nd
Pyroquilon	nd	nd	nd	nd	nd	nd	nd	nd	nd	nd	nd	nd	nd	nd	nd	nd	nd	nd	nd	nd
Quinalphos	nd	nd	nd	nd	nd	nd	nd	nd	nd	nd	nd	nd	nd	nd	nd	nd	nd	nd	nd	nd
Sebuthylazin	nd	nd	nd	nd	nd	nd	nd	nd	nd	nd	nd	nd	nd	nd	nd	nd	nd	nd	nd	nd
Secbumeton	nd	nd	nd	nd	nd	nd	nd	nd	nd	nd	nd	nd	nd	nd	nd	nd	nd	nd	2.22	nd
Silthiofam	nd	nd	nd	nd	nd	nd	nd	nd	nd	nd	nd	nd	nd	nd	nd	nd	nd	nd	nd	nd
Simazine	nd	nd	nd	nd	nd	nd	nd	nd	nd	nd	nd	nd	nd	nd	nd	nd	nd	nd	nd	nd

Simetryn	nd	nd	nd	nd	nd	nd	nd	nd	nd	nd	nd	nd	nd	nd	nd	nd	nd	nd	1.37	nd
Spinetoram	nd	nd	nd	nd	nd	nd	nd	nd	nd	nd	nd	nd	nd	nd	nd	nd	nd	nd	nd	nd
Spiromesifen	nd	nd	nd	nd	nd	nd	nd	nd	nd	nd	nd	nd	nd	nd	nd	nd	nd	nd	nd	nd
Spirotetramat	nd	nd	nd	nd	nd	nd	nd	nd	nd	nd	nd	nd	nd	nd	nd	nd	nd	nd	nd	nd
Spiroxamine-Isomer	nd	nd	nd	nd	nd	nd	nd	nd	nd	nd	nd	nd	nd	nd	nd	nd	nd	nd	2.72	1.23
Sulfometuron-methyl	nd	nd	nd	nd	nd	nd	nd	nd	nd	nd	nd	nd	nd	nd	nd	nd	nd	nd	nd	nd
Sulfosulfuron	nd	nd	nd	nd	nd	nd	nd	nd	nd	nd	nd	nd	nd	nd	nd	nd	nd	nd	nd	nd
Tebuconazole	nd	nd	nd	5.62	nd	nd	nd	nd	nd	nd	nd	15.19	nd	nd	nd	nd	nd	nd	nd	nd
Tebufenozide	nd	nd	nd	nd	nd	nd	nd	nd	nd	nd	nd	nd	nd	nd	nd	nd	nd	nd	nd	nd
Tebufenpyrad	nd	nd	nd	nd	nd	nd	nd	nd	nd	nd	nd	nd	nd	nd	nd	nd	nd	nd	nd	nd
Tebutam	nd	nd	nd	nd	nd	nd	nd	nd	nd	nd	nd	nd	nd	nd	nd	nd	nd	nd	nd	nd
Tebuthiuron	nd	nd	nd	nd	nd	nd	nd	nd	nd	nd	nd	nd	nd	nd	nd	nd	nd	nd	1.28	nd
Terbufos-sulfoxide	nd	nd	nd	nd	nd	nd	nd	nd	nd	nd	nd	nd	nd	nd	nd	nd	nd	nd	nd	nd
Terbumeton	nd	nd	nd	nd	nd	nd	nd	nd	nd	nd	nd	nd	nd	nd	nd	nd	nd	nd	1.88	nd
Terbuthylazine-desethyl	nd	nd	nd	18.13	nd	1.91	1.11	1.42	1.70	3.06	1.39	1.75	1.21	1.49	nd	1.07	1.20	1.16	2.54	1.75
Terbutryn	nd	nd	nd	nd	nd	5.84	nd	1.66	nd	5.12	1.00	6.15	nd	1.00	nd	nd	nd	nd	nd	nd
Tetrachlorvinphos	nd	nd	nd	nd	nd	nd	nd	nd	nd	nd	nd	nd	nd	nd	nd	nd	nd	nd	nd	nd
Tetraconazole	nd	nd	nd	nd	nd	6.84	nd	nd	nd	nd	nd	nd	nd	nd	nd	nd	nd	nd	nd	nd
Thiabendazole	1.50	12.62	6.44	3.74	nd	42.36	8.19	16.44	10.33	43.88	11.95	27.94	10.08	15.04	6.52	9.23	10.55	10.17	14.44	11.25

Thiacloprid	nd	nd	nd	nd	nd	nd	nd	nd	nd	nd	nd	nd	nd	nd	nd	nd	nd	nd	1.55	nd
Thiamethoxam	nd	nd	nd	nd	nd	nd	nd	nd	nd	nd	nd	8.07	3.58	nd	nd	nd	nd	nd	nd	nd
Thidiazuron	nd	nd	nd	nd	nd	nd	nd	nd	nd	nd	nd	nd	nd	nd	nd	nd	nd	nd	nd	nd
Thifensulfuron-methyl	nd	nd	nd	nd	nd	nd	nd	nd	nd	nd	nd	nd	nd	nd	nd	nd	nd	nd	1.58	nd
Thiodicarb	nd	nd	nd	nd	nd	nd	nd	nd	nd	nd	nd	nd	nd	nd	nd	nd	nd	nd	nd	nd
Thiofanox-sulfoxide	nd	nd	nd	nd	nd	nd	nd	nd	nd	nd	nd	nd	nd	nd	nd	nd	nd	nd	nd	nd
Thiophanate	nd	nd	nd	nd	nd	nd	nd	nd	nd	nd	nd	nd	nd	nd	nd	nd	nd	nd	nd	nd
Thiophanate-methyl	nd	nd	nd	nd	nd	nd	nd	nd	nd	nd	nd	nd	nd	nd	nd	nd	nd	nd	nd	nd
Tralkoxydim	nd	nd	nd	nd	nd	nd	nd	nd	nd	nd	nd	nd	nd	nd	nd	nd	nd	nd	nd	nd
Triadimefon	nd	nd	nd	nd	nd	nd	nd	nd	nd	nd	nd	nd	nd	nd	nd	nd	nd	nd	nd	nd
Triadimenol	nd	nd	nd	nd	nd	nd	nd	nd	nd	nd	nd	nd	nd	nd	nd	nd	nd	nd	nd	nd
Triasulforon	nd	nd	nd	nd	nd	nd	nd	nd	nd	nd	nd	nd	nd	nd	nd	nd	nd	nd	2.61	nd
Triazophos	nd	nd	nd	nd	nd	nd	nd	nd	nd	nd	nd	nd	nd	nd	nd	nd	nd	nd	1.53	nd
Tricyclazole	nd	nd	nd	nd	nd	nd	nd	nd	nd	nd	nd	nd	nd	nd	nd	nd	nd	nd	1.53	nd
Trietazin	nd	nd	nd	nd	nd	nd	nd	nd	nd	nd	nd	nd	nd	nd	nd	nd	nd	nd	1.19	1.07
Trifloxystrobin	nd	nd	nd	nd	nd	nd	nd	nd	nd	nd	nd	nd	nd	nd	nd	nd	nd	nd	nd	nd
Triflumizole	nd	nd	nd	nd	nd	nd	nd	nd	nd	nd	nd	nd	nd	nd	nd	nd	nd	nd	nd	nd
Triflumuron	nd	nd	nd	nd	nd	nd	nd	nd	nd	nd	nd	nd	nd	nd	nd	nd	nd	nd	nd	nd
Triflusulfuron-methyl	nd	nd	nd	nd	nd	nd	nd	nd	nd	nd	nd	nd	nd	nd	nd	nd	nd	nd	1.57	nd

Trimethylphenyl-methylcarbamate	nd	nd	nd	nd	nd	nd	nd	nd	nd	nd	nd	nd	nd	nd	nd	nd	nd	nd	nd	nd
Triticonazole	nd	nd	nd	nd	nd	nd	nd	nd	nd	nd	nd	nd	nd	nd	nd	nd	nd	nd	nd	nd
Vamidothion	nd	nd	nd	nd	nd	nd	nd	nd	nd	nd	nd	nd	nd	nd	nd	nd	nd	nd	1.54	nd
Vamidothion-sulfoxide	nd	nd	nd	nd	nd	nd	nd	nd	nd	nd	nd	nd	nd	nd	nd	nd	nd	nd	nd	nd
Zoxamide	nd	nd	nd	nd	nd	nd	nd	nd	nd	nd	nd	nd	nd	nd	nd	nd	nd	nd	nd	nd
**SUM (above 10 ng/L)**	**<10**	**28.0**	**<10**	**163.2**	**<10**	**92.9**	**<10**	**30.5**	**20.7**	**85.8**	**28.8**	**145.9**	**20.2**	**31.3**	**31.9**	**10.3**	**22.5**	**22.5**	**33.2**	**25.0**
**SUM (all results)**	**11.8**	**34.5**	**19.5**	**212.7**	**15.7**	**159.0**	**30.0**	**45.8**	**31.3**	**131.4**	**45.2**	**219.1**	**34.7**	**46.1**	**58.4**	**26.2**	**36.5**	**30.8**	**244.5**	**41.5**
